# The Potential of Thermomechanical and Thermochemical Processes to Enable Sustainable Household Sanitation

**DOI:** 10.1021/acs.est.5c15639

**Published:** 2026-01-15

**Authors:** Zixuan Wang, Jianan Feng, Buai Shi, Johanna Arita Mendoza, Xinyi Zhang, Nina Trousdale, Roland D. Cusick, Shannon Yee, Jeremy S. Guest

**Affiliations:** † The Grainger College of Engineering, Department of Civil and Environmental Engineering, Newmark Civil Engineering Laboratory, 14589 University of Illinois Urbana-Champaign, 205 N. Mathews Avenue, Urbana, Illinois 61801, United States; ‡ George W. Woodruff School of Mechanical Engineering, Georgia Institute of Technology, Atlanta, Georgia 30332, United States; § Institute for Sustainability, Energy, and Environment, University of Illinois Urbana-Champaign, 1101 W. Peabody Drive, Urbana, Illinois 61801, United States

**Keywords:** household-level non-sewered sanitation, mechanical dewatering, supercritical water oxidation, life cycle assessment (LCA), techno-economic analysis (TEA)

## Abstract

Biological processes underpin centralized wastewater treatment but are difficult to deploy at a small scale. Thermomechanical and thermochemical approaches could enable household-level sanitation, yet their economic and environmental potential remains unclear. We assessed two prototype household reinvented toilets (HRTs), with either pasteurization mechanical dewatering (PMD) and supercritical water oxidation (SCWO) treatment processes, using integrated process simulation, techno-economic analysis, and life cycle assessment under uncertainty. The total annualized expenditures (including capital and operating) are 1.41–1.87 (5th to 95th percentiles) and 1.85–2.45 USD·cap^–1^·day^–1^ for PMD and SCWO, respectively, placing both at the high end of global centralized treatment prices. The life cycle greenhouse gas (GHG) emissions span 321–452 and 362–520 kg CO_2_-eq·cap^–1^·year^–1^ for PMD and SCWO, respectively, with the grid electricity contributing 87–90% in both HRTs. Poor solid–liquid separation disproportionately increases costs and GHG emissions for SCWO relative to PMD. In the short term, optimizing a few levers—number of users, flush water volume, and the detailed design of the SCWO unit—can significantly reduce cost and emissions. In the long term, operating at maximum efficiency reduces both cost and emissions by approximately 70%. Deployment in locations with low wage, low-carbon electricity, low price levels, and large household sizes offers the greatest potential, positioning HRTs as viable advanced decentralized sanitation options in specialized settings.

## Graphical abstract

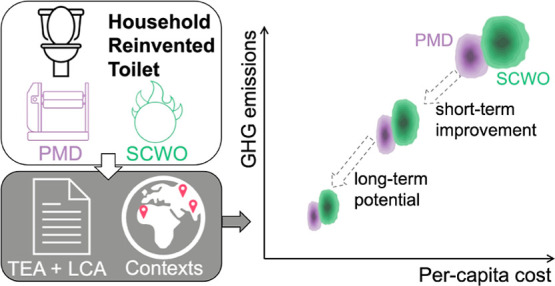

## 1 Introduction

Centralized urban sanitation systems represent one of the most significant engineering achievements of the 20th century, effectively preventing disease transmission and extending the life expectancy.^1^ These conventional systems—relying heavily on extensive underground pipe networks—require large-scale infrastructure and substantial upfront capital investment,^2–4^ and their global expansion has slowed.^5^ In developed economies, the cost of delivering centralized sanitation services continues to rise due to increasing topographic complexity, urban sprawl that extends pipe length, and the maintenance and renewal of aging infrastructure.^6–8^ Since 2020, more people globally have used non-sewered sanitation (NSS) systems than sewered systems.^9^ NSS systems such as septic tanks, improved latrines, and advanced decentralized wastewater treatment systems, can be deployed rapidly as small, modular installations that meet local or regional needs without mega-infrastructure investment.^5^ They can add flexible capacity to absorb short-term load spikes,^10^ provide onsite water reuse and nutrient recycling,^11,12^ and have demonstrated resilience to climate change-driven natural disasters.^13^ These advantages have attracted growing investment from both public institutions and the private sector, with projections anticipating a global NSS market growth of 13.6% in 2024.^14–16^

Conventional NSS technologies often use biological or natural treatment systems—such as anaerobic baffled reactors, aerated tanks, or drain fields—to reduce pathogens, chemical oxygen demand (COD), and nutrients (including nitrogen and phosphorus) in human excreta.^17–19^ Recent research funded by the Gates Foundation has focused on community reinvented toilets (CRTs) with enhanced liquid treatment, including biological nutrient removal sequencing batch reactors, anaerobic membrane bioreactors, ion exchange systems, and granular activated carbon columns.^20–23^ Some of these systems have undergone field testing in South Africa and India and met ISO 30500 performance criteria for *E. coli*, COD, total suspended solids (TSS), and total nitrogen (TN) removal.^21,24–26^ However, these systems still fall short of ISO 30500 standards for total phosphorus (TP) removal (>80% reduction), and the accumulated solids still require offsite treatment. Additionally, these CRTs are typically designed to serve populations of 30–800, making them unsuitable for remote off-grid households or compact spaces.

To address the sanitation gap and integrate on-site solids treatment, household reinvented toilets (HRTs) are designed to process blackwater—a mixture of urine, feces, flush water, and toilet paper—from a single household (typically 1–12 users). These toilets increasingly combine thermomechanical, thermochemical, and membrane separation technologies to minimize the spatial footprint. Recent advances include membrane separation for liquid treatment,^27^ while the spectrum of solid treatment technologies (Table S1) range from thermomechanical methods, such as pasteurization followed by mechanical dewatering (PMD),^28^ Archimedes-screw-based dewatering followed by combustion,^29^ and gravity-based drying followed by smoldering,^30^ to thermochemical methods, such as supercritical water oxidation (SCWO)^31^ and hydrothermal carbonization.^32^ PMD- and SCWO-based HRTs represent the technological end-members of these pathways, with PMD operating at the low-temperature–pressure ranges (ca. 90 °C, 1 bar) and SCWO operating at the high-temperature–pressure ranges (ca. 374 °C, 220 bar). Both configurations share a similar liquid treatment system and reportedly meet all ISO 30500 liquid-treatment criteria,^33^ positioning them as representative cases of thermomechanical- and thermochemical-based HRTs, respectively. Although both HRTs have undergone field testing,^34^ their financial feasibility and life cycle greenhouse gas (GHG) emissions are not well-characterized. Previous studies have demonstrated the economic and environmental sustainability of CRTs serving 100 users,^35^ yet HRTs are often associated with higher per-capita cost and/or emissions as they employ more advanced treatment while serving fewer users. Identifying key technological improvements, context-specific deployment strategies, and performance limits of these two HRTs is therefore essential to elucidate the economic and environmental opportunity space of HRTs and to inform their future research, development, and deployment (RD&D).

This study aims (i) to characterize the economic performance, environmental impacts, and maximum efficiency limits of thermomechanical and thermochemical HRTs, using PMD- and SCWO- HRTs as representative cases, respectively, and (ii) to prioritize technological improvements and contextual features that enhance the economic and environmental sustainability of HRTs. To achieve these goals, we used QSDsan^36,37^ (an open-source Python platform for sanitation and resource recovery system analysis) to develop process models of PMD- and SCWO- HRTs and to quantify per-capita costs and GHG emissions through techno-economic analysis (TEA) and life cycle assessment (LCA) under uncertainty. Process designs and technical assumptions were informed by published patents and communication with inventors.^28,38–41^ To guide future RD&D of HRTs, we conducted sensitivity analyses to identify key performance drivers and evaluated the performance of PMD- and SCWO-HRTs with contextual parameters from across 77 countries.^42^ Overall, this study provides insights into the financial and environmental viability of nonbiological HRT systems and highlights their development pathways and context-specific implementation strategies to advance NSS.

## 2 Methods

### 2.1 System Flow and Process Model

The design of PMD- and SCWO-HRTs was based on the processes described in published patents.^28,38–41^ Both HRTs were designed to treat blackwater from 6 users and comprised a squatting flush toilet (the user interface; also known as the frontend) system, a solids pretreatment system, a liquid treatment system, a solids treatment system, and a drying system (Figures 1 and S2–S4). Mixed blackwater first enters the solids pretreatment system—vacuum pump, wedge-wire filter vacuum tank solids separator, and belt separator—which produces a sludge stream at 84% moisture (range 75–90%) and a liquor with <1% suspended solids. The liquor then passes through reservoir tanks, ultrafiltration (UF), and reverse osmosis (RO) to meet ISO 30500 effluent standards. The frontend and liquid treatment system are functionally identical for the two HRT configurations.

**Figure 1:**
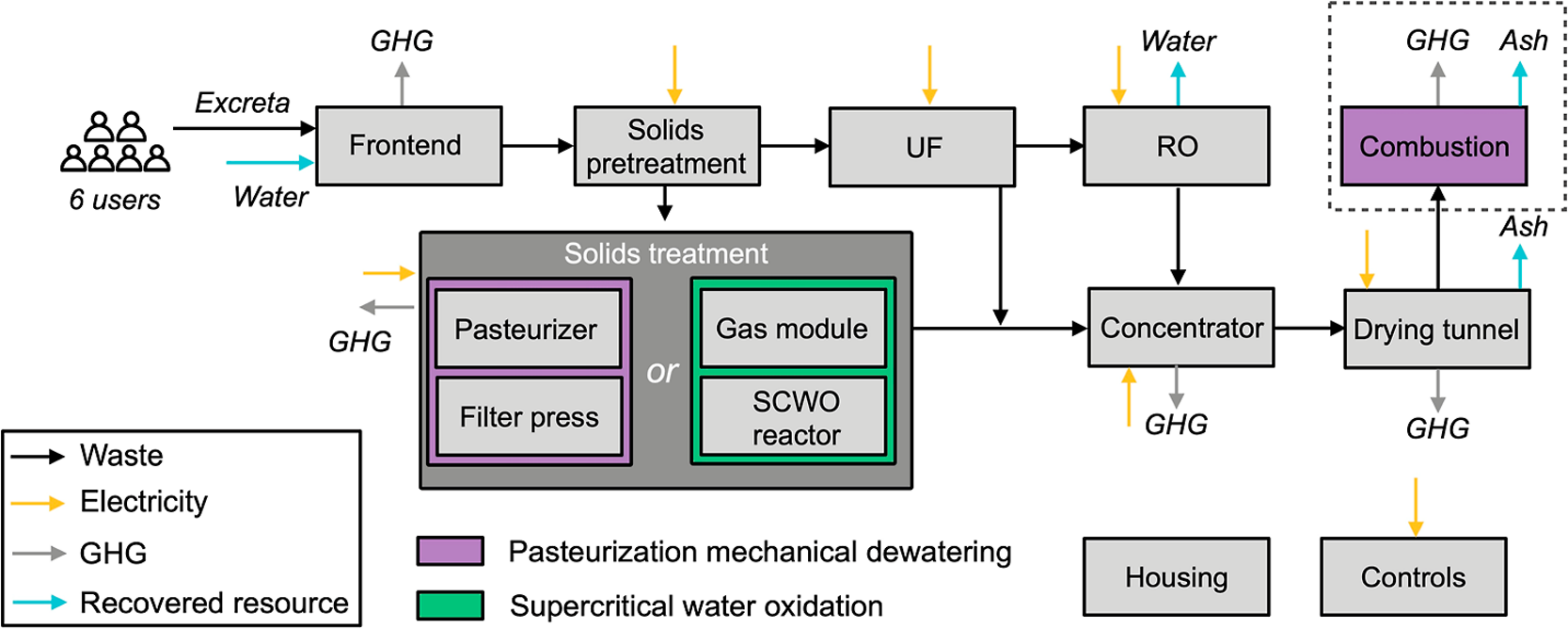
Process flow diagram and system boundary for the pasteurization mechanical dewatering (PMD) and the supercritical water oxidation (SCWO) toilets analyzed in this study. Gray boxes represent overlapping units between two types of toilets. Purple boxes represent units unique to PMD, and the green box represents the SCWO unit unique to SCWO. The housing and controls are not directly dependent upon the selected treatment train but are included in the costs and emissions analyses. The combustion unit (dotted black box) is included for modeling consistency but is not required in the field test for the PMD toilet. The main inputs include liquid and solid wastes (black arrows) and electricity (yellow arrows). The main outputs include water permeating from the reverse osmosis (RO) unit that is recycled for toilet flushing, water vapor and other trace gases (e.g., SO_*x*_, NO_*x*_, etc., not shown), direct greenhouse gas (GHG) emissions (N_2_O and CH_4_, gray arrows), and nutrients (nitrogen, phosphorus, and potassium) recovered from the ash product (blue arrows). UF stands for ultrafiltration.

The solids treatment system diverges thereafter. For the PMD HRT, compressional rheology studies suggest that decentralized solids dewatering via PMD is more efficient than transport and offsite treatment.^43^ The solids treatment system in the PMD HRT includes a homogenizer, a pasteurizer, and a filter press. The solids are pasteurized inline at 90 °C for 10 min. The volume of pasteurized solid waste is reduced through a mechanical dewatering press, lowering the moisture content to about 56% (range 28–84%). A drying system consists of an evaporative concentrator and a drying tunnel, which is used to further evaporate additional moisture, including the concentrated RO brine paste and the dewatered solids cake. The final dried cake is ready for disposal and can be combusted externally if desired. For a consistent final product, we included the combustor in the analysis and assumed that each external combustor can serve 60 users. For the SCWO HRT, the solid treatment system includes a homogenizer, an air compression system, and a SCWO reactor. SCWO kills pathogens, mineralizes organics, and recovers chemical energy from the solid waste at 408 °C (range 367–450 °C) and 221 bar. The SCWO HRT’s drying system, which similarly consists of an evaporative concentrator and a drying tunnel, vaporizes water from RO brine and the SWCO effluent to produce ash. Both HRTs can recover excess clean water from urine for flushing and discharge and mineral ash that contains macronutrients, including nitrogen (N), phosphorus (P), and potassium (K).

The blackwater components modeled in our system include H_2_O, NH_3_, non-NH_3_ nitrogen, dissolved organic carbon, particulate organic carbon, P, K, Mg, Ca, N_2_O, CH_4_, and unspecified soluble solids. The quantity of each component in the blackwater was estimated based on dietary intake and the fraction of intake excreted in urine and feces, a method established in previous work (Table S2).^44,45^ We used G2RT patents, similar units previously modeled in QSDsan, literature, technical manuals, or vendor web sites (in descending order of preference, as applied throughout this manuscript) to gather measured performance data to develop process models (Table S3). These data were parameterized into mass flow parameters (e.g., removal efficiencies) to calculate the individual component’s mass in the output streams. Data uncertainty was represented with probability distributions and propagated through uncertainty and sensitivity analyses.

### 2.2 Techno-Economic Analysis (TEA)

We conducted a discounted annual cash flow analysis (Figure S6) to determine the daily per-capita cost (USD·capita­(cap)^−1^·day^–1^) of PMD and SCWO HRTs with a basis year of 2020. Cash flows included capital expenditure (CAPEX), maintenance costs, electricity costs, labor costs, and revenue from recovered resources (e.g., NPK fertilizers and flush water). CAPEX represented a fixed capital investment for each unit and was derived from three main sources: the bill of materials from similar units previously modeled in QSDsan, vendor quotes, and the summation of individual parts (Table S5). To account for other costs incurred through the supply chain (e.g., fitting, assembly) when using the summation method, we applied a miscellaneous cost ratio on top of the purchase cost of individual parts (Figure S6). The manufacturing economy of scale was projected to reach an assumed production volume of 100,000 units by using a learning curve function (Figure S5) consistent with past studies.^35,46^ Maintenance costs included the replacement of parts and materials throughout the technology’s lifetime. Annual replacement cost was determined based on prior analysis of similar processes in QSDsan, manufacturer manuals, or an assumed annual replacement ratio (Table S4). Electricity consumption estimates were based on prior analysis of similar processes, literature values, or theoretical thermodynamic- and efficiency-based calculations (Table S6). Annual labor hours for the maintenance of each unit were based on prior analysis of similar processes, reported practices, or assumptions (Table S7). To estimate electricity and labor costs under both the general and targeted improvement scenarios, we applied the 50%–150% uncertainty range around the global average electricity unit price and labor wage (Table S4). Resource recovery offsets costs by generating revenue from nitrogen, phosphorus, and potassium in the final ash product as a replacement of fertilizer and from recycled water as a replacement of flushing water. The economic value was estimated using fertilizer and freshwater selling prices (Table S4), with a price factor of 0.25 applied to account for reduced market preference for recycled materials, an approach used in previous publications.^35,47^

### 2.3 Life Cycle Assessment (LCA)

The LCA impact category used in this study was life cycle GHG emissions, with all sources of GHGs normalized to CO_2_ equivalents on a 100 year time horizon. The functional unit was the provision of sanitation for one person for one year (CO_2_-eq·cap^–1^·year^–1^) using HRTs serving 6 people. The system boundary included all materials used for construction, electricity consumption, direct emissions, and recovery products during operation over a lifetime of 10 years (Figures 1 and S8). When the masses of construction material were not available, we estimated material masses using material density and volume or by referencing similar units with known weights from an external vendor. The life cycle inventory data and impact factors for construction materials, electricity, material inputs, and direct emission were gathered from Ecoinvent v3.8.^48^ GHG impact assessment followed the U.S. EPA’s Tool for the Reduction and Assessment of Chemicals and Other Environmental Impacts (TRACI) methodology (2007 version).^49^ For the baseline (general scenario), the rest-of-the-world electricity carbon intensity (CI) was used. Emissions of CH_4_ and N_2_O were estimated based on COD and nitrogen content, respectively, in the mixed waste across various processes throughout the treatment train, including storage at the frontend, concentrating and drying at the concentrator and drying tunnel, oxidation at the SCWO reactor, and combustion at the PMD combustor unit (Table S8).

### 2.4 Uncertainty and Sensitivity Analyses

To quantify uncertainty in the daily per-capita cost and the annual per-capita GHG emissions, we defined probability distributions for 445 and 429 input parameters to model the PMD and SCWO HRTs, respectively, and conducted Monte Carlo simulations (repeated random sampling of inputs) with 10,000 iterations for each analysis. Stratified random sampling of input parameters was performed using the Latin hypercube sampling method. Given that our input parameters were from various sources and had epistemic uncertainties, we applied selection criteria derived from Feng et al. to determine the numerical range and probability distribution type (Section S5).^50^ Due to the inherent high impact of user count and system lifetime on daily cost and annual per-capita GHG emissions, these two parameters were excluded from the uncertainty and sensitivity analyses and were analyzed individually in Section 3.4. To identify key input parameters (e.g., emission factors, removal efficiencies, nitrogen speciation fractions, purchase costs) influencing TEA and LCA results, we conducted a sensitivity analysis using Spearman’s rank-order correlation. Parameters with *p*-values < 0.05 and absolute Spearman’s ρ-values >0.2 (per-capita costs) or >0.1 (per-capita GHG emissions) were identified as priorities for targeted research, development, and deployment. We relaxed the |ρ| cutoff for emissions to allow secondary but still actionable parameters to be identified as coefficient values were compressed toward zero by a single dominant driver (see Section 3.3).

### 2.5 Improvement Scenarios Analysis

To explore short- and long-term pathways, we evaluated two improvement scenarios against the general (baseline) case. The baseline represents the default design capacity (e.g., serving six users) and uses global average values for contextual parameters, such as the wage and electricity price (Table S4). The targeted-improvement scenario represents near-term measures to cut costs and emissions. We first ranked inputs by Spearman’s ρ and then adjusted high-leverage and controllable parameters one at a time to identify their optimal values. We next applied these optimized values simultaneously to compute system performance under the targeted improvement scenario (Tables S9 and S10). The maximum efficiency scenario reflects a long-term goal: in addition to the targeted improvement, all parameters related to energy use, energy efficiency, and labor hours were adjusted to ideal values (Tables S9 and S11), and the ideal values were used as input to estimate costs and emissions.

### 2.6 Location-Specific Analysis

To assess how local conditions affect the financial viability and GHG emissions of the implementation of HRTs, we conducted a location-specific analysis with country-level data sets from 77 countries. The list of countries can be found in a previous study.^42^ Among the 450+ input parameters, contextual parameters including CI of a country’s electricity source, electricity unit price, dietary intake, household food waste, household size, labor wage, and price level ratio (ratio of local price levels to price levels in United States) were collected from country-level databases (Figure S16).^42^ The price level was used to adjust the material and component replacement costs to reflect local sourcing expenses. Additionally, the number of users per HRT was set based on the average household size in each country. The global range of tariffs charged by centralized water resource recovery facilities (WRRFs) were compiled from the International Benchmarking Network by a previous work.^51,52^

## 3 Results and Discussion

### 3.1 Solids Diversion and Membrane Processes Are Essential in Meeting the Effluent Target

For a household of six users and a range of potential diets (Table S2), the system treats 69.5 [31.9–132] kg·day^–1^ water, 0.228 [0.0915–0.370] kg-COD·day^–1^ organic carbon (measured as COD), 0.222 [0.113–0.339] kg·day^–1^ TSS, 0.0492 [0.0402–0.0589] kg·day^–1^ total N, 0.00690 [0.00348–0.0110] kg·day^–1^ total P, and 0.0134 [0.0102–0.0173] kg·day^–1^ total K (values in square brackets represent the 5th to 95th percentiles). Most COD and suspended solids are removed from the liquid phase by solid pretreatment and membrane operations. In both PMD and SCWO HRTs, solids pretreatment diverts 80.9% to 86.2% of the influent organic carbon and 2.74% to 2.92% of water to the solids treatment system (Figure 2). Clarified liquid from solids treatment is sent to the UF unit, which rejects 95% [75–100%] of the remaining particulate carbon in the liquid stream but allows 65% [60–70%] of soluble carbon to reach RO. Consequently, 22.2% [14.5–40.1%] (PMD) and 20.6% [13.8–36.3%] (SCWO) of the initial carbon arrives at the RO unit; almost all is subsequently retained in the RO brine and sent to drying (Figure 2). Final RO permeates contain 60.9 [19.9–176.0] mg·L^–1^ (PMD) and 60.3 [19.6–174] mg·L^–1^ (SCWO) COD and 0.511 [0.0360–2.99] mg·L^–1^ (PMD) and 0.193 [0.0108–1.40] mg·L^–1^ (SCWO) TSS, aligning with experimental reports of 33–87 mg·L^–1^ COD and TSS below a 1 mg·L^–1^ detection limit in the liquid effluent.^33^ RO also plays a critical role in removing soluble nutrients from the liquid stream, achieving nitrogen and phosphorus removal efficiencies of 82.3% [77.2–89.1%] and 94.7% [91.3–97.6%], respectively, for the PMD HRT and 82.5% [77.5–89.2%] and 94.8% [91.5–97.7%], respectively, for the SCWO HRT—values close to experimental observations of 87% TN and 99.9% TP removal.^33^ Both HRTs largely comply with ISO 30500: 91.6% of simulations achieve COD < 150 mg·L^–1^, and all simulations achieve TSS < 10 mg·L^–1^ and over 70% TN and 80% TP removal (Figure 2).

**Figure 2:**
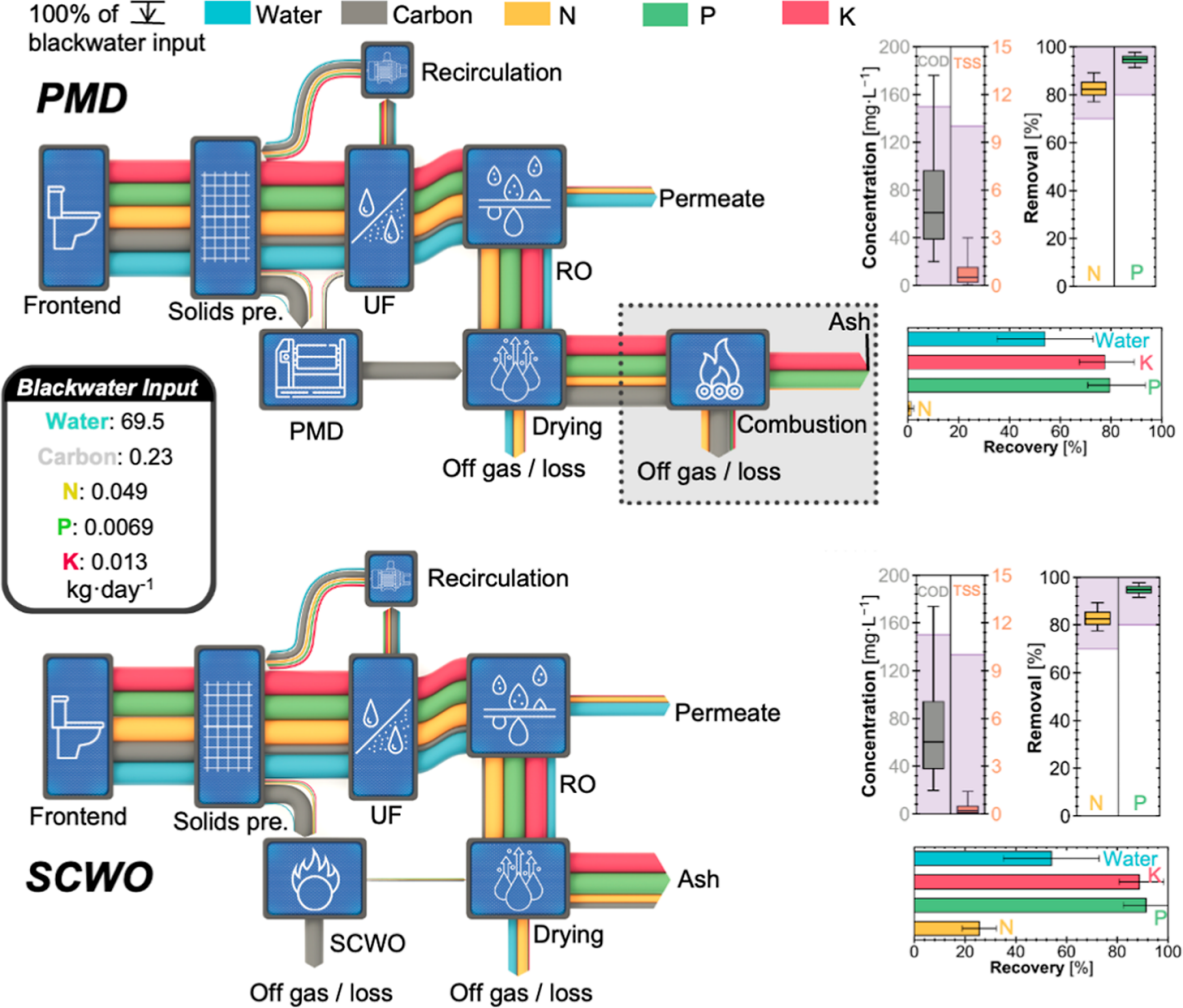
Mass flow, treatment performance, and resource recovery potential of pasteurization mechanical dewatering (PMD, top) and supercritical water oxidation (SCWO, bottom) toilet systems treating blackwater. Sankey diagrams illustrate median mass flows (kg·h^–1^) of water (blue), carbon (gray), nitrogen (N, yellow), phosphorus (P, green), and potassium (K, red) through each unit operation. Flow widths are scaled to the percentage of each element’s initial input. Flow direction is from left to right, except for ultrafiltration (UF) recirculating to solids pretreatment (Solids pre.). The combustion unit (dotted black box) is included for modeling consistency but was not used in PMD toilet field testing. The permeate from reverse osmosis (RO) is returned to the frontend for flushing but is not shown above for clarity. Box plots on the right show chemical oxygen demand (COD) and total suspended solids (TSS) in the RO permeate (left) and liquid-phase N and P removal efficiencies (right). Purple-shaded regions indicate compliance with ISO 30500 standards category B. Box plot elements represent the 5th/95th (whiskers), 25th/75th (box), and 50th (midline) percentiles. Bar charts (bottom) summarize median recovery efficiencies for N, P, and K from ash and water recovery from the RO permeate, with error bars showing 25th to 75th percentiles. Results are based on 10,000 Monte Carlo simulations.

The RO permeate and nutrient-rich ash products offer potential for resource recovery, but their reuse pathways differ. The PMD and SCWO HRTs can similarly recover 54.0% [35.1–72.9%] of influent water, which can be directly reused for toilet flushing. Most water losses occur in the drying system due to evaporation. Regarding nitrogen, the SCWO HRT recovers 25.6% [18.9–32.5%] of total nitrogen in the final ash product, substantially higher than the 1.28% [0.395–2.31%] recovered in the PMD HRT. Both systems lose a significant portion of nitrogen to the drying system as gaseous NH_3_ and to the RO permeate as NH_3_ or NH_4_^+^. However, the PMD system incurs additional losses during combustion, where nitrogen is released as N_2_, NO_*x*_, and N_2_O. To ensure compliance with ISO 30500 air emission requirements, especially for NH_3_, future studies should monitor the composition of off-gas streams. Phosphorus and potassium recovery in the final ash are also higher in the SCWO system, with recovery efficiencies of 91.3% [82.3–100%] for P and 88.7% [80.7–98.3%] for K, compared to 79.5% [70.8–93.6%] for P and 77.6% [67.4–89.2%] for K in the PMD system. The lower recovery observed in the PMD is primarily attributed to losses during the combustion process.^53^ Consistent with field observations, combustion is not desired if nutrients are to be recovered: the pasteurized fecal cake (often brine-coated) already provides a practical form factor for nutrient reuse. However, nutrient reuse will likely be challenging, given the limited form and quantity available.

### 3.2 Varying Solid–Liquid Separation Shifts the Balance between Thermomechanical and Thermochemical HRTs

Beyond treatment performance, solid–liquid separation is a major driver of life cycle cost and GHG emissions, and it affects thermomechanical and thermochemical HRTs differently. It was reported that solids pretreatment outlets could span 75% to 97% moisture content when a wedge-wire filter vacuum tank separator and a belt separator are used.^54^ We widened the moisture content range to 50% to 98% to gauge its influence on the costs and emissions of both HRTs. Our results show that the SCWO HRT is more sensitive to solid moisture content: raising pretreated solids’ moisture content from 80% to 95% increased per-capita cost by 6.2% and GHG emission by 66%, whereas that of an PMD HRT rose by only 0.54% and 3.4%, respectively (Figure 3A,B). Field tests showed that the steeper SCWO penalty stemmed from the extra energy needed to compress additional air to pressurize the additional water under supercritical conditions. In contrast, the PMD unit expends less energy to handle the same increment of water (Figure S1). Across the entire range of moisture contents, SCWO remains more expensive, with costs escalating sharply above 90% solids moisture. GHG emissions track a different pattern: the SCWO HRT emits less GHGs below 65% solids moisture, but above that threshold, the SCWO HRT’s emissions overtake and widen the gap because of the additional electricity consumption to compress additional air.

**Figure 3:**
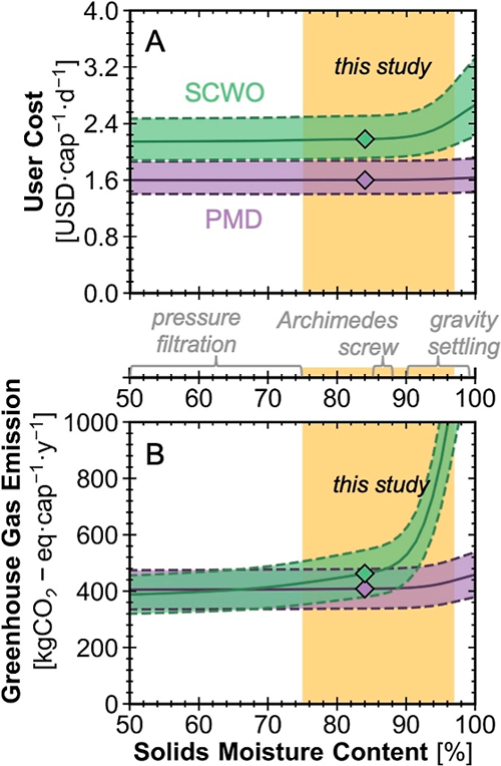
Line plot showing the relationship between (A) solids moisture content from the solids pretreatment outlet (horizontal axis) and daily per-capita cost and (B) solids moisture content and annual per-capita GHG emissions for pasteurization mechanical dewatering (PMD, purple) and supercritical water oxidation (SCWO, green) toilets. Green and purple shaded areas indicate uncertainty ranges (5th to 95th percentiles) from 10,000 Monte Carlo simulations. Diamonds indicate the median results at a solids moisture content of 84%. The yellow band denotes the range of solids moisture content used in this study. For reference, typical moisture contents from alternative solids pretreatment processes are marked by gray labeled zones between the top and bottom panels. To generate the data for this figure, the solids moisture content in the solids pretreatment outlet was varied while keeping other parameters consistent with the general scenario (Table S9).

Given the outlet moisture range in the current solids pretreatment system,^54^ the PMD configuration will generally outperform SCWO in terms of lower cost and GHG emissions. We applied alternative pretreatment options—gravity settling, Archimedes screw, and pressure filtration—using their characteristic outlet solids moisture ranges to assess how each shifts the cost and emissions. Gravity settling (90% to 99% outlet moisture)^43,55^ and an Archimedes screw (85% to 88% outlet moisture)^56^ would favor PMD, whereas deep dewatering by pressure filtration (50% to 75% outlet moisture)^43^ yields comparable or lower emissions for the SCWO HRT. These results highlight that pretreatment technologies providing greater dewatering capacity can significantly narrow or reverse the GHG emission gap between thermomechanical and thermochemical HRTs.

### 3.3 Life Cycle Costs, Greenhouse Gas Emissions, and Key Drivers

In the general scenario that represents the current design capacity (Figure 4A), baseline (excluding resource recovery) daily per-capita costs are estimated at 1.61 [1.41–1.87] and 2.18 [1.91–2.52] USD·cap^–1^·day^–1^ for the PMD and SCWO HRT, respectively. The inclusion of revenue from recovered N, P, and K fertilizers and water in the general scenario has minimal impact on costs, leading to 1.60 [1.40–1.87] and 2.18 [1.90–2.52] USD·cap^–1^·day^–1^ for PMD and SCWO HRTs, respectively: this indicates that resource recovery alone does not significantly offset costs (unpaired *t*-test *p*-value > 0.2). CAPEX accounts for over half of the costs in both systems, contributing approximately 54% of the total cost (Figure S7, top), followed by maintenance, electricity, and labor. Operating and capital expenditures drive the PMD HRT’s advantage. Annual OPEX is 2033 [1634–2601] USD·year^–1^ for the PMD configuration versus 2710 [2169–3405] USD·year^–1^ for SCWO, while CAPEX is 14,700 [13,600–15,900] USD compared with 20,700 [19,100–22,300] USD (Figure 4B). Sensitivity analysis (Figure S10) identifies the flush water volume, labor wage, and electricity unit price as the strongest cost drivers for both toilets. SCWO cost is additionally sensitive to the SCWO reactor’s capital and material replacement costs. In the general scenario, both HRTs currently cost significantly more than CRTs (estimated to cost between 0.03 and 0.26 USD·cap^–1^·day^–1^),^22,35,57^ septic systems (between 0.26 and 1.22 USD·cap^–1^·day^–1^),^58,59^ and most centralized WRRFs (typically between 0.02 and 1.59 USD·cap^–1^·day^–1^).^13,51,60^

**Figure 4:**
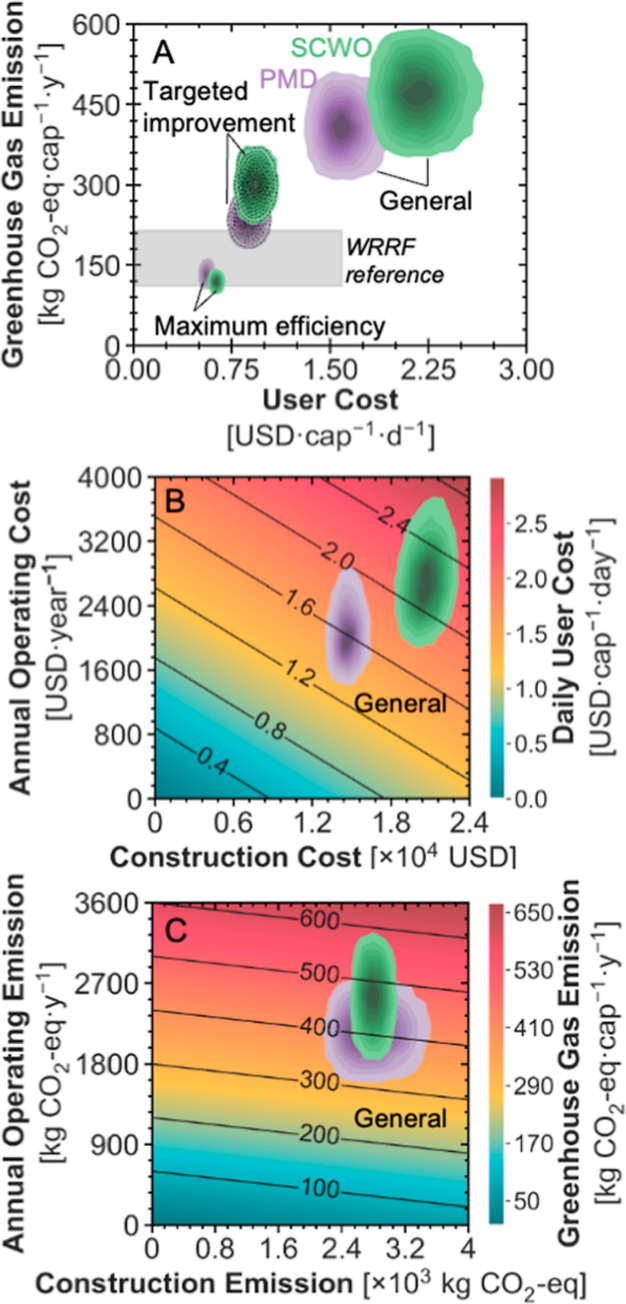
Estimates of economic and environmental outcomes for pasteurization mechanical dewatering (PMD, purple) and supercritical water oxidation (SCWO, green) toilets under different scenarios (excluding resource recovery). (A) Kernel density plot depicting the distribution of daily per-capita cost (horizontal axis) and annual per-capita GHG emissions (vertical axis) in three different scenarios: (i) general (pessimistic learning curve for scaled CAPEX projection), (ii) targeted improvement (optimistic learning curve with improved key parameters: a user count of 8, flush water use of 0.2 L per flush, a reduced SCWO reactor cost of 1200 USD, and a lower annual material replacement cost ratio of 0.08 for the SCWO reactor), and (iii) maximum efficiency (Tables S9 and S11). The gray rectangle depicts the range for the centralized water resource recovery facilities (WRRFs) across the globe to provide a general frame of reference for typical magnitudes of costs and life cycle GHG emissions of WRRFs.^51^ They do not support direct comparison with HRTs as WRRFs treat both black and gray water while HRTs treat only blackwater in this analysis. (B) Heat map illustrating estimated construction material costs (horizontal axis), operating costs (maintenance, electricity, and labor; vertical axis), and daily per-capita costs (color gradient and contour lines) for the general scenario. (C) Heat map illustrating estimated GHG emissions from construction (horizontal axis), operating GHG emissions (maintenance, electricity, and labor; vertical axis), and annual per-capita GHG emissions (color gradient and contour lines) for the general scenario. The kernel density plots are each based on results from 10,000 Monte Carlo simulations, and darker regions indicate higher density of results.

LCA results indicate that baseline GHG emissions (excluding resource recovery) are lower (unpaired *t*-test *p*-value < 0.01) for the PMD HRT (409 [339–479] kg CO_2_-eq·cap^–1^·year^–1^) than for the SCWO HRT (472 [386–557] kg CO_2_-eq·cap^–1^·year^–1^; Figure 4A). Inclusion of resource recovery—through recovery of N, P, K, and water—provides only marginal emission reductions, with life cycle emissions decreasing roughly 1% to 406 [337–477] kg CO_2_-eq·cap^–1^·year^–1^ for the PMD HRT and 466 [381–551] kg CO_2_-eq·year^–1^ for the SCWO HRT. Since construction emissions are comparable between systems (2780–2790 kg CO_2_-eq, unpaired *t*-test *p*-value > 0.5), the difference in life cycle emissions is primarily driven by operations (Figure 4C). Operations account for 2170 [1750–2600] kg CO_2_-eq·year^–1^ GHG emissions for the PMD HRT and 2550 [2040–3060] kg CO_2_-eq·year^–1^ for the SCWO HRT. Grid electricity is the primary source of operating emissions, accounting for 87% to 90% of total emissions in both toilets (Figure S9, top). Energy demand is high—PMD and SCWO HRTs consume 2.0 [1.2–3.5] and 2.3 [1.4–3.8] kWh·cap^–1^·day^–1^, respectively—significantly above the 0.19 [0.09–0.42] kWh·cap^–1^·day^–1^ typical of centralized treatment.^61^ The unit CI of electricity, therefore, dominates the sensitivity results: the correlation coefficient is 0.97 to 1.00 for both toilets (Figure S10). In the general scenario, both HRTs emit more than centralized WRRFs (111–215 kg CO_2_-eq·cap^–1^·year^–1^),^13,62,63^ septic systems (180–231 kg CO_2_-eq·cap^–1^·year^–1^),^64^ and CRTs (28.0–112.3 kg CO_2_-eq·cap^–1^·year^–1^).^35,57^

### 3.4 Targeted Improvement toward Maximum Efficiency

A short-term pathway to lower HRT costs and emissions is to adjust a handful of parameters identified as the most influential, namely, system lifetime, user count, flush water volume, and the capital and maintenance costs of the SCWO reactor (dictated by the detailed design of the SCWO reactor).

#### 3.4.1 Increase System Lifetime and User Count

An HRT’s lifetime and user count will vary with its operating context. Improper use, frequent interruptions, or neglected maintenance can shorten the system service lifetime, whereas sound engineering, informed user behavior, and routine upkeep can extend it. Similarly, user count and, thus, waste loading can potentially be increased through improved design and operation. To gauge how lifetime and user count affect daily per-capita costs and annual per-capita GHG emissions, we resimulated system performance for lifespans from 2 to 20 years and occupancies from 1 to 12 users. Starting from the general-scenario baseline of six users, we scaled component sizes (pumps, blowers, compressors, tanks, membranes, etc.), electricity demand, and maintenance requirements in proportion to the assumed user count (Table S10).

Increasing either system lifetime or user count significantly reduces costs, though with diminishing returns beyond a certain threshold. For example, extending the system lifetime from 4 to 7 years (with 6 users) reduces the median costs from 2.40 to 1.83 USD·cap^–1^·day^–1^ for the PMD HRT and from 3.17 to 2.41 USD·cap^–1^·day^–1^ for the SCWO HRT (Figure S12A,B). Similarly, increasing the number of users from 2 to 6 (with a fixed 10 year lifetime) lowers the median costs from 3.60 to 1.60 USD·cap^–1^·day^–1^ for the PMD HRT and from 5.04 to 2.11 USD·cap^–1^·day^–1^ for the SCWO HRT. However, further extending the system lifetime from 10 to 15 years (with 6 users) only marginally reduces costs from 1.60 to 1.43 USD·cap^–1^·day^–1^ for the PMD HRT and from 2.11 to 1.89 USD·cap^–1^·day^–1^ for the SCWO HRT. Similarly, increasing users from 10 to 15 (with a 10 year lifetime) has a limited effect on cost reduction, from 1.22 to 1.04 USD·cap^–1^·day^–1^ for the PMD HRT and from 1.53 to 1.24 USD·cap^–1^·day^–1^ for the SCWO HRT. The upper thresholds for achieving a significant cost reduction for increasing lifetime or user number intersect at approximately 8.5 users per toilet and 7 years of system lifetime. This indicates that in the general scenario (6 users and a 10 year lifetime), increasing user count delivers greater economic benefit than further extending the lifetime.

Increasing lifetime and user number also reduces annual per-capita emissions (Figure S12C,D), but the threshold for meaningful reduction is reached earlier at around 4.5 users and a 5 year system lifetime. This indicates that emissions are already near minimum levels (without optimizing other parameters) in the general scenario. Further emission reductions would require addressing grid electricity CI.

#### 3.4.2 Reduce Flush Water

To isolate the effects of the OPEX of flush water volume, the HRT size (i.e., CAPEX) was held constant across flush volume ranges in this section. A positive linear relationship exists between flush water volume and daily per-capita costs for both HRTs (Figure S14). Current ultralow-flush designs can achieve as low as 0.2 L per flush. Reducing flush volume from the current U.S. federal standard of 6 L per flush^65^ to 0.2 L per flush lowers costs from 1.95 [1.67 to 2.28] to 1.44 [1.30–1.59] USD·cap^–1^·day^–1^ for the PMD HRT and from 2.46 [2.11–2.85] to 1.94 [1.72–2.19] USD·cap^–1^·day^–1^ for the SCWO HRT. The observed cost reductions mainly arise from lower OPEX for pumping, heating, and pressurization. However, field tests indicate users prefer a higher flush volume at 0.8 L per flush, likely reflecting both pre-existing habits and the unsatisfactory cleaning performance experienced at lower flush volumes.^34^ Increasing the flush volume to 0.8 L per flush raises costs slightly to 1.49 [1.34–1.65] and 2.00 [1.77–2.25] USD·cap^–1^·day^–1^ for the PMD and SCWO HRT, respectively, representing a 3% cost increase as compared to 0.2 L per flush. While the trade-off between costs and flush volume ultimately is up to the user’s choice, field tests also show that an ultralow flush below the 0.8 L per flush threshold will likely incur additional costs because auxiliary technologies and additional conveyance are required—a condition outside the scope of the current analysis.

#### 3.4.3 Lower the Cost of the Supercritical Water Oxidation Reactor

In the SCWO HRT, the SCWO unit is the largest cost contributor (36%, Figure S7) due to the high capital costs and current design features that result in frequent part replacements. Reducing the baseline purchase cost of the SCWO reactor from 3483 to 1066 USD (assuming optimistic learning curve; Section S3) through increased efficiency in design and manufacturing reduces the cost from 2.10 to 1.84 USD·cap^–1^·day^–1^ (annual material replacement cost ratio fixed at 0.2; Figure S15). Likewise, reducing the annual material replacement cost ratio from 0.2 to 0.05 (SCWO system purchase cost fixed at 3000 USD) could reduce the cost from 2.06 to 1.85 USD·cap^–1^·day^–1^. Opportunities to reduce CAPEX and OPEX of the SCWO reactor include adopting high-pressure actuators, refining flow paths and operating strategies, and scaling manufacturing of capital-intensive components, but these approaches require additional development and testing.^66^

#### 3.4.4 Short-Term Targeted Improvement and Long-Term Potential

Applying the above improvements, plus a more optimistic learning-curve assumption (Table S4), reduces daily costs by 45% for PMD (0.879 [0.766–1.00] USD·cap^–1^·day^–1^) and 57% for SCWO (0.931 [0.819–1.06] USD·cap^–1^·day^–1^), placing both above the wastewater tariffs charged in 87% of utilities worldwide (Figure 4A). Corresponding emissions fall by 43% for PMD to 235 [197–273] kg CO_2_-eq·cap^–1^·year^–1^ and by 36% for SCWO to 301 [250–353] kg CO_2_-eq·cap^–1^·year^–1^ yet still exceed centralized tariffs.

Current energy data in the model are derived from pilot-scale NSS systems with underloaded pumps and compressors and modest resource efficiency. Continued research and development is expected to advance HRTs toward maximum resource efficiency over the longer term (Table S11). In that scenario, projected costs drop to 0.556 [0.515–0.601] and 0.633 [0.589–0.680] USD·cap^–1^·day^–1^, respectively, for the PMD and SCWO HRT—a 65% and 71% cost reduction from the general case—placing them above the wastewater tariffs charged in 75% and 80% of utilities worldwide (Figure 4A).^51^ The annual per-capita GHG emissions fall to 134 [114–153] and 119 [102–135] kg CO_2_-eq·cap^–1^·year^–1^ for the PMD and SCWO HRTs, respectively, representing a 67% and 75% emission reduction and placing both systems at the lower end of the centralized reference range. Notably, SCWO would then emit less GHGs than PMD—an inversion driven by the higher combustion-related direct emissions inherent to the PMD configuration.

### 3.5 Deployment Potential Varies Significantly by Context

When location-specific values for 77 countries (Figure S16) are applied to the general scenario, both HRTs display a wide performance range, largely influenced by the local labor wage, unit CI of grid electricity, price level ratio, and household size (Figure 5A). For the PMD configuration, the daily per-capita cost ranges widely from 0.477 to 8.64 USD·cap^–1^·day^–1^, with a left-skewed median of 1.54 USD·cap^–1^·day^–1^. For the SCWO configuration, the daily per-capita cost follows a similar left-skewed distribution with the lowest, highest, and median values of 0.621, 9.13, and 1.94 USD·cap^–1^·day^–1^, respectively. Sensitivity analysis shows that a higher price level ratio and labor wage are positively correlated with costs (Figure S17). Because countries with high price level ratios typically also pay higher wages, these two factors explain most high-cost outliers (Figure S18). The annual per-capita GHG emissions have an even distribution. For the PMD configuration, annual per-capita GHG emissions range from 146 to 624 kg CO_2_-eq·cap^–1^·year^–1^, with a median of 391 kg CO_2_-eq·cap^–1^·year^–1^. The SCWO configuration spans a slightly wider interval—136 to 717 kg CO_2_-eq·cap^–1^·year^–1^—and centers at a median of 439 kg CO_2_-eq·cap^–1^·year^–1^. Emission differences between countries are primarily driven by grid-electricity CI (positive correlation) and household size (negative correlation). In the general scenario, only five countries (Albania, Georgia, Kenya, Kyrgyzstan, Uganda) for PMD and three countries (Albania, Kyrgyzstan, Uganda) for SCWO fall within the global range of centralized WRRF tariffs (Table S12; note that the HRT costs still exceed the tariffs charged by utilities in those countries).^51^ These countries share four traits: low labor wage (<$2.5 h^–1^), low grid electricity CI (<0.22 kg CO_2_-eq·kWh^–1^), low local price level ratio (<0.41), and relatively large household size (>3.3 people). In contrast, countries with high labor wage and price level ratio with small households, such as Switzerland, Denmark, and Norway, exhibit the highest per-capita cost; countries with high grid electricity CI, such as Israel, Belarus, and Botswana, exhibit the highest per-capita emissions.

**Figure 5:**
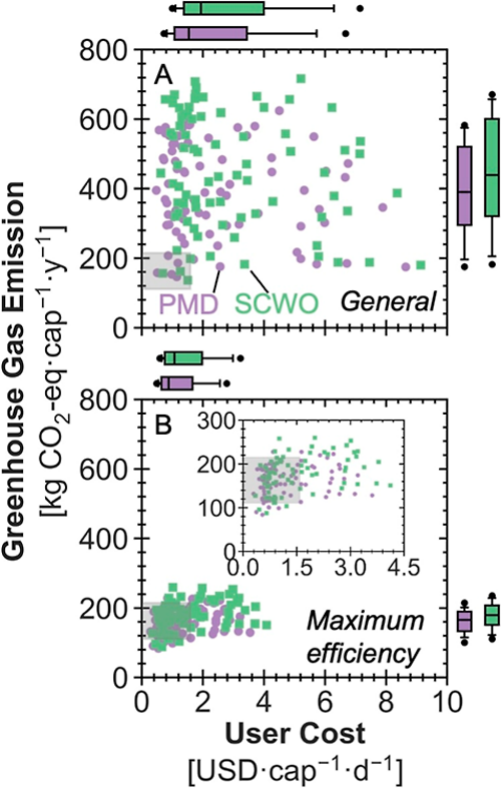
Estimates of economic and environmental outcomes for deploying pasteurization mechanical dewatering (PMD, purple) and supercritical water oxidation (SCWO, green) toilets in 77 countries in (A) the general and (B) the maximum efficiency scenario (the upper-right inset shows a magnified view). Scenario assumptions match those in Figure 4A, whereas location-specific parameters differ according to Lohman et al.^42^ Each scatter point represents the median of 5000 Monte Carlo iterations, plotted as daily per-capita cost on the horizontal axis and annual per-capita GHG emissions on the vertical axis. For the 77-country distribution, the box indicates the 25th, median, and 75th percentiles; whiskers extend to the 10th and 90th percentiles; and filled circles mark the 5th and 95th percentiles. The gray rectangle depicts the range for the centralized water resource recovery facilities (WRRFs) across the globe to provide a general frame of reference for typical magnitudes of costs and life cycle GHG emissions of WRRFs.^51^ They do not support direct comparison with HRTs as WRRFs treat both black and gray water, while HRTs treat only blackwater in this analysis.

Operating both HRTs at a maximum resource efficiency substantially decreases the cost and emissions (Figure 5B). Daily per-capita costs still display a left-skewed distribution, with the lowest, highest, and median values being 0.290, 3.54, and 0.879 USD·cap^–1^·day^–1^ for PMD and 0.348, 4.08, and 1.07 for USD·cap^–1^·day^–1^ for SCWO, respectively. Cost sensitivity also shifts (Figure S17): price level ratio becomes more influential, whereas labor wage becomes less influential as total labor hours decline under the maximum efficiency scenario. The GHG emissions’ lowest, highest, and median values are 83.8, 229, and 165 kg CO_2_-eq·cap^–1^·year^–1^ for PMD and 88.1, 260, and 179 kg CO_2_-eq·cap^–1^·year^–1^ for SCWO, respectively. Unit CI of grid electricity is still the dominant emissions driver, but its impact lessens because energy demand is lower in the maximum efficiency scenario. In this scenario, 53 countries for PMD and 44 countries for SCWO (largely emerging economies) now fall into the global centralized reference range (Table S13). Yet, when compared against utility-specific tariffs for WRRFs, only four countries show median HRT costs below existing charges (Table S14).

### 3.6 Advancing the HRT’s Research, Development, and Deployment

This study presents the first assessment of two end-members of HRTs (from low to high operating pressure and temperature) in terms of treatment performance, economic and environmental sustainability, and long-term potential based on current progress. Meeting ISO 30500 standards depends on the reliability of the solid pretreatment units (vacuum solid separator and belt separator) and the liquid treatment units (ultrafiltration and reverse osmosis) for both thermomechanical and thermochemical HRTs. Routine mechanical maintenance, membrane upkeep, and periodic sampling must therefore be embedded in field practice. Additionally, air quality compliance remains unverified; monitoring, particularly for ammonia, should be incorporated to satisfy the ISO 30500 air emissions requirements.

Thermochemical HRTs currently cost more than thermomechanical alternatives, yet their relative GHG emission performance shifts with context. Efficient solid–liquid separation lowers costs and emissions for both designs; however, weak separation penalizes thermochemical systems more severely because the additional water requires additional compressed air. With the solids pretreatment system’s variable outlet moisture (75%–97%), thermomechanical HRTs usually emit more GHGs than thermochemical HRTs owing to higher energy demand. Two exceptions emerge from the 77-country screening: exceptionally low-carbon grids, such as Albania’s 0.06 kg CO_2_-eq·kWh^–1^, where combustion emissions from the thermomechanical HRT dominate, and large-household settings (e.g., Saudi Arabia with 5.6 persons·household^–1^; Table S13) under the maximum efficiency scenario, where thermochemical HRTs recover more energy from organics and offset their energy demand. Although heat recovery from solids is often included in thermomechanical HRTs, field tests of the PMD HRT showed that the energy content of household-scale solids did not justify the additional costs for a dedicated combustor. As energy efficiency improves or grid electricity decarbonizes, the emissions gap between the two HRT types may narrow or even reverse.

Our analysis highlights cost and emission reduction pathways. CAPEX accounts for 54% of the total costs for both systems, indicating that efficient manufacturing at scale and adopting better system designs will be key to reducing costs. Additionally, lowering the manufacturing and maintenance costs of the SCWO reactor should be prioritized for the SCWO HRT. Additional identified pathways for minor improvements include designing systems with lifespans exceeding 7 years and/or user capacities above 8.5 and optimizing flush water volume. Improving the energy efficiency of components, such as pumps and compressors, will substantially reduce the cost and GHG emissions. Deployment context can amplify these gains: locations with low price level ratios, low labor wages, and large households can further reduce costs. GHG emissions under the general scenario are 386 and 441 kg CO_2_-eq·cap^–1^·year^–1^ for PMD and SCWO HRTs, respectively, both higher than the centralized reference range.^72–74^ Three strategies offer the greatest mitigation: designing systems with lifespans of at least 5 years and/or user capacities greater than 4.5, deploying in regions with low-carbon electricity grids, and continuously improving energy efficiency. Where electricity is already low-carbon or efficiency is maximized, experimental emphasis should shift to abating CH_4_ and N_2_O emissions from the pasteurized feces cake during combustion or any other disposal methods used if combustion is not used.

Although nonbiological HRTs currently entail higher per-capita costs than biological CRTs and centralized WRRFs and lack the footprint economies of scale typical of large facilities, they excel in specialized settings. One is where space constraints are severe: nonbiological HRTs occupy only 0.5– to 1 m^2^ per household unit versus 4 to 6 m^2^ for biological CRTs that serve 100 users per unit^26^ or 25,000 to 1,700,000 m^2^ for city-scale WRRFs that serve the complete wastewater management needs of populations in the tens of thousands to millions.^70,71^ Such constraints are common at urban pop-up venues and emergency deployments, such as music festivals, sports fan zones, field hospitals, and refugee intake centers. These venues also demand rapid decontamination under fluctuating loads: units must arrive ready to operate, sanitize waste quickly, sit idle for long intervals, and then resume full capacity without process upset. Biological systems struggle to meet these criteria because they require a relatively consistent influent and extended start-up times. In addition, nonbiological HRTs perform well where biology or sewers are impractical: in closed or extreme environments (e.g., Arctic regions, space stations) and in areas where trenching is constrained by topography (e.g., impermeable clay soil) and narrow space.^72–75^ In these contexts, HRTs provide reliable sanitation without the bioreactors that require carefully maintained microbial communities and without the sewer networks required by WRRFs.^67–69^

Given that this study focused on HRT technology viability and development, future studies may place greater focus on the broader social–ecological systems in which HRT systems are deployed.^45,76^ This may include expanding environmental impact categories to include (for example) eutrophication potential and ecotoxicity to provide a more comprehensive environmental assessment, as well as addressing social factors through structured planning and design processes and by explicitly including social sustainability indicators (e.g., odor control, exposure risks, user acceptance).^45,76–78^ Capturing these factors will require locality-specific assessments that reflect real deployment constraints; in particular, evaluating the accessibility of replacement components, integrating prototype-specific data (e.g., bill of materials, component energy consumption, and field performance), and conducting user experience surveys would help set priorities that are appropriate for the current development stage of each prototype.

Overall, nonbiological HRTs show promise as advanced NSS technologies for decentralized blackwater treatment and resource recovery. Although current costs and environmental impacts remain high, prioritizing improvement pathways can accelerate the learning and technological advancements of HRTs technology, improving economic and environmental sustainability over time.^79^ Integrated financial subsidies, institutional governance, and community engagement are critical to the success of sanitation systems in most contexts and especially in low-income settings. The need for sanitation systems that require compact, small-scale, rapid treatment can be met by nonbiological HRTs, and advancing their research, development, and deployment will diversify the global sanitation portfolio and have the potential to enhance circularity and resource efficiency.

## Supplementary Material



## Data Availability

All data and code used in this study are available in the QSD-Group EXPOsan repository under the “toilet” branch. This branch may be merged into the main branch in the future (https://github.com/QSD-Group/EXPOsan/tree/toilet/exposan/g2rt).

## Notes

Summary of nonbiological HRTs, supplementary solid–liquid separation analysis results, detailed process flow diagrams, influent blackwater composition, key parameters and methods for process simulation, TEA, LCA, cost and emission breakdown under different scenarios and HRT configurations, supplementary uncertainty and sensitivity analyses results, key parameters and assumptions of user scale up and operation under maximum efficiency, supplementary HRTs-targeted improvement analysis results, location-specific input data, and supplementary location-specific analysis results (PDF)
