# Supplementary material for: The Potential of Thermomechanical and Thermochemical Processes to Enable Sustainable Household Sanitation
Source: Environ Sci Technol. 2026 Jan 15;60(8):6227–38. doi: 10.1021/acs.est.5c15639 (PMC12961933; doi:10.1021/acs.est.5c15639)
Supplement: Supplementary file 1 [file es5c15639_si_001.pdf]

Supplementary Information for

The potential of thermomechanical and thermochemical processes to enable sustainable household sanitation

Zixuan Wang[1], Jianan Feng[1], Buai Shi[1], Johanna Arita Mendoza[1], Xinyi Zhang[1], Nina Trousdale[1], Roland D. Cusick[1], Shannon Yee[2], Jeremy S. Guest[1,3,*]

[1] The Grainger College of Engineering, Department of Civil and Environmental Engineering, University of Illinois Urbana-Champaign, Newmark Civil Engineering Laboratory, 205 N. Mathews Avenue, Urbana, IL 61801, USA.

[2] George W. Woodruff School of Mechanical Engineering, Georgia Institute of Technology, Atlanta, GA 30332, USA.

[3] Institute for Sustainability, Energy, and Environment, University of Illinois Urbana-Champaign, 1101 W. Peabody Drive, Urbana, IL 61801, USA.

*Corresponding author: jsguest@illinois.edu, Phone: +1 (217) 244-9247

Table of Contents:

Section S1. Portfolio of non-biological household reinvented toilets and solids pretreatment effects on energy consumption 1

Section S2. Parameter inputs for process simulation 3

Section S3. Methods and parameter inputs for TEA 10

Section S4. Method and parameter inputs for LCA 19

Section S5. Uncertainty and sensitivity analysis 22

Section S6. Improvement and maximum efficiency scenarios 24

Section S7. Location-specific analysis 31

References 39

This document contains:

46 pages

18 figures

14 tables

Section S1. Portfolio of non-biological household reinvented toilets and solids pretreatment effects on energy consumption

Table S1. Summary of non-biological household reinvented toilets.

[redacted]									
Thermo-mechanical	Firelight	evaporation	evaporation	combustion	N.A.[a]	1 to 2	N.A.	no	team communication [1]
	G2RT volume reduction	physical separation	mechanical dewatering & evaporative drying	pasteurization	90	1	75-95	yes	[2]
	Toronto	gravity-based solid/liquid separation	gravity-based solid/liquid separation	smoldering	200-400	N.A.	N.A.	no	[3,4]
	Nano membrane	gravity-based settling	archimedes screw	combustion	600	N.A.	18 to 22	yes	[5,6]
Thermo-chemical	HTClean	none	none[b]	hydrothermal carbonization	180-230	35	90-97	no[c]	team communication [1]
	G2RT micro-supercritical water oxidation	physical separation	none	supercritical water oxidation	367-450	221	75-95	yes	[7]

[a]. Data are not available.
[b]. Drying for the hydrothermal carbonization char product (hydrochar) is excluded.
[c]. Require additional steps to treat the process water.

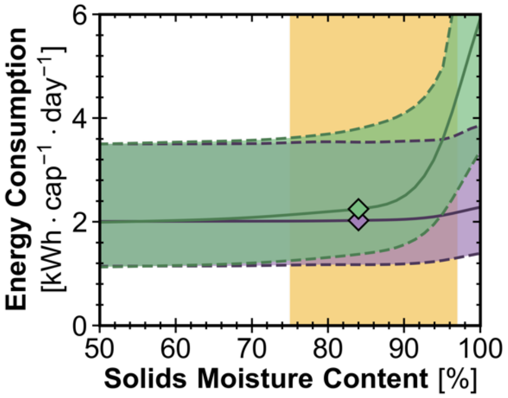

Figure S1. Line plot showing the relationship between solids moisture content (horizontal axis) from the solids pretreatment outlet and energy consumption for pasteurization mechanical dewatering (**PMD, purple**) and supercritical water oxidation (**SCWO, green**) toilets. Shaded green and purple areas indicate uncertainty ranges (5th to 95th percentiles) from 10,000 Monte Carlo simulations. Diamonds indicate the median values of baseline assumptions in the model. Vertical yellow shading indicates the reported solids moisture content from the belt separator outlet.

Section S2. Parameter inputs for process simulation

The process simulation for pasteurization mechanical dewatering (PMD) and supercritical water oxidation (SCWO) household reinvented toilets (HRTs) uses input-output relationships. For example, in the SCWO reactor, a carbon conversion efficiency of 0.945 (range: 0.9–0.99, uniform probability distribution) is applied to compute the carbon mass balance at the unit's outlet. Consequently, 94.5% of organic carbon is mineralized to CO_2, while 5.5% remain as CO and COD. Such splitting ratio parameters are applied to all waste stream components. These mass splitting parameters are listed in **Table S2.** Mass splitting parameters that are not explicitly listed can be found in the QSDsan source code (toilet branch under QSDsan and EXPOsan repositories).[8] To mitigate membrane fouling, the UF and RO units are programmed to have automatic backwash and air scrubbing components that are triggered every 0.02 [0.014-0.026] kg of influent TSS and 0.02 [0.014-0.026] kg of influent total COD, respectively (**Table S2**).

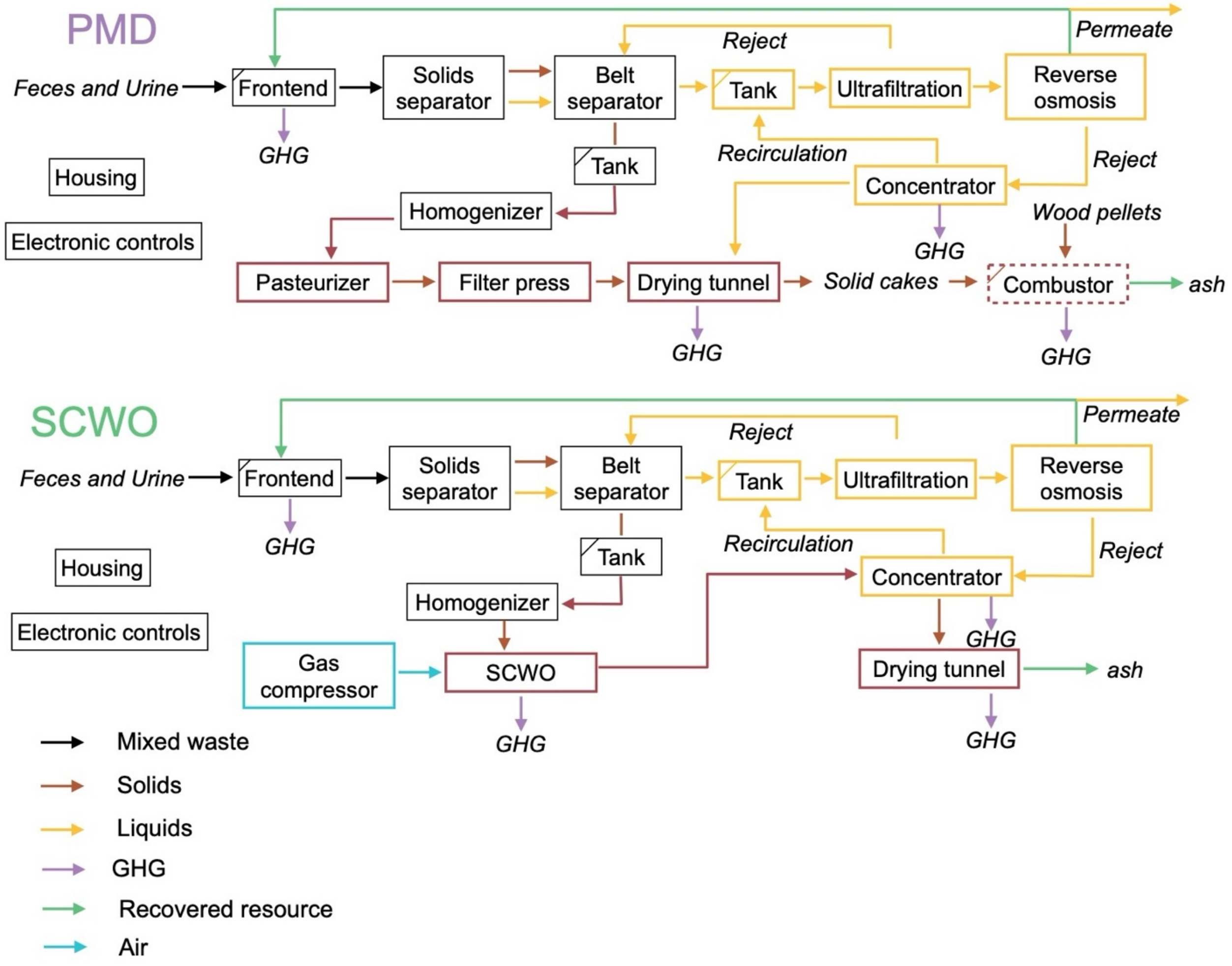

Figure S2. Illustration of process flows for the pasteurization mechanical dewatering (PMD) and supercritical water oxidation (SCWO) household reinvented toilet.

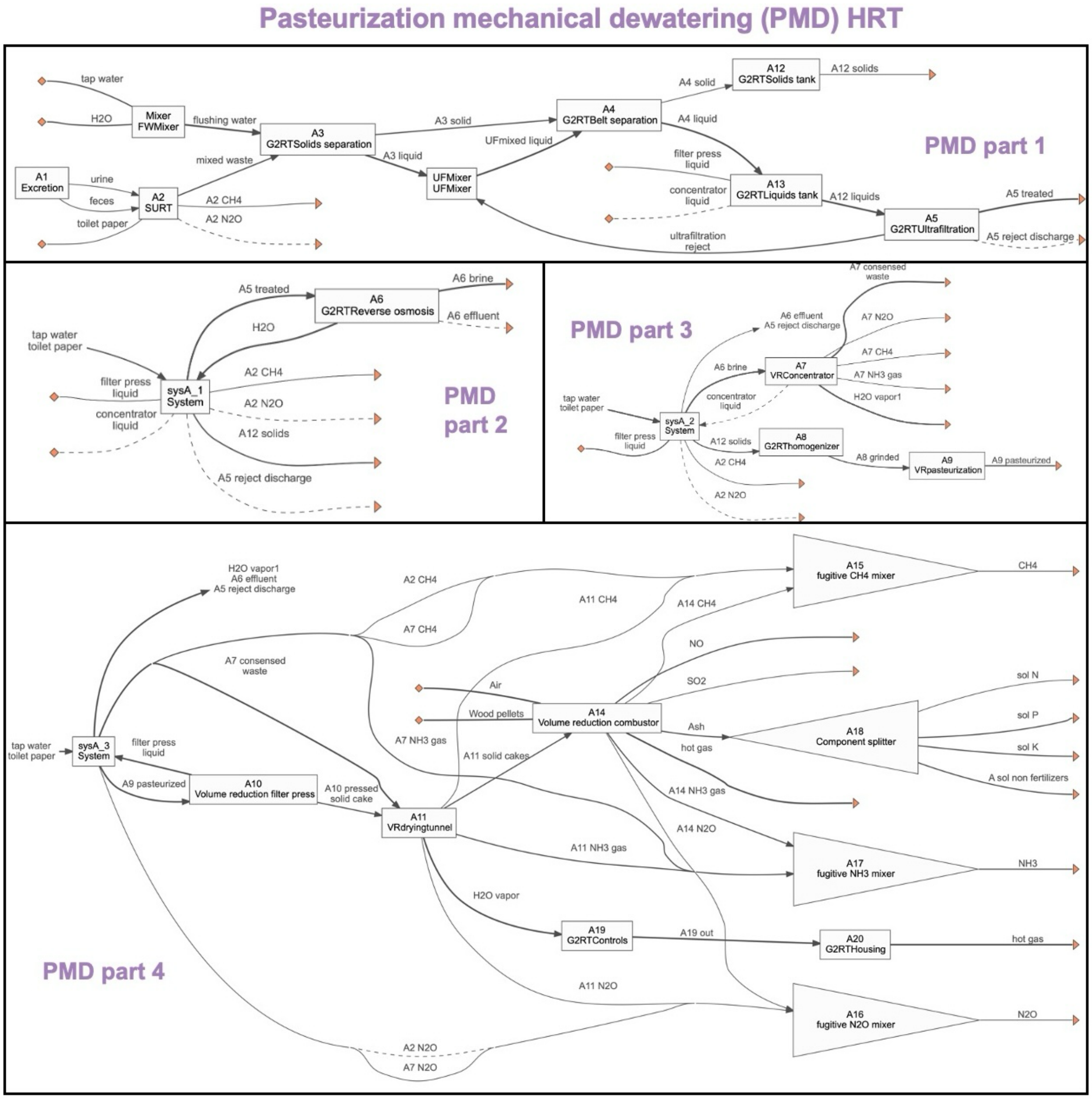

Figure S3. Detailed process flow diagram assembled in QSDsan for the pasteurization mechanical dewatering (PMD) household reinvented toilet, divided into four sequential sections (parts 1-4). The original figure files are available at https://github.com/QSD-Group/EXPOsan/tree/toilet/exposan/g2rt/readme_figures.

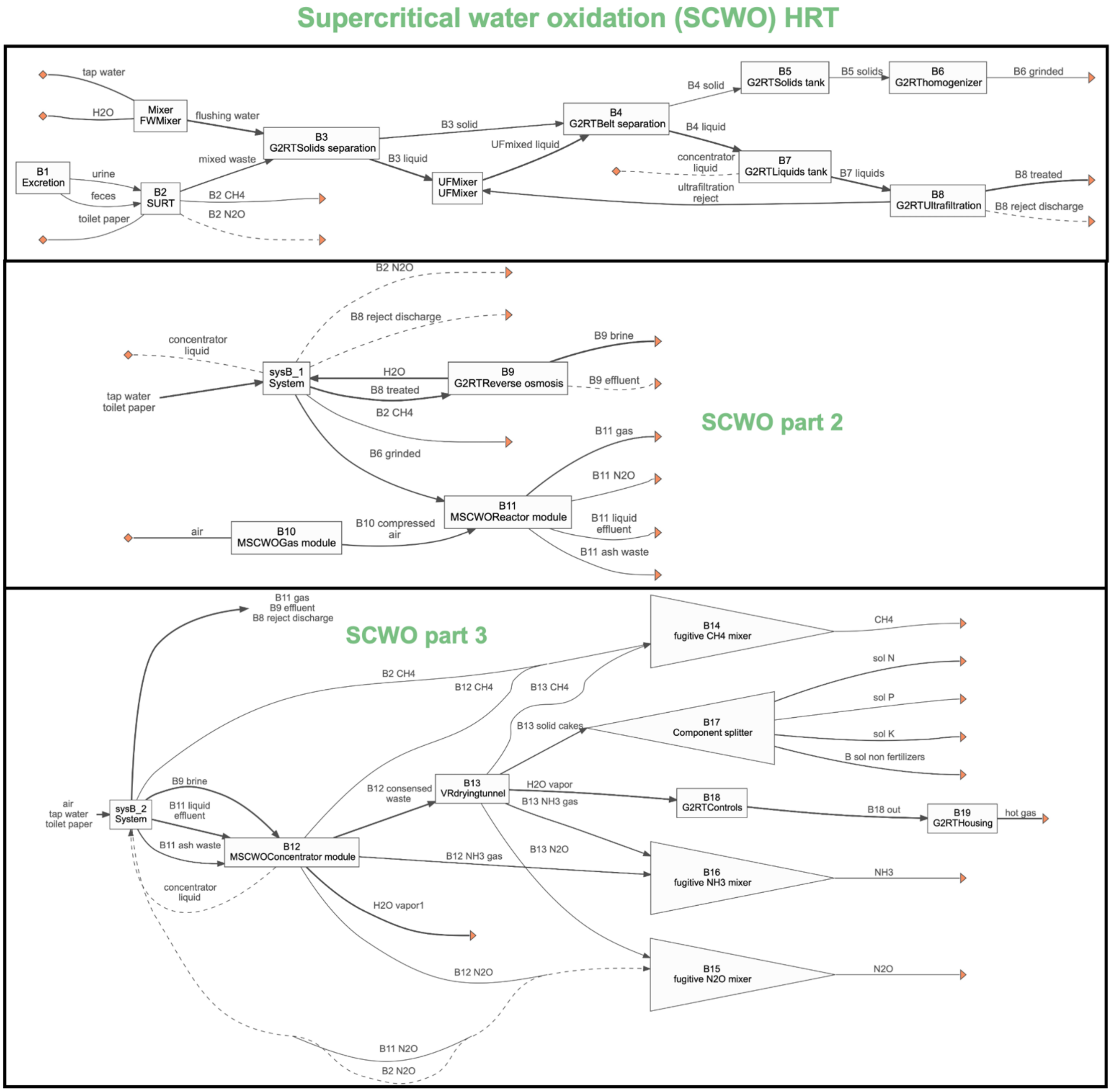

Figure S4. Detailed process flow diagram assembled in QSDsan for the supercritical water oxidation household reinvented toilet, divided into three sequential sections (parts 1-3). The original figure files are available at https://github.com/QSD-Group/EXPOsan/tree/toilet/exposan/g2rt/readme_figures.

Table S2. Characteristics of blackwater inputs.

Caloric intake [$kcal \cdot cap^{-1} \cdot d^{-1}$]	2130 (1917-2343)	uniform	[9]
Vegetal protein intake [$g \cdot cap^{-1} \cdot d^{-1}$]	40.29 (36.26-44.32)	uniform	[9]
Animal protein intake [$g \cdot cap^{-1} \cdot d^{-1}$]	12.39 (11.15-13.63)	uniform	[9]
Fraction of nitrogen content in protein	0.13 (0.13-0.19)	uniform	[10,11]
Fraction of phosphorus in vegetal protein	0.022 (0.004-0.048)	triangular	[12]
Fraction of phosphorus in animal protein	0.011 (0.002-0.032)	triangular	[12]
Potassium content in caloric intake	1.2 (1.1-1.5)	uniform	[13,14]
Fraction of excreted nitrogen in intake	1 (0.99-1.0)	uniform	[15,16]
Fraction of excreted phosphorus in intake	1 (0.99-1.0)	uniform	[15,16]
Fraction of excreted potassium in intake	0.88 (0.65-0.98)	uniform	[13,17]
Energy excretion rate (% of intake in urine and feces)	0.06 (0.02-0.10)	uniform	[18–20]
Fraction of nitrogen in urine	0.88 (0.74-0.93)	triangular	[18,19]
Fraction of phosphorus in urine	0.61 (0.33-0.75)	triangular	[18,19]
Fraction of potassium in urine	0.74 (0.53-0.93)	triangular	[18,19]
Fraction of energy in feces	0.81 (0.69-0.90)	triangular	[18,19]
Fraction of reduced inorganic nitrogen in urine	0.85 (0.75-0.90)	uniform	[18,19]
Fraction of reduced inorganic nitrogen in feces	0.20 (0.16-0.24)	uniform	[19,21]
Urine excretion [$g \cdot cap^{-1} \cdot d^{-1}$]	1400 (800-2500)	triangular	[18,19]
Feces excretion [$g \cdot cap^{-1} \cdot d^{-1}$]	250 (75-520)	triangular	[18,19]
Fraction of urine moisture content	0.95 (0.93-0.97)	triangular	[18,19]
Fraction of feces moisture content	0.85 (0.76-0.88)	triangular	[18,19]
Magnesium in urine [$g \cdot cap^{-1} \cdot d^{-1}$]	0.20 (0.12-0.21)	uniform	[18,22]
Magnesium in feces [$g \cdot cap^{-1} \cdot d^{-1}$]	0.25 (0.15-0.34)	uniform	[18]
Calcium in urine [$g \cdot cap^{-1} \cdot d^{-1}$]	0.28 (0.06-0.50)	uniform	[18]
Calcium in feces [$g \cdot cap^{-1} \cdot d^{-1}$]	1.90 (0.10-3.60)	uniform	[18]

Table S3. Parameters used for mass flow simulation throughout the units in pasteurization mechanical dewatering and supercritical water oxidation toilets.

Combustor	N_2O emission factor [g N_2O-N·g^{-1}-N]	0.0214 (0.004-0.06)	triangular	[23]	4.2
	fugitive CH_4 emission factor [g CH4·g^{-1}-feces solids]	0.00712 (0.00307-0.0307)	triangular	[23]	4.2
	NO_x emission factor [g NO_x-N·g-N^{-1}]	0.147 (0.062-0.194)	triangular	[24]	4.2
	NH_3 emission factor [g NH_3-N·g-N^{-1}]	0.0459 (0.009-0.109)	triangular	[24]	4.2
	SO_x emission factor [g SO_2-S·g-feces $solids^{-1}$]	0.00279 (0.00015-0.00062)	triangular	[25]	4.2
	feces ash content [fraction]	0.146 (0.12-0.25)	triangular	[26]	4.2
	CO_2 to CO ratio [molar ratio]	5.93 (3.35-8.1)	triangular	[26]	4.2
	biofuel to solids [g biofuel·g-feces $solids^{-1}$]	1 (0.5-2)	uniform	assumption	4.2
	wood ash content [fraction]	0.0062 (0.0049-0.0074)	triangular	[27]	1
	wood moisture content [fraction]	0.059 (0.054-0.0645)	triangular	[27]	3
	combustion N loss [fraction]	0.95 (0.9-1)	triangular	[28]	1
	combustion P loss [fraction]	0.13 (0.05-0.16)	triangular	[29]	1
	combustion K loss [fraction]	0.12 (0.02-0.26)	triangular	[29]	1
	combustion OtherSS loss [fraction]	0.85 (0.55-0.99)	uniform	assumption	3
	combustion C loss [fraction]	0.846 (0.79-0.89)	triangular	[26]	4.2
Reverse osmosis	Water recovery [fraction]	0.6 (0.33-0.9)	uniform	[30]	4.2
	TDS removal [fraction]	0.95 (0.9-0.99)	uniform	[31]	3
	NH3 removal [fraction]	0.74 (0.69-0.91)	triangular	[32,33]	1
	TSS removal [fraction]	0.999 (0.99-1)	uniform	assumption	3
Ultrafiltration	TSS removal [%]	95 (75-100)	uniform	assumption	3
	Water recovery rate [%]	90 (85-95)	uniform	[34]	3
	sCOD removal [%]	35 (30-40)	uniform	[35]	1
Belt separator	moisture content out [%]	84 (75-90)	uniform	[36]	2
	TSS removal [%]	75 (52.5-97.5)	uniform	assumption	3
Filter press	moisture content out [%]	56 (28-84)	uniform	[36]	2
	TSS removal [%]	90 (85-95)	uniform	assumption	3

Concentrator	drying CO_2 emissions [% of total C]	0.016 (0-0.032)	uniform	[37]	1.0
	drying CH_4 emissions [% of total C]	0.0003 (0-0.0006)	uniform	[37]	1.0
	drying NH_3 emissions [fraction of total N]	0.5 (0.3-0.83)	triangular	[38]	4.2
	moisture content out [%]	84 (75-90)	uniform	[39]	2
Drying tunnel	moisture content out [%]	7 (4-10)	uniform	[39]	2
Pasteurization	heat loss [%]	30 (20-40)	uniform	assumption	1
SCWO reactor	energy recovery efficiency [fraction]	0.79 (0.55-1)	uniform	[40]	1
	carbon conversion efficiency [fraction]	0.95 (0.8-0.99)	uniform	[41]	3
	ammonium nitrogen fraction [fraction]	0.84 (0.74-0.94)	uniform	[41]	3
	N_2O nitrogen fraction [fraction]	0.09 (0.03-0.15)	uniform	[42,43]	3
	P loss [fraction]	0.042 (0.01-0.1)	uniform	[44–46]	4.2
	K loss [fraction]	0.042 (0.01-0.1)	uniform	[44–46]	4.2
Solids separator	solids separator TSS removal [%]	65 (45.5-84.5)	uniform	assumption	3.0

[a.] The criterion code is explained in **Section S5**.

Section S3. Methods and parameter inputs for TEA

To account for the manufacturing CAPEX at scale for the Nth unit (100,000th unit in our analysis), we use a learning curve function:

$$C_N = \left((rC_1 - L)N^b + L\right) + (1 - r)C_1 \qquad \text{(Eq. 1)}$$

Where:

- C_N is the cost of the Nth unit;
- C_1 is the first unit cost (estimated for a single unit);
- r is the fraction of the unit cost that can be scaled through increased production volume;
- L is the minimum cost limit;
- N is the number of units;
- b is the learning curve exponent.[1]

The assumption for these parameters used in this report can be found in **Table S4**. As production scales up, costs asymptotically approach a minimum limit. We assumed a minimum cost limit of 1-3% of the first unit cost (although this limit will never be reached).[48,49] The learning curve exponent, which represents the rate of cost reduction with increased production, is calculated as (Eq. 2):

$$b = \frac{\log(learning\ curve\ percentage)}{\log(2)} \qquad \text{(Eq. 2)}$$

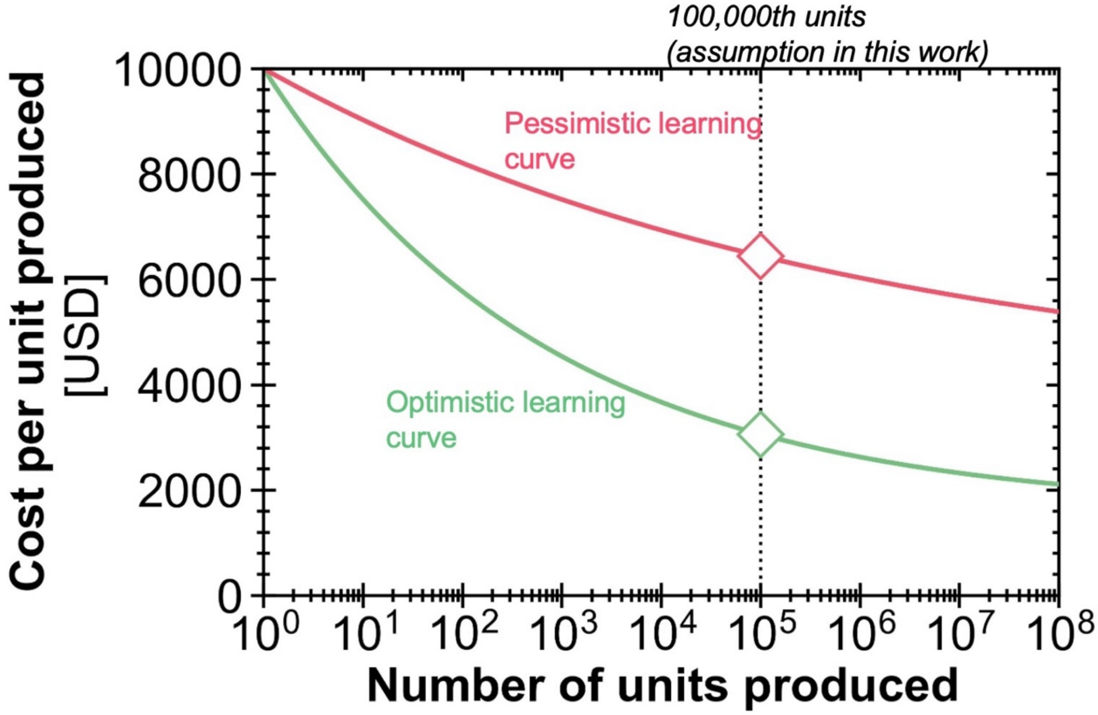

Figure S5. An example of the procedure used for scaling capital costs to reflect mass production (based on a prototype material cost of $10,000). The two curves represent optimistic (green, used in the targeted improvement and maximum efficiency scenarios) and pessimistic (red, used in the baseline scenario that represents global average case) learning curve assumptions. Diamonds indicate assumed number of units and the associated cost reduction used in this study. See **Table S4** for a complete set of parameters used to complete the economic analysis.

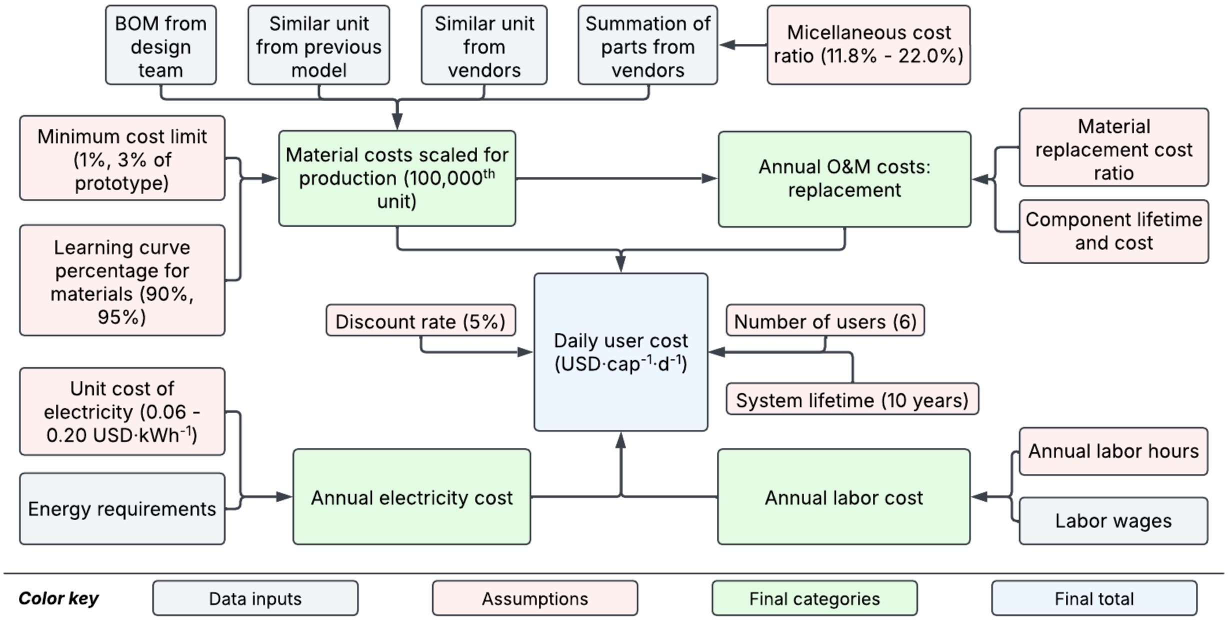

Figure S6. The process used to calculate user cost of each system. Four major cost categories were shaded in green, including upfront material cost, annual replacement cost, annual electricity cost, and annual labor cost. Sources of data inputs shown in gray boxes included information from the design team and vendors. Several assumptions, colored pink, were based on the literature and analysis of previous systems.

Electricity consumption of the components or unit treatment processes is summarized in **Table S6**. Certain energy consumption such as heating energy for pasteurization is not explicitly defined but is calculated using Python thermodynamic library Thermosteam or CoolProp. Based on the availability of data, the average power demand is calculated using one of the three following methods (ranked in descending order of preference):

1. $Power\ demand\ (kW) = \frac{\Delta Theoretical\ thermodynamic\ energy\ (kWh)}{unit\ process\ efficiency\ (\%) \cdot treatment\ time\ (h)}$
2. $Power\ demand\ (kW) = Empirical\ energy\ consumption\ of\ the\ process\ (kWh \cdot m^{-3}\ treated\ water) \cdot Wastestream\ flow\ rate\ (m^3 \cdot h^{-1})$
3. $Power\ demand\ (kW) = \frac{power\ (kW) \cdot daily\ operational\ hours\ of\ the\ unit\ (hours \cdot day^{-1})}{24\ hours \cdot day^{-1}}$

The final electricity consumption is calculated based on power, operational hours, the efficiency of the electrical process, or the volumetric flow rate to be treated. **Table S6** is not an exhaustive list and parameters that are not explicitly listed can be found in the QSDsan source code (toilet branch under QSDsan and EXPOsan repositories).[8]

Annual maintenance labor hours for each unit in HRTs are compiled in **Table S7**. These labor hours should include the time for manual cleaning, material replacement, and preventative maintenance, etc., for 6 users.

Table S4. TEA assumptions used for discounted cash flow analysis.

Discount rate [%]	5%	-	African Development Bank
Number of units for scaled production estimates	100,000	-	assumption
Optimistic fraction of CAPEX that is learnable	0.85	-	assumption
Pessimistic fraction of CAPEX that is learnable	0.65	-	assumption
Optimistic learning curve percentage for scaling capital material cost [%]	90%	-	Yelle (1979)[50]
Pessimistic learning curve percentage for scaling capital material cost [%]	95%	-	Yelle (1979)[50]
Optimistic minimum cost limit [% of prototype cost]	1%	-	Wong (2013)[48] (Value based on analysis of Helbling's HTClean serial production estimates)
Pessimistic minimum cost limit [% of prototype cost]	3%	-	Wong (2013)[48] (Value based on analysis of Helbling's HTClean serial production estimates)
Miscellaneous cost [% of material cost before scaling]	16.9% (11.8%-22.0%)	uniform	[51]
Annual material replacement cost [% of total material cost]	4 (2-6)	uniform	assumption
Electricity cost [USD·kWh^{-1}]	$0.13 ($0.06-$0.20)	triangular	[52]
Labor wages [USD·h^{-1}]	$3.64 ($1.82-$5.46)	-	[53]
Fresh water cost [USD·ton^{-1}]	$4.01 ($3.61-$4.41)	triangular	[54]
Nitrogen fertilizer price [USD·kg-N^{-1}]	$1.51 ($1.21-$1.81)	uniform	[55]
Phosphorus fertilizer price [USD·kg-P^{-1}]	$3.98 ($3.18-$4.78)	uniform	[55]
Potassium fertilizer price [USD·kg-K^{-1}]	$1.33 ($1.06-$1.60)	uniform	[55]
Fertilizer price ratio [%]	25	-	assumption

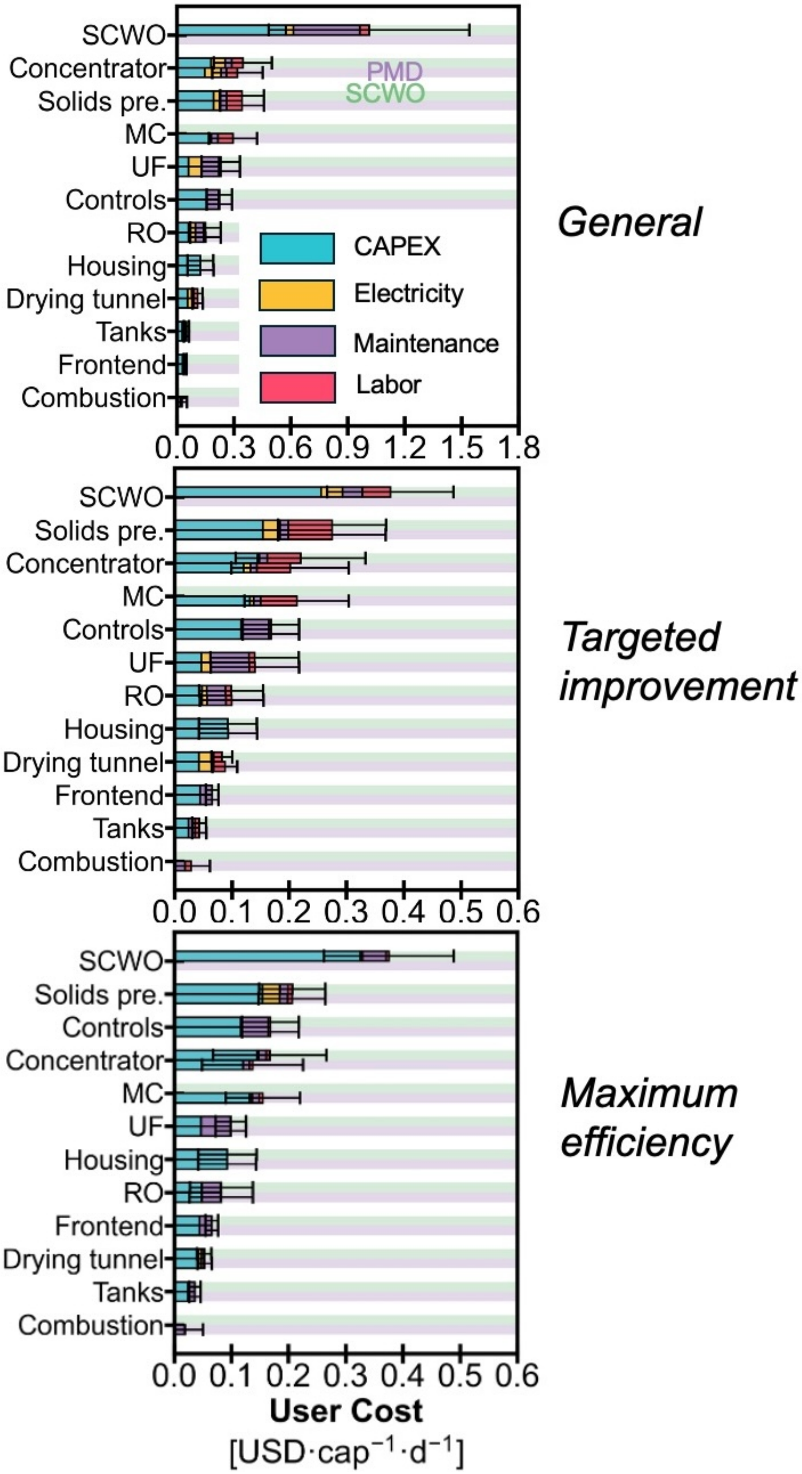

Figure S7. Breakdown of daily user costs for pasteurization mechanical dewatering (PMD, purple-shaded) and supercritical water oxidation (SCWO, green-shaded) toilets under general (pessimistic learning curve for scaled CAPEX projection), targeted improvement (optimistic learning curve with improved key parameters: a user count of 8, flush water use of 0.2 L per flush, a reduced SCWO reactor cost of 1,200 USD, and a lower annual material replacement cost ratio of 0.08 for the SCWO reactor), and maximum efficiency (**Table S10**) scenarios. All bars show median values, with error bars extending to the 5th and 95th percentiles from the uncertainty analysis. Solids pre.: solids pretreatment; SCWO: supercritical water oxidation; UF: ultrafiltration; RO: reverse osmosis.

Table S5. Selected parameters used for CAPEX estimation for each unit in pasteurization mechanical dewatering and supercritical water oxidation toilets.

Combustor	combustor cost [USD]	499 (349.3-648.7)	uniform	[56,57]	4.3
	wood pellets cost [USD·kg^{-1}]	0.759 (0.342-1.93)	uniform	[58–61]	4.2
	turbine ventilator cost [USD]	256 (179.2-332.8)	uniform	[62]	4.3
Reverse osmosis	reverse osmosis system cost [USD]	402 (70.0-1600)	triangular	[63]	4.2
	membrane cost [USD]	140 (98.2-182)	uniform	[64]	3
	piping cost [USD]	88.7 (65.1-120)	uniform	[65]	3
	back flush pump cost [USD]	89.1 (75.69-102.41)	uniform	[51]	3
	air scrubbing blower cost [USD]	200 (140-261)	uniform	[51]	4.3
Ultrafiltration	back flush pump cost [USD]	89.1 (75.69-102.41)	uniform	[51]	3
	air scrubbing blower cost [USD]	200 (140 -261)	uniform	[51]	4.3
	UF unit [USD]	860 (774-946)	triangular	[66]	3
	miscellaneous parts cost [USD]	86.3 (77.8-95.2)	uniform	[66]	3
Belt separator	belt conveyor cost [USD]	230 (184-276)	uniform	[67]	4.3
	meshed belt cost [USD]	63.9 (51.1-76.6)	uniform	[68,69]	4.3
	solid inlet chamber cost [USD]	50 (35-65)	uniform	[70]	3
	splash shield cost [USD]	89.1 (62.4-116)	uniform	[65]	3
Filter press	filter press purchase cost [USD]	1690 (1350-2030)	uniform	[71–74]	4.3
Concentrator	fan purchase cost [USD]	53.9 (37.7-70)	uniform	[75]	3
	enclosure purchase cost [USD]	52.5 (29.9-82.8)	uniform	[76]	4.3
	open concentrator vessel purchase cost [USD]	254 (203-305)	uniform	[77–80]	4.3
	heating unit purchase cost [USD]	16.6 (2.8-5.2)	uniform	[81]	4.2
	motor purchase cost [USD]	322 (42.9-648)	triangular	[82]	4.2
	phase separator cost [USD]	550 (100-1000)	uniform	assumption	2
	thermistor cost [USD]	11.8 (7.86-15.7)	uniform	[83]	1
	disc purchase cost [USD]	145 (101-188)	uniform	[84]	1
	axle purchase cost [USD]	123 (86.1-160)	uniform	[85]	3
	stainless steel cost [USD·kg^{-1}]	23.6 (5.08-42.1)	uniform	[83]	1

	pump purchase cost [USD]	379 (75.0-856)	triangular	[86]	4.2
Drying tunnel	housing cost [USD]	150 (105-195)	uniform	[87]	4.3
	duct cost [USD]	38.3 (30.7-46)	uniform	[88–90]	4.3
	ventilator cost [USD]	404 (324-485)	uniform	[91,92]	3
	conveyor cost [USD]	230 (184-276)	uniform	[93]	4.3
Pasteurization	joule heater cost [USD]	42.5 (2.65-196)	triangular	[94–99]	4.2
	thermocouple port cost [USD]	71.3 (16.4-109)	triangular	[100–104]	4.2
	temperature control [USD]	109 (76.6-142)	uniform	[105]	3
	valve cost [USD]	74.4 (16.4-195)	triangular	[106,107,107–112]	4.2
	tubing cost [USD]	268 (187-348)	uniform	[113]	3
SCWO Reactor	reactor system cost [USD]	3480 (2440-4530)	uniform	[114] & quote from Ollital laboratory	4.3
	injector cost [USD]	997 (698-1300)	uniform	[115,116]	4.3
	annual material replacement cost [fraction]	0.2 (0.1-0.3)	uniform	assumption	1
SCWO gas handling module	injection valve cost [USD]	271 (190-353)	uniform	[117]	3
	dosing valve cost [USD]	607 (363-792)	uniform	[118–120]	4.3
	vessel cost [USD]	742 (519-964)	uniform	estimated based on pressure 160 bar, diamater 50 mm, length 300 mm, rho_M = 8000 kg·m^{-3} [121]	3
	compressor cost [USD]	559 (391-727)	uniform	[122–124]	4.3
Control system	programmable logic controller cost [USD]	314 (251-376)	uniform	[51]	4.3
	IO relay module cost [USD]	48.8 (34.2-63.5)	uniform	[51]	3
	sensors cost [USD]	400 (280-520)	uniform	[51]	3
	cables cost [USD]	200 (140-260)	uniform	[51]	3
Housing	system housing cost [USD]	1800 (1260-2340)	uniform	[51]	3

Solids separator	vacuum pump cost [USD]	379 (75-856)	triangular	[125–130]	4.2
	vacuum tank cost [USD]	300 (210-390)	uniform	assumption	3
	separating filter cost [USD]	31.9 (4-122)	triangular	[131–136]	4.2
	actuator cost [USD]	127 (88.8-165)	uniform	[137]	3
	solid outlet cost [USD]	150 (105-195)	uniform	assumption	3
	liquid outlet cost [USD]	80 (56-104)	uniform	assumption	3
	inlet chamber cost [USD]	200 (140-260)	uniform	assumption	3
Homogenizer	macerator cost [USD]	252 (202-302)	uniform	[138–142]	4.3
	solid tank cost [USD]	64.3 (45-83.5)	uniform	[143]	3
Tanks	liquids tank cost [USD]	178 (78.7-255)	triangular	[144–150]	4.2
	liquids tank feed pump cost [USD]	170 (144-196)	uniform	[51]	1
	liquids tank tubing [USD]	58.2 (40.7-75.7)	uniform	[151]	3
	solids tank piping [USD]	50.9 (35.6-66.2)	uniform	[152]	3

Table S6. Selected parameters used for electricity consumption estimation for each unit in pasteurization mechanical dewatering and supercritical water oxidation toilets.

Reverse osmosis	RO energy consumption [kWh·kg^{-1} water]	0.0475 (0.029-0.066)	uniform	[66]	3
	backwash pump energy per cycle [kWh]	8e-05 (6.4e-05-9.6e-05)	uniform	[51]	3
	air scrubbing energy per cycle [kWh]	0.00417 (0.00292-0.00542)	uniform	estimated [a]	1
Ultrafiltration	ultrafiltration energy consumption [kWh·kg^{-1} water]	0.0475 (0.029-0.066)	uniform	[66]	4.2
	backwash pump energy per cycle [kWh]	8e-05 (6.4e-05-9.6e-05)	uniform	[51]	3
	air scrubbing energy per cycle [kWh]	0.00417 (0.00292-0.00542)	uniform	estimated [a]	1
Filter press	filter press energy per water [kWh·kg^{-1}]	0.00270 (0.00189 – 0.00351)	uniform	[153]	2
	Filter press energy efficiency [fraction]	0.3 [0.23-0.39]	uniform	assumption	3
Pasteurization	heat loss [%]	30 [20-40]	uniform	assumption	2
Concentrator	fan power demand [kW]	0.014 (0.0098-0.0182)	uniform	[75]	3
	pump power demand [kW]	0.215 (0.161-0.269)	uniform	[83]	3
	energy required to vaporize water [kWh·kg^{-1}]	0.15 (0.105-0.195)	uniform	estimated [b]	3
	Minimal energy required to vaporize water [kWh·kg^{-1}]	0.025 (0.02-0.03)	uniform	[154]	2
Drying tunnel	energy required to vaporize water [kWh·kg^{-1}]	0.26 (0.182-0.338)	uniform	estimated [c]	3
	Minimal energy required to vaporize water [kWh·kg^{-1}]	0.025 (0.02-0.03)	uniform	[154]	2
SCWO reactor & gas handling module	HHV feces solids [MJ·kg^{-1}]	19.9 (13.9-25.8)	uniform	[155]	3
	dosing valve power demand [kW]	0.13 (0.09-0.17)	uniform	[118–120]	3
	heating energy efficiency [fraction]	0.85 (0.595-1.0)	uniform	[156]	3
	energy recovery efficiency [fraction]	0.790 (0.553-1.0)	uniform	[40]	3
	compressor efficiency [fraction]	0.30 (0.20-0.70)	uniform	assumption	1
Control system	control system energy per cycle [kWh]	0.0103 (0.00825-0.0124)	uniform	[51]	1
	control background runtime energy per day [kWh]	0.05 (0.035-0.065)	uniform	[51]	3
	control batch cycle per day [# batches·day^{-1}]	3 (2.1-3.9)	uniform	[51]	3
Tanks	pump energy [kWh·m^{-3}]	0.083 (0.0581-0.108)	uniform	[51]	1
Homogenizer	macerator power demand [kW]	0.37 (0.259-0.481)	uniform	[157]	3
	macerator daily operation [hour·capita^{-1}·day^{-1}]	0.5 (0.35-0.65)	uniform	assumption	3
Belt separator	belt power demand [kW]	0.06 (0.042-0.078)	uniform	[93]	3

	belt daily operation [hour·capita^{-1}·day^{-1}]	0.5 (0.35-0.65)	uniform	assumption	3
Solids separator	vacuum pump power demand [kW]	0.11 (0.077-0.143)	uniform	[158]	3
	vacuum pump daily operation [hour·capita^{-1}·day^{-1}]	0.250 (0.175-0.325)	uniform	assumption	3

a. Estimated based on air flow rate: 0.6 m^3/h, pressure for membrane aeration: 0.075 MPa, blower efficiency: 0.5, air density: 1.2 kg/m^3, compressor isentropic efficiency: 0.85, working time per cycle: 10 min.[159]

b. Estimated based on inlet relative humidity: 40 %, volumetric air flow: 0.32 m^3 s^{-1}, duct cross-section: 0.18 m^2, static pressure drop: 200 Pa, blower efficiency: 0.50, disc diameter: 0.20 m, number of discs: 50, wetted surface fraction: 0.25, saturation humidity ratio: 0.0202 kg kg^{-1}, gas-phase mass transfer coefficient: 0.02 m s^{-1}.[160,161]

c. Estimated based on inlet relative humidity: 40 %, volumetric air flow: 0.32 m^3 s^{-1}, tunnel cross-section: 0.3 m^2, static pressure drop: 70 Pa, blower efficiency: 0.8, saturation humidity ratio: 0.0202 kg kg^{-1}, gas-phase mass transfer coefficient: 0.007 m s^{-1}. [160,161]

Table S7. Selected parameters used for labor hours estimation for each unit in pasteurization mechanical dewatering and supercritical water oxidation toilets.

SCWO gas handling module	gas module maintenance [hr·yr^{-1}]	6 (4.2-7.8)	uniform	assumption	3
SCWO Reactor	reactor maintenance [hr·yr^{-1}]	24 (16.8-31.2)	uniform	assumption	3
Reverse osmosis	reverse osmosis maintenance [hr·yr^{-1}]	6 (4.2-7.8)	uniform	assumption	3
Ultrafiltration	ultrafiltration maintenance [hr·yr^{-1}]	6 (4.2-7.8)	uniform	assumption	3
Combustor	combustor operation maintenance [hr·yr^{-1}]	100 (70-130)	uniform	assumption	3
Solids separator	solids separator maintenance [hr·yr^{-1}]	18 (12.6-23.4)	uniform	assumption	3
Belt separator	belt separator maintenance [hr·yr^{-1}]	20 (14-26)	uniform	assumption	3
Filter press	filter press maintenance [hr·yr^{-1}]	9 (6.3-11.7)	uniform	[162]	3
Concentrator	concentrator maintenance [hr·yr^{-1}]	36 (25.2-46.8)	uniform	assumption	3
Drying tunnel	drying tunnel maintenance [hr·yr^{-1}]	12 (8.4-15.6)	uniform	assumption	3
Homogenizer	homogenizer maintenance [hr·yr^{-1}]	12 (8.4-15.6)	uniform	assumption	3
Pasteurization	pasteurizer maintenance [hr·yr^{-1}]	40 (28-52)	uniform	assumption	3
Tanks	labor tank cleaning [hr·yr^{-1}r]	6 (4.2-7.8)	uniform	assumption	3
	Labor pump replacement [hr·yr^{-1}]	0.17 (0.14-0.19)	uniform	[51]	2
Control	control labor replacement misc repairs [hr·yr^{-1}]	3 (2.1-3.9)	uniform	[51]	3

Section S4. Method and parameter inputs for LCA

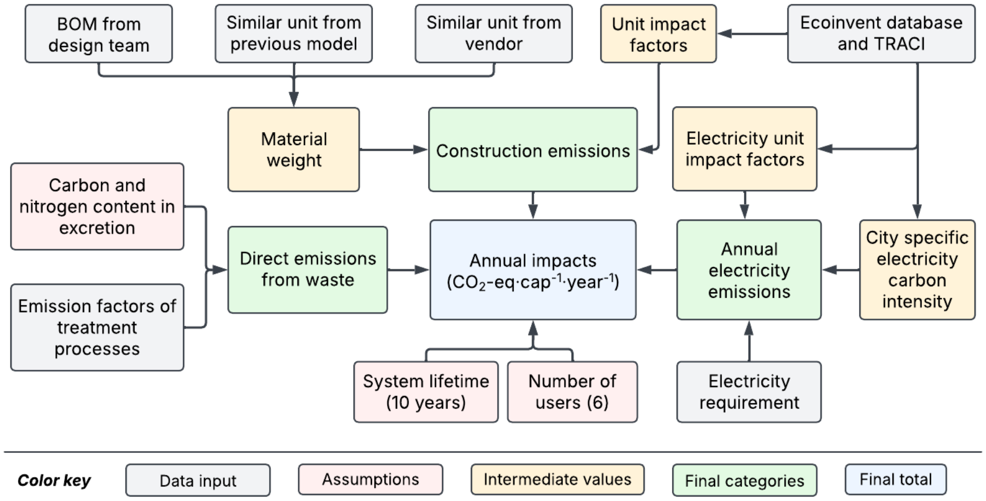

Figure S8. The process used to calculate environmental impacts of each system. Major categories of life cycle impacts include emissions from construction, treatment processes, and electricity. Unit impacts for the latter two categories were obtained using the Ecoinvent database and TRACI method and then scaled based on material and electricity use. Direct emissions from treatment were estimated from emission factors for different processes and the assumed C and N content in the excretion. End-of-life stage is excluded as end-of-life processes remain poorly understood as HRT is still in the field test stage. Assuming landfilling of the inert HRT housing material that weighs 500 to 1000 kg, the landfilling would cause GHG emissions of 0.04 to 0.09 kg CO_2-eq·cap^{-1}·year^{-1} (Ecoinvent 3.8 cutoff datasets), four orders of magnitude lower than emissions that are included in our analysis.

Table S8. LCA input parameters for electricity and direct emissions.

Electricity intensity [kg CO_2eq·kWh^{-1}]	0.69 (0.48-0.0.90)	uniform	[163]
Methane characterization factor [kg-CO_2eq·kg-CH_4 $^{-1}$]	34	-	[164]
N_2O characterization factor [kg-CO_2eq·kg-N_2O $^{-1}$]	298	-	[164]
Maximum CH_4 emission [kg-CH_4·kg-COD^{-1}]	0.25 (0.175-0.325)	triangular	[165,166]
Full degradation time	2 (1-3)	uniform	[165,166]
Log degradation	3 (2-4)	uniform	[165,166]
Nitrogen volatilization [fraction of N input]	0.005 (0.000-0.100)	uniform	[21]
MCF decay [fraction of anaerobic conversion of degraded COD]	0.10 (0.05-0.15)	triangular	[165,166]
N_2O EF decay [fraction of N emitted as N_2O]	0.0005 (0.000-0.001)	uniform	[165,166]
Methane emissions (fraction of total C, G2RT concentrator & drying tunnel)	0.0003 (0.000-0.0006)	uniform	[37]
Methane emissions (fraction of total C, combustor)	0.007 (0.000-0.031)	triangular	[23]
N_2O emissions (fraction of total N, PMD combustor)	0.147 (0.062-0.194)	triangular	[24]
N_2O emissions (fraction of total N, SCWO reactor)	0.09 (0.03-0.15)	uniform	[42,43]
Recovered water characterization factor [kg-CO_2 eq·kg-water $^{-1}$]	0.0004	triangular	[167]
Recovered N characterization factor [kg-CO_2eq·kg-N $^{-1}$]	3.50	triangular	[168]
Recovered P characterization factor [kg-CO_2eq·kg-P $^{-1}$]	3.61	triangular	[169]
Recovered K characterization factor [kg-CO_2eq·kg-K $^{-1}$]	0.88	triangular	[170]

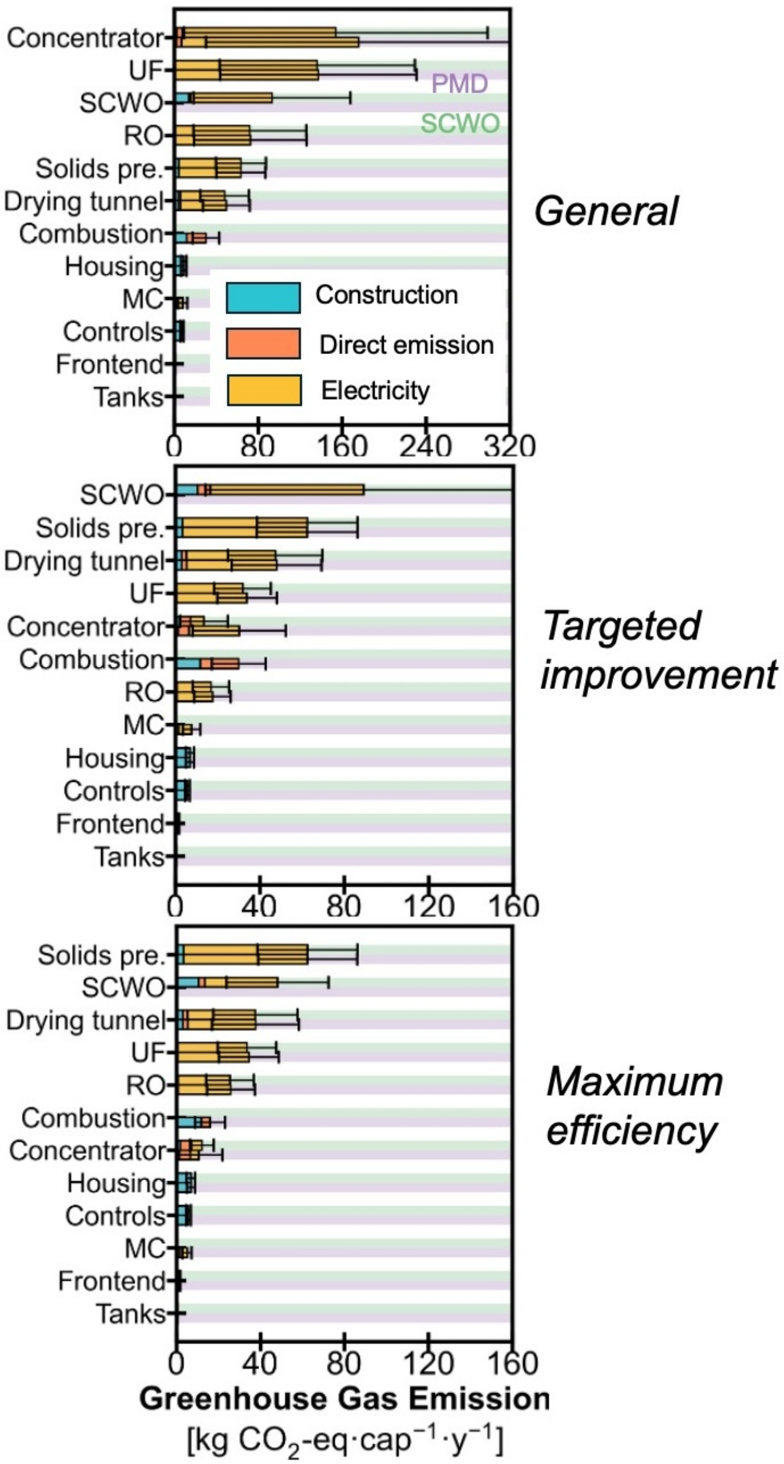

Figure S9. Breakdown of annual per capita life cycle greenhouse gas emissions for pasteurization mechanical dewatering (PMD, purple-shaded) and supercritical water oxidation (SCWO, green-shaded) toilets under general (pessimistic learning curve for scaled CAPEX projection), targeted improvement (optimistic learning curve with improved key parameters: a user count of 8, flush water use of 0.2 L per flush, a reduced SCWO reactor cost of 1,200 USD, and a lower annual material replacement cost ratio of 0.08 for the SCWO reactor), and maximum efficiency (**Table S10**) scenarios. All bars show median values, with error bars extending to the 5th and 95th percentiles from the uncertainty analysis. Solids pre.: solids pretreatment; SCWO: supercritical water oxidation; UF: ultrafiltration; RO: reverse osmosis.

Section S5. Uncertainty and sensitivity analysis

The criteria for the parameters' distribution (based on Feng et al.)[171]:

Criterion 1. For parameters where the distributions can be found in literature (e.g., wood ash content [fraction]), we used the same distributions as the literature.

Criterion 2. For parameters where only the ranges can be found (e.g., belt separator moisture content out [%]), we chose a uniform distribution with the minimum and maximum limits as the lower and upper bounds of the distribution and the average value of the minimum and maximum limits as the baseline.

Criterion 3. For parameters where only a single value can be found in the literature and is based on empirical formulae (e.g., SCWO reactor carbon conversion efficiency [fraction]), this value was assigned as the baseline and uniform distributions were set from 70% to 130% of the baseline value.

Criterion 4. For parameters with no known distribution in the literature:

Criterion 4.1. If more than 20 data points were found in the literature, we selected triangular distributions and used 5th, 50th, and 95th percentiles from the literature values as the minimum, most probable, and maximum values. If data are symmetrical and bell-shaped around a mean, normal distributions are also acceptable.

Criterion 4.2. If only 5 to 20 data points were found in the literature, we selected triangular distributions and used minimum, average, and maximum of the literature value as the minimum, most probable, and maximum values.

Criterion 4.3. If less than 5 data points can be found in the literature, we selected uniform distributions and used 70%, 100%, and 130% of the average value as lower bound, baseline, and upper bounds.

We ran Monte Carlo simulations at increments between 100 to 10,000 simulations under the general case and found the 5th and 95th percentile results from 1,000 simulations were within 1.1% of the 5th and 95th percentiles from 10,000 simulations; and those from 5,000 simulations were within 0.1% of that from 10,000 simulations. Thus, running at least 5,000 simulations would yield reproducible results. In this study, we report results from 10,000 simulations.

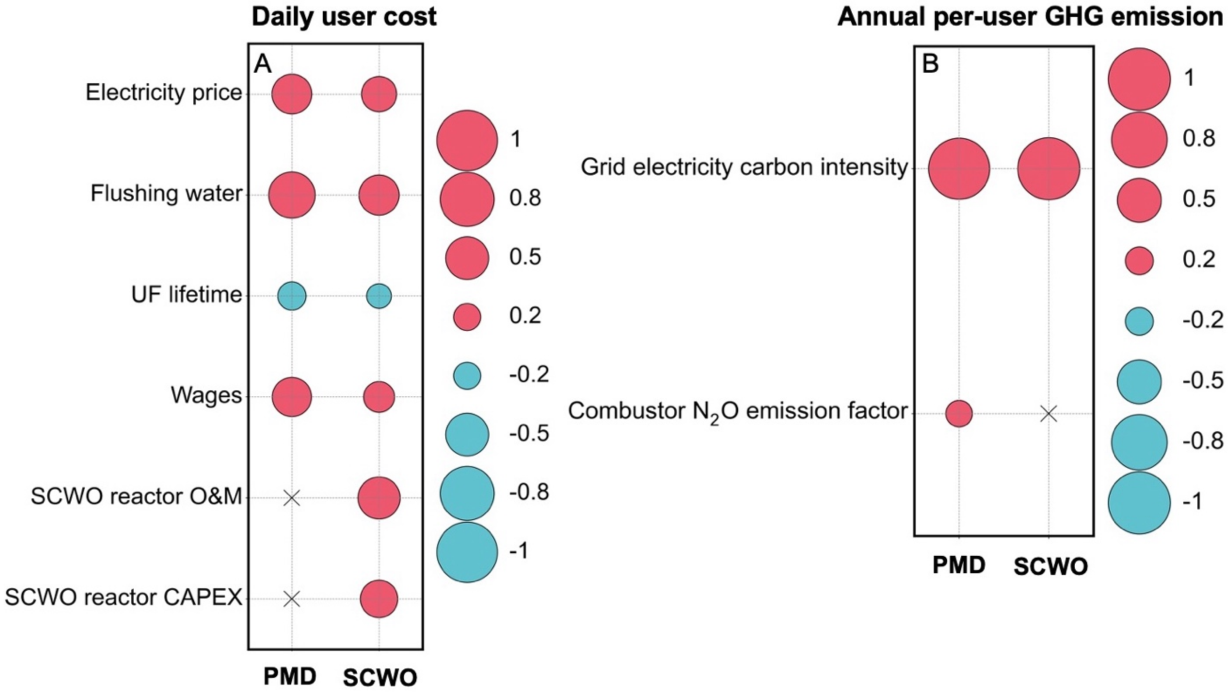

Figure S10. The Spearman's rank correlation coefficient for **(A)** daily user cost and **(B)** annual per-user GHG emissions for the PMD and SCWO toilets. The area of the bubble indicates the Spearman's correlation coefficient values, with the larger bubble indicating stronger correlation between the parameter and daily user cost or annual per-user GHG emissions. Among 445 and 429 input parameters for PMD and SCWO toilets, only the input parameters that have p-value <0.05 and the absolute value of correlation coefficient greater than 0.2 for daily user cost and 0.1 for annual per-user GHG emissions are shown. Crosses indicate the input parameter is not applicable for the toilet type. Red indicates a positive correlation, and blue indicates a negative correlation.

Section S6. Improvement and maximum efficiency scenarios
The scaling method can be found in a previous publication.[51]

Table S9. Detailed breakdown of assumption differences in general, targeted improvement, and maximum efficiency scenarios.

User number per toilet	6	increase to 8	increase to 8
CAPEX	pessimistic learning curve (**table s4**) SCWO reactor purchase cost 3,500 USD	optimistic learning curve (**table s4**) SCWO reactor purchase cost 1,200 USD	optimistic learning curve (**table s4**) SCWO reactor purchase cost 1,200 USD
Maintenance	SCWO reactor annual material replacement cost ratio 0.20	SCWO reactor annual material replacement cost ratio 0.08	SCWO reactor annual material replacement cost ratio 0.08
Electricity	**Table S6** 0.8 L per flush	**Table S6** 0.2 L per flush	**Table S11** 0.2 L per flush
Labor	**Table S7,** labor wage the same as in **Table S4**	**Table S7,** labor wage the same as in **Table S4**	Hours in **Table S7** reduce by 90%, labor wage the same as in **Table S4**

Table S10. Individual components that were scaled in cost, labor, and electricity calculations to meet the needs of increased Items scaled for increased user numbers in pasteurization mechanical dewatering (PMD) and supercritical water oxidation (SCWO) household reinvented toilets (HRTs) during simulation in each treatment unit.[51]

**Scaling method**				
	$new\ CAPEX = CAPEX * \left(\frac{new\ users}{6\ users}\right)^{0.6}$	$new\ OPEX = OPEX * \left(\frac{new\ users}{6\ users}\right)^{0.6}$	$new\ labor\ hours = labor\ hours * (\frac{new\ users}{6\ users})$	$new\ kWh = kWh * (\frac{new\ users}{6\ users})$
**Items**				
Solids separator	pumps, actuators	pumps, actuators	entire unit	pumps
Belt separator	-	belt conveyor	entire unit	belt conveyor
Tanks	tank, pumps	tank, pumps	pumps	-
Ultrafiltration	pumps, blowers, membrane	pumps, blowers	entire unit	pumps, blowers
Reverse osmosis	pumps, blowers, membrane	pumps, blowers	entire unit	pumps, blowers
Concentrator	pumps	pumps	entire unit	pumps. fans
Homogenizer	-	-	-	macerators
Pasteurization	-	-	-	heaters
Drying tunnel	-	-	entire unit	belt conveyor
Filter press	pumps	-	entire unit	motors
Combustion	combustor	combustor, wood pellets	combustor	-
Gas handling module	compressor	compressor	entire unit	compressors
SCWO reactor	-	-	entire unit	heater and compressors

To maximize efficiency, all parameters related to energy consumption, energy efficiency, and labor hours are optimized. For energy consumption, energy data from full-scale UF operation, full-scale RO operation, and solar sludge drying are used to represent the most efficient energy consumption case. All energy efficiency values are changed to 100% and heat loss values are changed to 0%. Labor hours are assumed to be reduced by 90% based on **Table S7**. Detailed changes to specific parameters are listed below.

Table S11. Parameters used to simulate pasteurization mechanical dewatering and supercritical water oxidation toilets operated under maximum efficiency.

Reverse osmosis	RO energy consumption [$kWh \cdot kg^{-1}$ water]	0.00060 (0.00042-0.00078)	uniform	[172]	3
	water recovery [fraction]	0.80 (0.56-1.0)	uniform	assumption	3
Ultrafiltration	ultrafiltration energy consumption [$kWh \cdot kg^{-1}$ water]	0.00020 (0.00014-0.00026)	uniform	[172]	3
	ultrafiltration unit lifetime [years]	8.0 (5.6-10.4)	uniform	assumption	3
Concentrator	energy required to vaporize water [$kWh \cdot kg^{-1}$]	0.025 (0.02-0.03)	uniform	[173]	2
Pasteurization	heat loss [%]	0 [0-5]	uniform	assumption	-
Drying tunnel	energy required to vaporize water [$kWh \cdot kg^{-1}$]	0.025 (0.02-0.03)	uniform	[173]	2
SCWO reactor & gas handling module	heating energy efficiency [fraction]	1.0 (0.7-1.0)	uniform	assumption	3
	energy recovery efficiency [fraction]	1.0 (0.7-1.0)	uniform	assumption	3
	compressor efficiency [fraction]	1.0 (0.7-1.0)	uniform	assumption	3
Filter press	filter press energy per water	7.60e-06 (4.10e-06-1.11e-05)	uniform	[173]	3
	filter press energy efficiency	1.0 (0.7-1.0)	uniform	assumption	3
Combustor	N_2O emission factor [g N_2O-N$\cdot$g-N^{-1}]	0.004 (0.0028-0.0052)	uniform	assumption	3
Belt separator	moisture content out [%]	70 (65-75)	uniform	assumption	2
Labor	labor maintenance hours [$hr \cdot yr^{-1}$]	Reduce by 90%	same as **Table S7**	same as **Table S7**	same as **Table S7**
Learning curve	fraction of CAPEX that is learnable	0.85	-	Assumption	-
	minimum cost limit [% of prototype cost]	1%	-	[48]	-
	percentage for scaling capital material cost [%]	90%	-	[50]	-

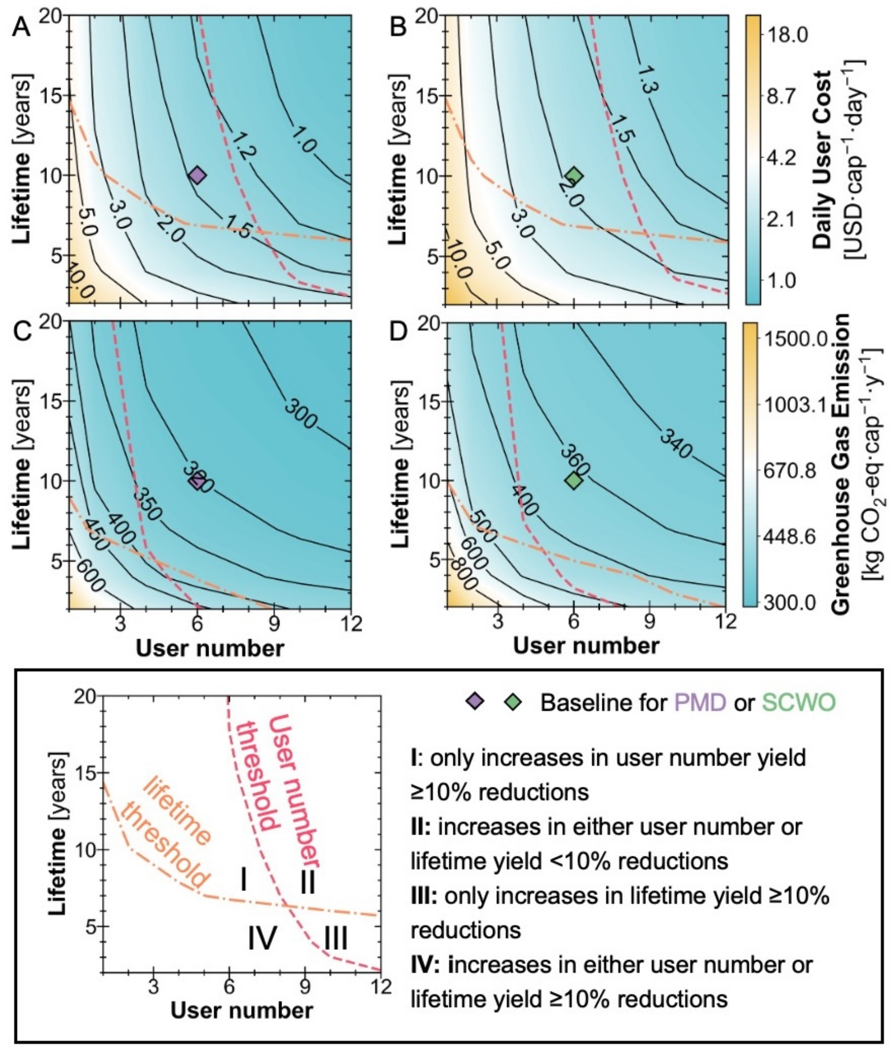

Figure S11. Estimated economic and environmental impacts of technology improvements for pasteurization mechanical dewatering (PMD) and supercritical water oxidation (SCWO) toilets.**(A–B)** Contour plots of daily user cost (color scale) as a function of user number (x-axis) and system lifetime (y-axis) for (A) PMD and (B) SCWO toilets. Red dashed and orange dash-dot lines indicate thresholds for user number and system lifetime, respectively, where each incremental increase yields a 10% reduction in user cost relative to the baseline scenario. **(C–D)** Contour plots of annual per-capita GHG emissions (color scale) as a function of user number (x-axis) and system lifetime (y-axis) for (C) PMD and (D) SCWO toilets. Thresholds reflect a 10% reduction in emissions per unit increase in user number or lifetime, relative to baseline. Diamonds mark general-scenario assumptions for PMD (purple) and SCWO (green). For all panels, only the specified input parameters are varied; all others follow baseline (general scenario) distributions. Each data point represents the **5th percentile** of 10,000 Monte Carlo simulations.

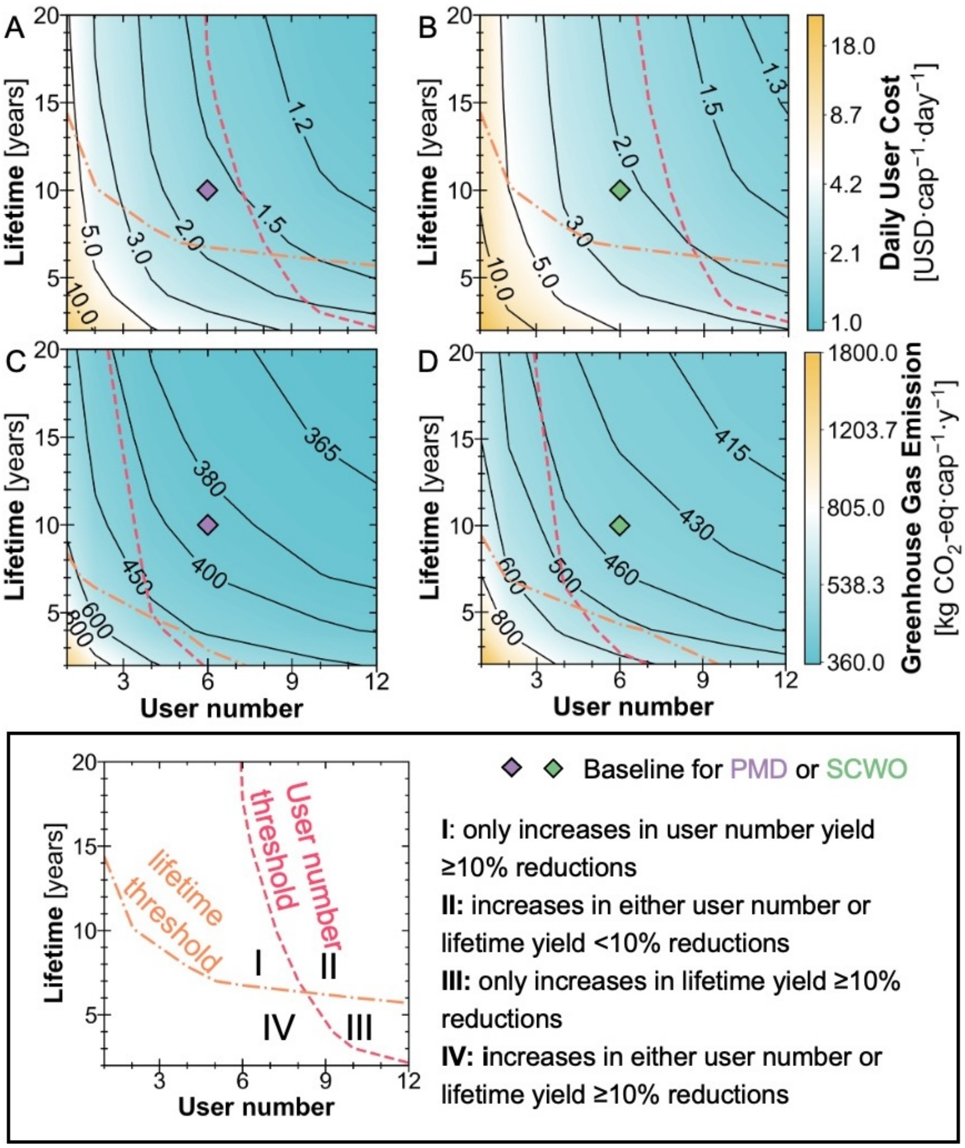

Figure S12. Estimated economic and environmental impacts of technology improvements for pasteurization mechanical dewatering (PMD) and supercritical water oxidation (SCWO) toilets. (A–B) Contour plots of daily user cost (color scale) as a function of user number (x-axis) and system lifetime (y-axis) for (A) PMD and (B) SCWO toilets. Red dashed and orange dash-dot lines indicate thresholds for user number and system lifetime, respectively, where each incremental increase yields a 10% reduction in user cost relative to the baseline scenario. (C–D) Contour plots of annual per-capita GHG emissions (color scale) as a function of user number (x-axis) and system lifetime (y-axis) for (C) PMD and (D) SCWO toilets. Thresholds reflect a 10% reduction in emissions per unit increase in user number or lifetime, relative to baseline. Regions I–IV represent different marginal benefit regimes: Region I: Only user number increases yield ≥10% reductions; Region II: Increases in either user number or lifetime yield <10% reductions; Region III: Only lifetime increases yield ≥10% reductions; Region IV: Increases in either parameter yield ≥10% reductions. Diamonds mark general-scenario assumptions for PMD (purple) and SCWO (green). For all panels, only the specified input parameters are varied; all others follow baseline (general scenario) distributions. Each data point represents the **50th percentile** of 10,000 Monte Carlo simulations.

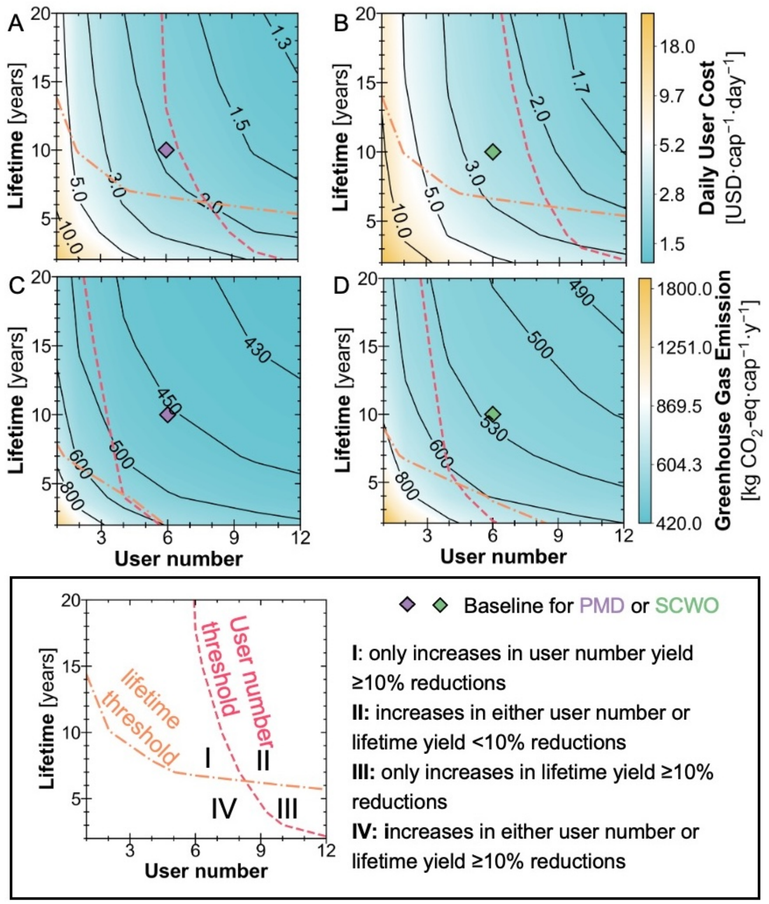

Figure S13. Estimated economic and environmental impacts of technology improvements for pasteurization mechanical dewatering (PMD) and supercritical water oxidation (SCWO) toilets.**(A–B)** Contour plots of daily user cost (color scale) as a function of user number (x-axis) and system lifetime (y-axis) for (A) PMD and (B) SCWO toilets. Red dashed and orange dash-dot lines indicate thresholds for user number and system lifetime, respectively, where each incremental increase yields a 10% reduction in user cost relative to the baseline scenario. **(C–D)** Contour plots of annual per-capita GHG emissions (color scale) as a function of user number (x-axis) and system lifetime (y-axis) for (C) PMD and (D) SCWO toilets. Thresholds reflect a 10% reduction in emissions per unit increase in user number or lifetime, relative to baseline. Diamonds mark general-scenario assumptions for PMD (purple) and SCWO (green). For all panels, only the specified input parameters are varied; all others follow baseline (general scenario) distributions. Each data point represents the **95th percentile** of 10,000 Monte Carlo simulations.

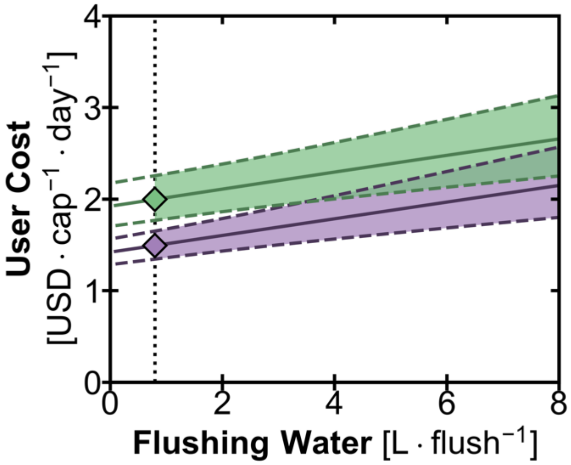

Figure S14. Line plot showing the relationship between flushing water volume and daily user cost for pasteurization mechanical dewatering (purple) and supercritical water oxidation (green) toilets. Shaded areas indicate uncertainty ranges (5th to 95th percentiles) from 10,000 Monte Carlo simulations. Only the flushing water volume is varied during simulation, while all other parameters follow baseline (general scenario) distributions. The vertical dotted line denotes the threshold of user acceptance at 0.8 L·flush^{-1}.

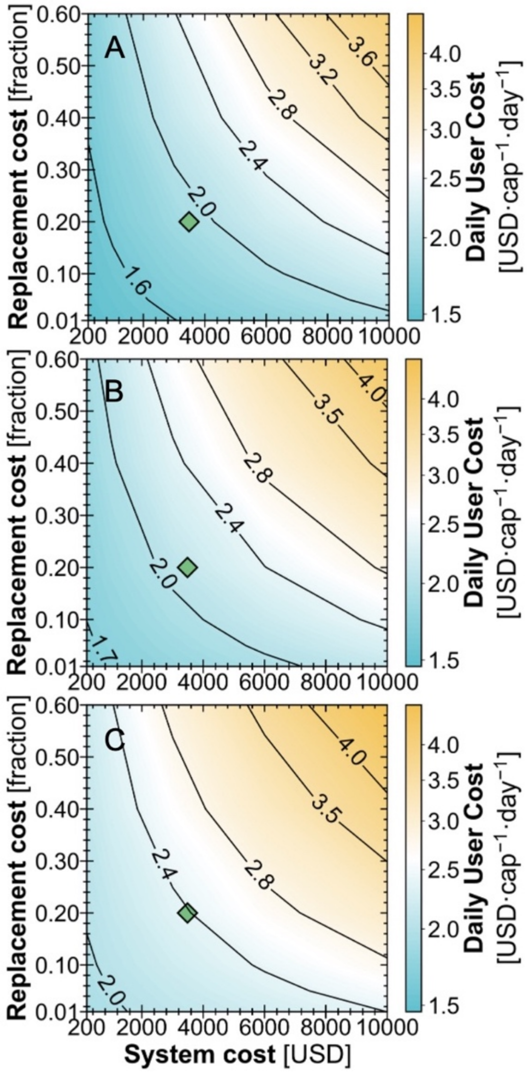

Figure S15. Contour plot of daily user cost (color scale) as a function of SCWO reactor CAPEX (x-axis) and annual material replacement cost (y-axis). Diamonds mark baseline assumption. Each data point represents the **(A)** 5th percentile, **(B)** 50th percentile, and **(C)** 95th percentile from 10,000 Monte Carlo simulations. Only SCWO reactor CAPEX and annual material replacement cost are varied during simulation, while all other parameters follow baseline (general scenario) distributions.

Section S7. Location-specific analysis

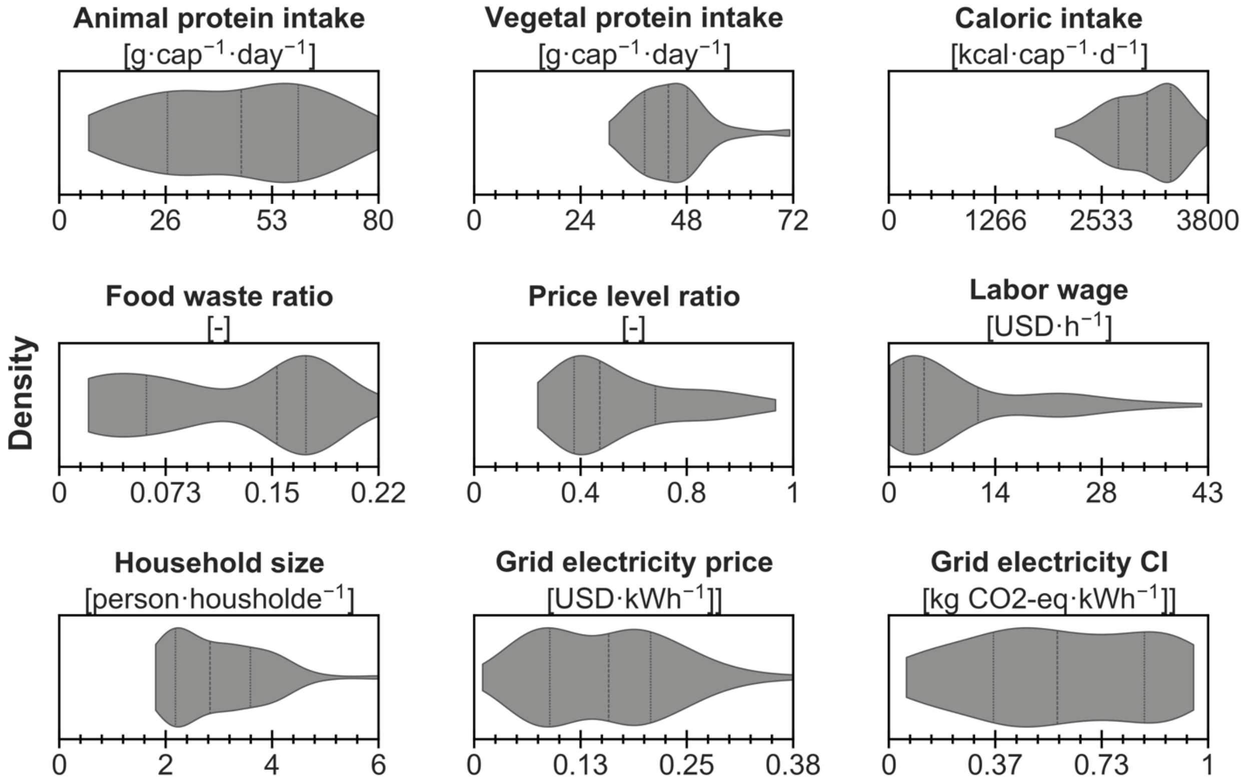

Figure S16. Distribution density of location-specific parameters (country-level data) from 77 countries. These parameters are applied to the pasteurization mechanical dewatering and supercritical water oxidation toilet models. Tabulated data are listed in a previous study.[174] Dashed lines mark the 25th, 50th, and 75th percentiles. Price level ratio: the relative cost of buying products in the country of interest compared to buying them in the United States on average.

For uncertainty analysis in each country, a 10% uniform probability distribution is applied to animal protein intake, vegetal protein intake, caloric intake, food waste ratio, and electricity price, a 20% uniform probability distribution is applied to price level ratio, a 30% uniform probability distribution is applied to electricity intensity, and a 50% uniform probability distribution is applied to labor wages.

.

Table S12. Among the 77 countries, a list of countries and household reinvented toilet configurations whose median cost and emissions fall within the centralized reference in the general scenario.

Albania	PMD	1.15	146
Georgia	PMD	0.86	207
Kenya	PMD	1.18	186
Kyrgyzstan	PMD	0.51	158
Uganda	PMD	0.98	153
Albania	SCWO	1.51	136
Kyrgyzstan	SCWO	0.69	156
Uganda	SCWO	1.26	162

Table S13. Among the 77 countries, a list of countries and household reinvented toilet configurations whose median cost and emissions fall within the centralized tariffs range in the maximum efficiency scenario.

Albania	PMD	0.71	100
Armenia		0.59	138
Bangladesh		0.54	187
Belize		1.01	99
Bolivia		0.75	168
Botswana		0.81	202
Bulgaria		1.04	172
Cambodia		0.54	120
Cameroon		0.53	118
Chile		1.04	154
China		1.14	175
Croatia		1.14	136
Czech Republic		1.46	182
Dominican Republic		0.77	189
Ecuador		0.88	114
Egypt		0.40	190
El Salvador		0.76	116
Georgia		0.58	112
Ghana		0.68	146
Greece		1.54	199
Guatemala		0.74	123
Guyana		0.86	198
Honduras		0.66	115
Hungary		1.18	154
India		0.45	165
Jordan		0.60	174
Kazakhstan		0.64	194
Kenya		0.73	101
Kyrgyzstan		0.37	91
Latvia		1.36	172
Lebanon		0.84	195
Lithuania		1.39	157
Malaysia		0.57	172
Mauritius		0.88	191
Moldova, Republic of		0.74	204

Mongolia		0.51	186
Montenegro		0.77	147
Nigeria		0.54	162
Pakistan		0.29	132
Philippines		0.61	165
Poland		1.07	207
Portugal		1.49	168
Romania		0.92	152
Russian Federation		0.91	186
Rwanda		0.59	131
Saudi Arabia		0.64	183
Slovakia		1.33	126
Sri Lanka		0.49	145
Tanzania		0.56	146
Thailand		0.74	183
Turkey		0.57	165
Uganda		0.54	84
Ukraine		0.70	156
Albania	SCWO	0.87	111
Armenia		0.72	149
Bangladesh		0.66	190
Belize		1.22	104
Bolivia		0.89	179
Bulgaria		1.26	197
Cambodia		0.65	124
Cameroon		0.65	121
Chile		1.25	166
China		1.38	188
Croatia		1.36	155
Dominican Republic		0.94	200
Ecuador		1.07	123
Egypt		0.48	195
El Salvador		0.92	121
Georgia		0.70	122
Ghana		0.83	157
Guatemala		0.90	126
Guyana		1.04	210
Honduras		0.80	119

Hungary		1.41	176
India		0.54	169
Jordan		0.73	177
Kazakhstan		0.78	208
Kenya		0.89	111
Kyrgyzstan		0.45	96
Lebanon		1.02	207
Malaysia		0.69	176
Mauritius		1.06	203
Mongolia		0.62	190
Montenegro		0.92	160
Nigeria		0.67	165
Pakistan		0.35	127
Philippines		0.74	169
Romania		1.10	172
Russian Federation		1.11	212
Rwanda		0.72	135
Saudi Arabia		0.76	182
Sri Lanka		0.60	155
Tanzania		0.68	149
Thailand		0.88	194
Turkey		0.68	172
Uganda		0.66	88
Ukraine		0.84	179

Table S14. Utilities and countries in which current WRRF tariffs exceed the estimated HRT median user cost in the maximum efficiency scenario. Tariff data are available for 37 of the 77 studied countries.[175]

Estonia	Haapsalu	PMD	1.66	1.67
	Võru	PMD	1.66	1.92
Mongolia	Darkhan	PMD	0.51	0.54
Tanzania	Iringa	PMD	0.56	0.67
United States	Atlanta Department of Watershed Management	SCWO	3.14	5.60
	Birmingham Water Works Board			3.64
	DC Water			3.32
	Golden heart utilities			3.50
	Kansas City Water Services Department			3.16
	Portland Water Bureau			3.80
	Public Works Department			3.19
	San Francisco Public Utilities Commission			4.64
	Seattle Public Utilities			4.80
	Atlanta Department of Watershed Management	PMD	2.69	5.60
	Baltimore Bureau of Water and Wastewater			2.94
	Birmingham Water Works Board			3.64
	Boston Water and Sewer Commission			2.77
	Charleston Water System			2.71
	DC Water			3.32
	Golden heart utilities			3.50
	Houston			2.80
	Kansas City Water Services Department			3.16
	Metropolitan District Commission			2.83
	Miami-Dade Water and Sewer Department			2.78
	Portland Water Bureau			3.80
	Public Works Department			3.19
	San Francisco Public Utilities Commission			4.64
	Seattle Public Utilities			4.80
	Washington Suburban Sanitary Commission			2.88

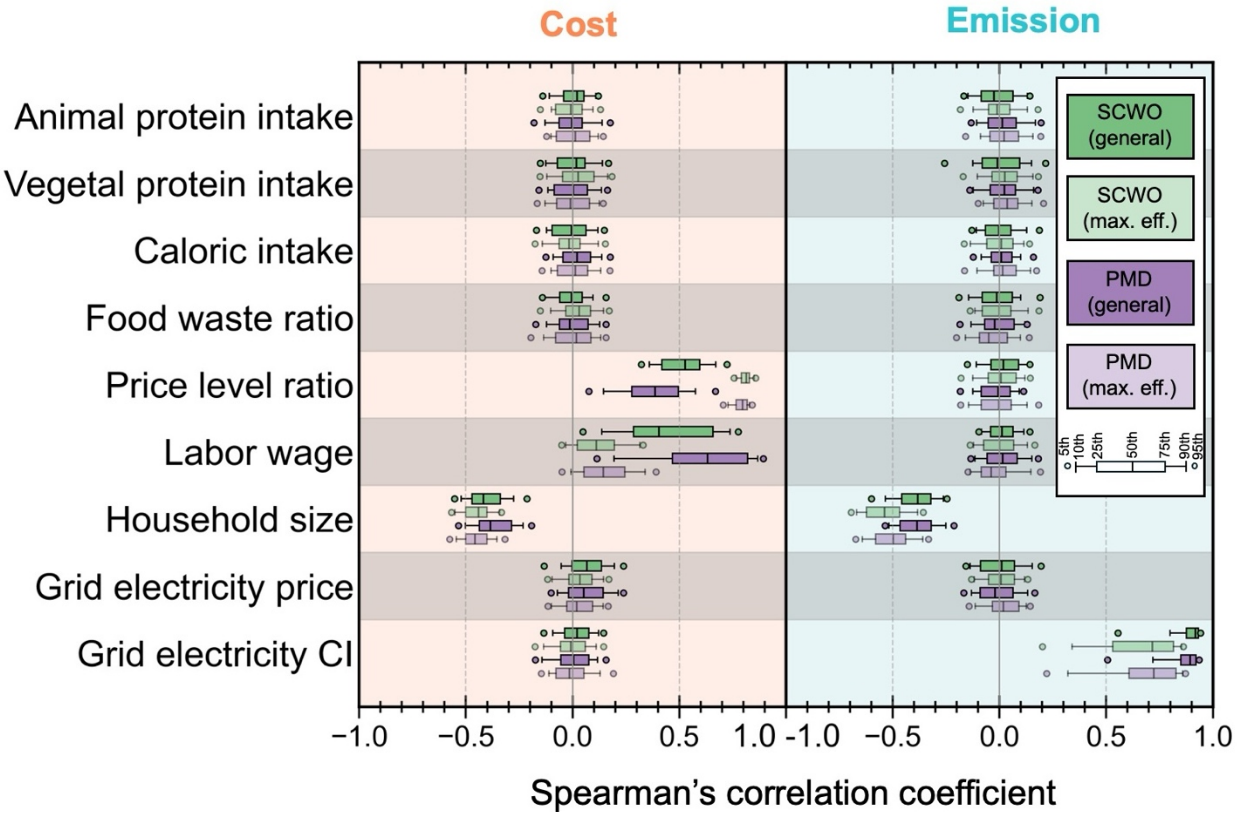

Figure S17. Spearman's rank correlation coefficients for daily user cost (left panel, orange background) and annual per-user GHG emissions (right panel, blue background) for PMD (purple) and SCWO (green) toilets in 77 countries under the general and maximum efficiency scenarios. Scenario assumptions match those in **Figure 4A**, whereas location-specific parameters differ according to Lohman et al.[176] For the 77-country distribution of the correlation coefficient, the box indicates the 25th, median, and 75th percentiles; whiskers extend to the 10th and 90th percentiles; and filled circles mark the 5th and 95th percentiles.

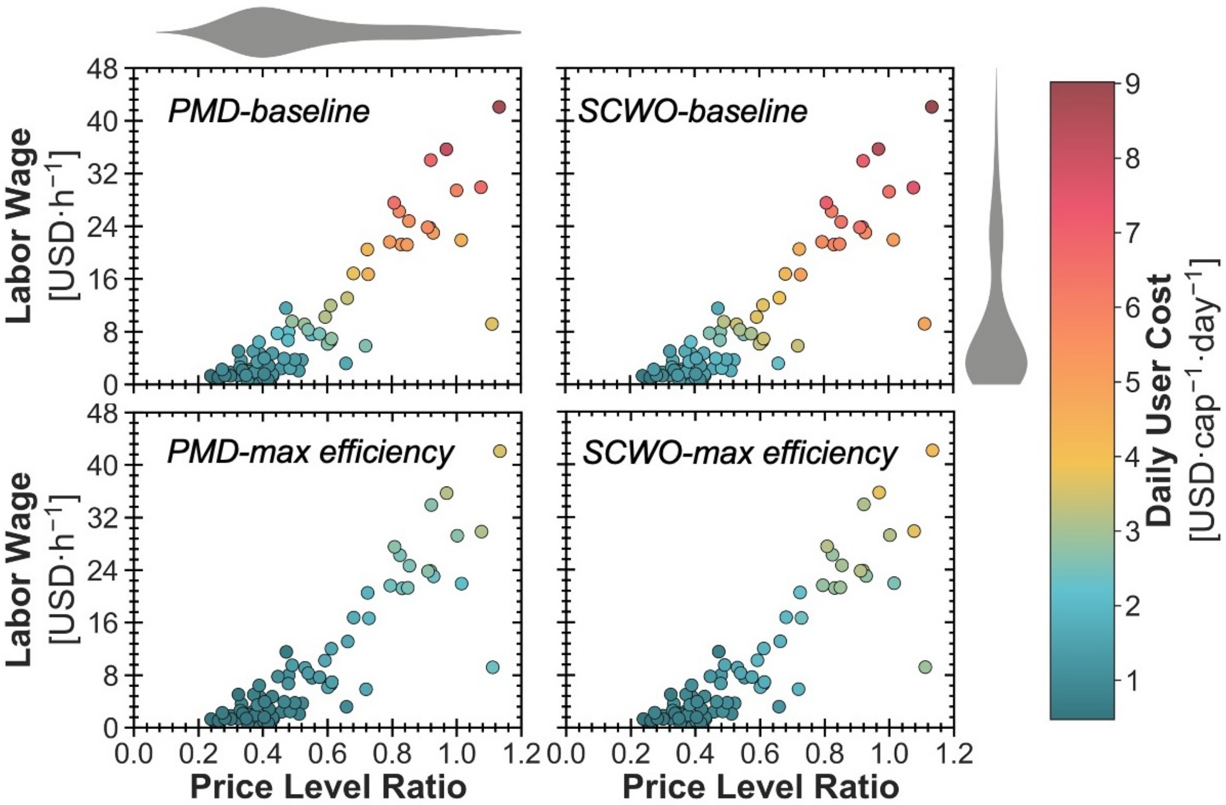

Figure S18. Estimated daily user cost for mechanical-compression (PMD) and supercritical-water-oxidation (SCWO) configurations under the general and maximum-efficiency scenarios. The horizontal axis shows each country's price-level ratio, the vertical axis its average labor wage, and the color scale encodes the median daily user cost obtained from 5,000 Monte Carlo iterations. Each scatter point represents the case of one country.

References

(1) *Janicki's reinvented Firelight Toilet*. https://www.susana.org/knowledge-hub/projects?id=298 (accessed 2025-07-01).

(2) Golly, J.; Yee, S.; Azevedo, K.; Gaylo, R.; Sherman, K.; Turner, T.; III, D. D.; Holcombe, W. D.; Holmes, J.; Noel, A.; Williams, A. N.; Richert, S.; Hawkins, B.; Miller, G.; Trotochaud, L.; Gruendl, H.; León, O. E.; Sampl, G.; Lehmann, R.; Hasler, D.; Seiler, C.; Farrér, C.; Forrer, C.; Staub, A.; Glatthard, J.; Schlauri, M.; Caduff, M.; Fischer, F.; Frasson, V.; Gemperli, A.; Rüdisüli, D.; Davey, C.; Mcadam, E.; Ravndal, K.; Mizia, J.; Willman, M. Volume Reduction Non-Sewered Single Unit Toilet System. WO2023288326A1, January 19, 2023. https://patents.google.com/patent/WO2023288326A1/en?oq=WO2023288326A1 (accessed 2025-04-15).

(3) *The Toronto Toilet - ANSI Sanitation*. https://sanitation.ansi.org/TorontoToilet (accessed 2025-07-01).

(4) Fabris, I.; Cormier, D.; Gerhard, J. I.; Bartczak, T.; Kortschot, M.; Torero, J. L.; Cheng, Y.-L. Continuous, Self-Sustaining Smouldering Destruction of Simulated Faeces. *Fuel* **2017**, *190*, 58–66. https://doi.org/10.1016/j.fuel.2016.11.014.

(5) *Nano Membrane Toilet*. https://www.cranfield.ac.uk/case-studies/nano-membrane-toilet (accessed 2025-07-01).

(6) Kolios, A.; Jiang, Y.; Somorin, T.; Sowale, A.; Anastasopoulou, A.; Anthony, E. J.; Fidalgo, B.; Parker, A.; McAdam, E.; Williams, L.; Collins, M.; Tyrrel, S. Probabilistic Performance Assessment of Complex Energy Process Systems – The Case of a Self-Sustained Sanitation System. *Energy Convers. Manag.* **2018**, *163*, 74–85. https://doi.org/10.1016/j.enconman.2018.02.046.

(7) Yee, S.; Hasler, D.; Lehmann, R.; Seiler, C.; Forrer, C.; Farrér, C.; Staub, A.; Caduff, M.; Glatthard, J.; Gemperli, A.; Fischer, F.; Rüdisüli, D.; Frasson, V.; Fischer, J.; Bleiker, T. Micro Super Critical Water Oxidation Solids Treatment System. WO2023288329A1, January 19, 2023. https://patents.google.com/patent/WO2023288329A1/en?oq=WO2023288329A1 (accessed 2025-04-15).

(8) *QSDsan/qsdsan/data/sanunit_data/g2rt at toilet · QSD-Group/QSDsan · GitHub*. https://github.com/QSD-Group/QSDsan/tree/toilet/qsdsan/data/sanunit_data/g2rt (accessed 2025-05-02).

(9) *FAOSTAT*. https://www.fao.org/faostat/en/#data/FBS (accessed 2025-03-23).

(10) *Food Energy: Methods of Analysis and Conversion Factors ; Report of a Technical Workshop, Rome, 3 - 6 December 2002*; FAO, Ed.; FAO food and nutrition paper; Food and Agriculture Organization of the United Nations: Rome, 2004.

(11) MacLean, W. C.; Warwick, P. Food and Agriculture Organization of the United Nations: Food Energy: Methods of Analysis and Conversion Factors: Report of a Technical Workshop, Rome, 3–6 December 2002, 2003.

(12) *Guidelines on the Use of Urine and Faeces in Crop Production*; EcoSanRes Programme, 2004.

(13) Holbrook, J. T.; Patterson, K. Y.; Bodner, J. E.; Douglas, L. W.; Veillon, C.; Kelsay, J. L.; Mertz, W.; Smith, J. C. Sodium and Potassium Intake and Balance in Adults Consuming Self-Selected Diets. *Am. J. Clin. Nutr.* **1984**, *40* (4), 786–793. https://doi.org/10.1093/ajcn/40.4.786.

(14) Kodama, N.; Morikuni, E.; Matsuzaki, N.; Yoshioka, Y. H.; Takeyama, H.; Yamada, H.; Kitajima, H.; Nishimuta, M. Sodium and Potassium Balances in Japanese Young Adults. *J. Nutr. Sci. Vitaminol. (Tokyo)* **2005**, *51* (3), 161–168. https://doi.org/10.3177/jnsv.51.161.

(15) Rittmann, B. E.; Mayer, B.; Westerhoff, P.; Edwards, M. Capturing the Lost Phosphorus. *Chemosphere* **2011**, *84* (6), 846–853. https://doi.org/10.1016/j.chemosphere.2011.02.001.

(16) Richert, A.; Gensch, R.; Jönsson, H.; Stenström, T.-A.; Dagerskog, L. Practical Guidance on the Use of Urine in Crop Production. *EcoSanRes Publ. Ser.* **2010**, No. 2010–1.

(17) Turban, S.; Miller, E. R. I.; Ange, B.; Appel, L. J. Racial Differences in Urinary Potassium Excretion. *J. Am. Soc. Nephrol.* **2008**, *19* (7), 1396. https://doi.org/10.1681/ASN.2007101142.

(18) Rose, C.; Parker, A.; Jefferson, B.; Cartmell, E. The Characterization of Feces and Urine: A Review of the Literature to Inform Advanced Treatment Technology. *Crit. Rev. Environ. Sci. Technol.* **2015**, *45* (17), 1827–1879. https://doi.org/10.1080/10643389.2014.1000761.

(19) Wastewater Composition. In *Source Separation and Decentralization for Wastewater Management*; Larsen, T., Udert, K., Lienert, J., Eds.; IWA Publishing, 2013; pp 241–258.

(20) McCarty, P. L.; Bae, J.; Kim, J. Domestic Wastewater Treatment as a Net Energy Producer–Can This Be Achieved? *Environ. Sci. Technol.* **2011**, *45* (17), 7100–7106. https://doi.org/10.1021/es2014264.

(21) D. Orner, K.; R. Mihelcic, J. A Review of Sanitation Technologies to Achieve Multiple Sustainable Development Goals That Promote Resource Recovery. *Environ. Sci. Water Res. Technol.* **2018**, *4* (1), 16–32. https://doi.org/10.1039/C7EW00195A.

(22) Udert, K. M.; Larsen, T. A.; Gujer, W. Biologically Induced Precipitation in Urine-Collecting Systems. *Water Supply* **2003**, *3* (3), 71–78. https://doi.org/10.2166/ws.2003.0010.

(23) Oshita, K.; Kawaguchi ,Koji; Takaoka ,Masaki; Matsukawa ,Kazutsugu; Fujimori ,Takashi; and Fujiwara, T. Emission and Control of N2O and Composition of Ash Derived from Cattle Manure Combustion Using a Pilot-Scale Fluidized Bed Incinerator. *Environ. Technol.* **2016**, *37* (4), 439–445. https://doi.org/10.1080/09593330.2015.1077190.

(24) Winter, F.; Wartha, C.; Hofbauer, H. NO and N2O Formation during the Combustion of Wood, Straw, Malt Waste and Peat. *Bioresour. Technol.* **1999**, *70* (1), 39–49. https://doi.org/10.1016/S0960-8524(99)00019-X.
(25) Fernandez-Lopez, M.; Puig-Gamero, M.; Lopez-Gonzalez, D.; Avalos-Ramirez, A.; Valverde, J.; Sanchez-Silva, L. Life Cycle Assessment of Swine and Dairy Manure: Pyrolysis and Combustion Processes. *Bioresour. Technol.* **2015**, *182*, 184–192. https://doi.org/10.1016/j.biortech.2015.01.140.
(26) Onabanjo, T.; Kolios, A. J.; Patchigolla, K.; Wagland, S. T.; Fidalgo, B.; Jurado, N.; Hanak, D. P.; Manovic, V.; Parker, A.; McAdam, E.; Williams, L.; Tyrrel, S.; Cartmell, E. An Experimental Investigation of the Combustion Performance of Human Faeces. *Fuel* **2016**, *184*, 780–791. https://doi.org/10.1016/j.fuel.2016.07.077.
(27) *Biomass - R&D Technical Reports Archive*. NYSERDA. https://www.nyserda.ny.gov/-/media/Project/Nyserda/Files/Publications/Research/Biomass-Solar-Wind/elemental-analysis-wood-fuel.pdf (accessed 2025-05-30).
(28) Cempa, M.; Olszewski, P.; Wierzchowski, K.; Kucharski, P.; Białecka, B. Ash from Poultry Manure Incineration as a Substitute for Phosphorus Fertiliser. *Materials* **2022**, *15* (9), 3023. https://doi.org/10.3390/ma15093023.
(29) Hedayati, A.; Lindgren, R.; Skoglund, N.; Boman, C.; Kienzl, N.; Öhman, M. Ash Transformation during Single-Pellet Combustion of Agricultural Biomass with a Focus on Potassium and Phosphorus. *Energy Fuels* **2021**, *35* (2), 1449–1464. https://doi.org/10.1021/acs.energyfuels.0c03324.
(30) Tchobanoglous, G.; Stensel, H. D.; Tsuchihashi, R.; Burton, F. In *Wastewater Engineering: Treatment and Resource Recovery*; McGraw-Hill Education: New York, 2013; p 1196.
(31) Dupont. *RO-NF-FilmTec-Manual-45-D01504-En.Pdf*; p 12. https://www.dupont.com/content/dam/water/amer/us/en/water/public/documents/en/RO-NF-FilmTec-Manual-45-D01504-en.pdf (accessed 2025-05-30).
(32) Davey, C. J.; Thomas, N.; McAdam, E. J. Downscaling Reverse Osmosis for Single-Household Wastewater Reuse: Towards Low-Cost Decentralised Sanitation through a Batch Open-Loop Configuration. *Water Reuse* **2022**, *12* (2), 191–205. https://doi.org/10.2166/wrd.2022.084.
(33) Mercer, E.; Davey, C.; Bajón Fernández, Y.; Septien, S.; Tyrrel, S.; Cartmell, E.; Pidou, M.; J. McAdam, E. Membrane Technology for Water Reuse in Decentralised Non-Sewered Sanitation Systems: Comparison of Pressure Driven (Reverse Osmosis) and Thermally Driven Processes (Membrane Distillation and Pervaporation). *Environ. Sci. Water Res. Technol.* **2024**, *10* (11), 2831–2843. https://doi.org/10.1039/D4EW00200H.
(34) *Ultrafiltration Solutions for Dairy & Food Processing*. Membrane System Specialists, Inc. https://www.mssincorporated.com/ultrafiltration/ (accessed 2025-05-30).
(35) Liu, J.; Zhao, M.; Duan, C.; Yue, P.; Li, T. Removal Characteristics of Dissolved Organic Matter and Membrane Fouling in Ultrafiltration and Reverse Osmosis Membrane Combined Processes Treating the Secondary Effluent of Wastewater Treatment Plant. *Water Sci. Technol.* **2020**, *83* (3), 689–700. https://doi.org/10.2166/wst.2020.589.
(36) Yee, S.; Gruendl, H.; Sampl, G.; León, O. E.; Lehmann, R.; Glatthard, J.; Schlauri, M. Human Waste Collection and Separation System, November 9, 2023. https://patentscope.wipo.int/search/en/WO2023214981 (accessed 2025-05-08).
(37) Trimmer, J. T.; Lohman, H. A. C.; Byrne, D. M.; Houser, S. A.; Jjuuko, F.; Katende, D.; Banadda, N.; Zerai, A.; Miller, D. C.; Guest, J. S. Navigating Multidimensional Social–Ecological System Trade-Offs across Sanitation Alternatives in an Urban Informal Settlement. *Environ. Sci. Technol.* **2020**, *54* (19), 12641–12653. https://doi.org/10.1021/acs.est.0c03296.
(38) Gulyas, H.; Zhang, S.; Otterpohl, R. Pretreating Stored Human Urine for Solar Evaporation by Low-Technology Ammonia Stripping. *J. Environ. Prot.* **2014**, *5* (11), 962–969.
(39) Yee, S.; III, D. D.; Holcombe, W. D.; Holmes, J.; Noel, A.; Azevedo, K.; Gaylo, R.; Richter, S.; Turner, T.; Sherman, K.; Hawkins, B.; Miller, G.; Trotochaud, L. Volume Reduction Solids Treatment System. WO2023288327A1, January 19, 2023. https://patents.google.com/patent/WO2023288327A1/en?oq=WO2023288327A1 (accessed 2025-04-15).
(40) Luo, C.; Teng, S.; Wang, J.; Xi, H. Energy Yield from Wastewater by Supercritical Water Oxidation Process: Experimental Validation and Simulation from the Viewpoint of Energy System. *Energy Convers. Manag.* **2024**, *299*, 117876. https://doi.org/10.1016/j.enconman.2023.117876.
(41) Zhang, D.; Clauwaert, P.; Luther, A.; López Barreiro, D.; Prins, W.; (Wim) Brilman, D. W. F.; Ronsse, F. Sub- and Supercritical Water Oxidation of Anaerobic Fermentation Sludge for Carbon and Nitrogen Recovery in a Regenerative Life Support System. *Waste Manag.* **2018**, *77*, 268–275. https://doi.org/10.1016/j.wasman.2018.04.008.
(42) Oe, T.; Suzugaki, H.; Naruse, I.; Quitain, A. T.; Daimon, H.; Fujie, K. Role of Methanol in Supercritical Water Oxidation of Ammonia. *Ind. Eng. Chem. Res.* **2007**, *46* (11), 3566–3573. https://doi.org/10.1021/ie070168u.
(43) Takahashi, Y.; Wydeven, T.; Koo, C. Subcritical and Supercritical Water Oxidation of CELSS Model Wastes. *Adv. Space Res.* **1989**, *9* (8), 99–110. https://doi.org/10.1016/0273-1177(89)90033-1.
(44) Stendahl, K.; Jäfverström, S. Phosphate Recovery from Sewage Sludge in Combination with Supercritical Water Oxidation. *Water Sci. Technol.* **2003**, *48* (1), 185–190. https://doi.org/10.2166/wst.2003.0050.

(45) Yanagida, T.; Minowa, T.; Nakamura, A.; Matsumura, Y.; Noda, Y. Behavior of Inorganic Elements in Poultry Manure during Supercritical Water Gasification. *J. Jpn. Inst. Energy* **2008**, *87* (9), 731–736. https://doi.org/10.3775/jie.87.731.

(46) Acelas, N. Y.; López, D. P.; Brilman, D. W. F. (Wim); Kersten, S. R. A.; Kootstra, A. M. J. Supercritical Water Gasification of Sewage Sludge: Gas Production and Phosphorus Recovery. *Bioresour. Technol.* **2014**, *174*, 167–175. https://doi.org/10.1016/j.biortech.2014.10.003.

(47) Watabe, S. *Advancing the Economic and Environmental Sustainability of the NEWgenerator Nonsewered Sanitation System*; ACS Environ. Au, 2023.

(48) Wong, L. F. A Generalized Learning Curve Adapted for Purchasing and Cost Reduction Negotiations. *Adv. Oper. Res.* **2013**, *2013*, 1–9. https://doi.org/10.1155/2013/584762.

(49) Linton, J. D.; Walsh, S. T. Integrating Innovation and Learning Curve Theory: An Enabler for Moving Nanotechnologies and Other Emerging Process Technologies into Production. *RD Manag.* **2004**, *34* (5), 517–526. https://doi.org/10.1111/j.1467-9310.2004.00359.x.

(50) Yelle, L. E. The Learning Curve: Historical Review and Comprehensive Survey. *Decis. Sci.* **1979**, *10* (2), 302–328. https://doi.org/10.1111/j.1540-5915.1979.tb00026.x.

(51) Watabe, S.; Lohman, H. A. C.; Li, Y.; Morgan, V. L.; Rowles, L. S.; Stephen, T.; Shyu, H.-Y.; Bair, R. A.; Castro, C. J.; Cusick, R. D.; Yeh, D. H.; Guest, J. S. Advancing the Economic and Environmental Sustainability of the NEWgenerator Nonsewered Sanitation System. *ACS Environ. Au* **2023**, *3* (4), 209–222. https://doi.org/10.1021/acsenvironau.3c00001.

(52) *Electricity prices around the world*. GlobalPetrolPrices.com. https://www.globalpetrolprices.com/electricity_prices/ (accessed 2025-05-19).

(53) International Labor Organization. Employment by Sex and Age - ILO Modelled Estimates. ILOSTAT database. https://ilostat.ilo.org/data/ (accessed 2020-06-18).

(54) US EPA, O. *Data and Information Used by WaterSense*. https://www.epa.gov/watersense/data-and-information-used-watersense (accessed 2025-03-23).

(55) AfricaFertilizer.org. *Retail Fertilizer Prices*. AfricaFertilizer. https://africafertilizer.org/local-prices/ (accessed 2019-06-04).

(56) *Even Embers Pellet Patio Heater HTR1085AS*. The Home Depot. https://www.homedepot.com/p/Even-Embers-Pellet-Patio-Heater-HTR1085AS/318982109 (accessed 2025-05-30).

(57) *QSTOVES 106,000 BTU Steel Pellet Heater Black Softwood Pellet Fuel Patio Heater Q05X*. The Home Depot. https://www.homedepot.com/p/QSTOVES-106-000-BTU-Steel-Pellet-Heater-Black-Softwood-Pellet-Fuel-Patio-Heater-Q05X/326738769 (accessed 2025-05-30).

(58) *40-lb Heating Wood Pellets Lowes.com*. Lowe's. https://www.lowes.com/pd/40-lb-Heating-Wood-Pellets-100-Natural-High-BTU-s-Low-Ash/5001965889 (accessed 2025-05-30).

(59) *Amazon.com : Camp Chef Competition Blend BBQ Pellets, Hardwood Pellets for Grill, Smoke, Bake, Roast, Braise and BBQ, 20 lb. Bag : Patio, Lawn & Garden*. https://www.amazon.com/Camp-Chef-Competition-Blend-Pellets/dp/B01CQ2VSBO/ref=sr_1_3?crid=54BLJMDLU24O&dib=eyJ2IjoiMSJ9.w0rqH86rDKyV_MdHJ4ULIBDS1t0O_Tju0YI4o3_cJ_N_fc-NvUN7He8-KPRR_9GcQGPgVOkChwpJdo8os6qH7w9xZVaXCzA-EtwWccxbAAq8fwpyY1eTZUDyqnsDaVDzlsUgKQVOhR-1tRTHftCsR880qZzJheTVVMTr6I7hcqUMwO8PS5W256L4a-cZ0AcJQRtI_LLCOoYaMGoOhkIL9YedGK2SzAp6ivDHkTWOqXh4C_8LRe7uTrO1GpMdNmTfH6d-7Ctv0vxNSiBAKn071MeLKtss4vd_yoKKgYjZBKg.pbSambplxBL-Fi-lfBtQxUxq0_KOwOn7xDS7-hLNLfg&dib_tag=se&keywords=wood%2Bpellets&qid=1731689544&s=home-garden&sprefix=wood%2Bpellets%2Cgarden%2C132&sr=1-3&th=1 (accessed 2025-05-30).

(60) *Soft Wood Fuel Pellets, 40 lb. at Tractor Supply Co*. Tractor Supply Company. https://www.tractorsupply.com/tsc/product/wise-wood-wood-pellet-fuel-40-lb-bag (accessed 2025-05-30).

(61) *Amazon.com : recteq Ultimate Premium Hardwood Grilling Cooking Pellet Barbecue BBQ Grill Smoker Blend with Red Oak, White Oak, and Hickory, 40 Pound Bag : Patio, Lawn & Garden*. https://www.amazon.com/RecTec-Grills-Ultimate-Blend-Pellets/dp/B01DDI0WX0/ref=sr_1_5?crid=54BLJMDLU24O&dib=eyJ2IjoiMSJ9.w0rqH86rDKyV_MdHJ4ULIBDS1t0O_Tju0YI4o3_cJ_N_fc-NvUN7He8-KPRR_9GcQGPgVOkChwpJdo8os6qH7w9xZVaXCzA-EtwWccxbAAq8fwpyY1eTZUDyqnsDaVDzlsUgKQVOhR-1tRTHftCsR880qZzJheTVVMTr6I7hcqUMwO8PS5W256L4a-cZ0AcJQRtI_LLCOoYaMGoOhkIL9YedGK2SzAp6ivDHkTWOqXh4C_8LRe7uTrO1GpMdNmTfH6d-7Ctv0vxNSiBAKn071MeLKtss4vd_yoKKgYjZBKg.pbSambplxBL-Fi-lfBtQxUxq0_KOwOn7xDS7-hLNLfg&dib_tag=se&keywords=wood+pellets&qid=1731689544&s=home-garden&sprefix=wood+pellets%2Cgarden%2C132&sr=1-5 (accessed 2025-05-30).

(62) *Low Profile Turbine Ventilator(10 Inch Aluminum)*. HVAC Express. https://hvacexpress.com/product/low-profile-turbine-ventilator-10-inch-aluminum/ (accessed 2025-05-30).

(63) *Commercial Reverse Osmosis System | 200-300 GPD | Crystal Quest*. Crystal Quest Water Filters. https://crystalquest.com/products/reverse-osmosis-system-low-flow (accessed 2025-06-02).

(64) *AXEON HF1-2521 400 GPD RO Membrane (200375)*. Reverse Osmosis Superstore. https://www.reverseosmosis.com/products/axeon-hf1-2521-400-gpd-150-psi-ro-membrane-element-200375 (accessed 2025-06-02).
(65) *McMaster-Carr*. https://www.mcmaster.com/catalog/131/2384/7426N19 (accessed 2025-06-02).
(66) Trotochaud, L.; Andrus, R. M.; Tyson, K. J.; Miller, G. H.; Welling, C. M.; Donaghy, P. E.; Incardona, J. D.; Evans, W. A.; Smith, P. K.; Oriard, T. L.; Norris, I. D.; Stoner, B. R.; Guest, J. S.; Hawkins, B. T. Laboratory Demonstration and Preliminary Techno-Economic Analysis of an Onsite Wastewater Treatment System. *Environ. Sci. Technol.* **2020**, *54* (24), 16147–16155. https://doi.org/10.1021/acs.est.0c02755.
(67) *Amazon.com: Moonshan Belt Conveyor 39 inch Conveyor Desktop Version Step-Less Speed Adjustment PVC Belt Conveyor for Packaging Factory, Production Line, Home : Industrial & Scientific*. https://www.amazon.com/Moonshan-Step-Less-Adjustment-Packaging-Production/dp/B0CCP8NSSJ (accessed 2025-06-03).
(68) *Ptfe Fiberglass Mesh Conveyor Belt Manufacturer For Conveyor Belt Oven - Buy Ptfe Food Grade Mesh Belt ptfe Teflonning Mesh Belt mesh Conveyor Belt Heat Insulation Ptfe Coated Fib Product on Alibaba.com*. www.alibaba.com. https://www.alibaba.com/product-detail/Ptfe-Fiberglass-Mesh-Conveyor-Belt-Manufacturer_1600087818610.html (accessed 2025-06-03).
(69) *Customized Teflon Mesh Conveyor Belt for Films Shrink Tunnel Sealing Machine*. eBay. https://www.ebay.com/itm/226018713201 (accessed 2025-06-03).
(70) *Heavy Duty Commercial Media Funnel 4" or cut to turn into 6" Tank OpeningsDefault Title*. PURE DIRECT USA. https://www.puredirectusa.com/products/heavy-duty-commercial-media-funnel-4-or-cut-to-turn-into-6-tank-openings (accessed 2025-06-03).
(71) *Laboratory Use Plate And Frame Filter Press - Buy Frame Filter Press laboratory Use Filter Press plate And Frame Filter Press Product on Alibaba.com*. www.alibaba.com. https://www.alibaba.com/product-detail/Laboratory-Use-Plate-and-Frame-Filter_1600443614071.html (accessed 2025-06-03).
(72) *110V Stainless Steel Plate and Frame Press Filtration Solid Liquid Filter Ft-150*. eBay. https://www.ebay.com/itm/374953006026 (accessed 2025-06-03).
(73) *Plate And Frame Filter Press Machine For Stone Mining Tailings Wastewater Treatment - Buy Plate And Frame Filter Press Machine For Kaolin Ceramic Clay Filter Press Machine For Granite Marble Stone Industries Gold Mining Wastewater Treatment plate And Frame Filter Press Machine For Edible Oil Product on Alibaba.com*. www.alibaba.com. https://www.alibaba.com/product-detail/Plate-and-Frame-Filter-Press-Machine_60777426971.html (accessed 2025-06-03).
(74) *Filter Press Brewery For Waste Water - Buy Filter Press filter Press Brewery filter Press Product on Alibaba.com*. www.alibaba.com. https://www.alibaba.com/product-detail/Filter-Press-Brewery-for-Waste-Water_62221978178.html (accessed 2025-06-03).
(75) *4 11/16 in Ht, 1 1/2 in Dp, Standard Square Axial Fan*. Grainger. https://www.grainger.com/product/DAYTON-Standard-Square-Axial-Fan-2RTJ7 (accessed 2025-06-03).
(76) *McMaster-Carr*. https://www.mcmaster.com/5865N129/ (accessed 2025-06-03).
(77) *15 Gallon Natural LLDPE Rectangular Tamco® Heavy-Duty Tank with Internal Flange - 24" L x 12" W x 12" Hgt. (Cover Sold Separately) | U.S. Plastic Corp.* https://www.usplastic.com/catalog/item.aspx?itemid=26176&v1=&v7=&gad_source=1&gclid=Cj0KCQjwo8S3BhDeARIsAFRmkONlUWVFApK_ro6DWZRtCnTtQSR4VtFGrNOROBhzbksKy8FwFRxup5MaAjiREALw_wcB (accessed 2025-06-03).
(78) *McMaster-Carr*. https://www.mcmaster.com/3687K103/ (accessed 2025-06-03).
(79) *McMaster-Carr*. https://www.mcmaster.com/1792N16/ (accessed 2025-06-03).
(80) *McMaster-Carr*. https://www.mcmaster.com/4439T14/ (accessed 2025-06-03).
(81) *LWD, 4,000 W, Resistored LWD Element*. Grainger. https://www.grainger.com/product/RHEEM-Resistored-LWD-Element-LWD-15A574 (accessed 2025-06-03).
(82) *DC-motors 12VDC 15.5A 2600rpm 134W. - Transmotec*. https://www.transmotec.com/product/D10079-12/ (accessed 2025-06-03).
(83) Rowles, L. S. Financial Viability and Environmental Sustainability of Fecal Sludge Treatment with Pyrolysis Omni Processors. *ACS Env. Au* **2022**, *2*, 455–466.
(84) *McMaster-Carr*. https://www.mcmaster.com/8701K95/ (accessed 2025-06-03).
(85) *Solid Shaft, 1 1/2 in Dia, Linear Shaft*. Grainger. https://www.grainger.com/product/THOMSON-Linear-Shaft-Solid-Shaft-3DWT7 (accessed 2025-06-03).
(86) *Self-Priming Pumps | Pump World*. https://www.pumpworld.com/categories/6858/self-priming-pumps (accessed 2025-06-03).
(87) *26.2 gal, 48 in x 22 1/2 in x 7 1/4 in, Straight Wall Container*. Grainger. https://www.grainger.com/product/ORBIS-Straight-Wall-Container-26-3CLW4 (accessed 2025-06-03).
(88) *Master Flow 36 in. x 48 in. Galvanized-Steel Flat Sheet - 30-Gauge Duct GFS36X48*. The Home Depot. https://www.homedepot.com/p/Master-Flow-36-in-x-48-in-Galvanized-Steel-Flat-Sheet-30-Gauge-Duct-GFS36X48/100175914 (accessed 2025-06-03).

(89) *Master Flow 12 in. x 8 in. x 4 ft. Half Section Rectangular Duct RD12X8X48*. The Home Depot. https://www.homedepot.com/p/Master-Flow-12-in-x-8-in-x-4-ft-Half-Section-Rectangular-Duct-RD12X8X48/100185410 (accessed 2025-06-03).
(90) *Heating & Cooling Products 10" x 24" x 48" 26 Gauge Rectangular Metal Duct Pipe (Half Section)*. https://www.menards.com/main/heating-cooling/ductwork/ductwork-pipe/heating-cooling-products-26-gauge-rectangular-metal-duct-pipe/15342450hb/p-1444432235537-c-14259.htm (accessed 2025-06-03).
(91) *Round Compact Axial Fans - Grainger Industrial Supply*. https://www.grainger.com/category/hvac-and-refrigeration/cooling-fans/equipment-cooling-axial-fans/compact-axial-fans/round-compact-axial-fans?filters=webParentSkuKey&searchQuery=evaporator+fan&webParentSkuKey=WP10610196&sst=4&ts_optout=true (accessed 2025-06-03).
(92) *Square Compact Axial Fans - Grainger Industrial Supply*. https://www.grainger.com/category/hvac-and-refrigeration/cooling-fans/equipment-cooling-axial-fans/compact-axial-fans/square-compact-axial-fans?filters=webParentSkuKey&searchQuery=evaporator+fan&webParentSkuKey=WP10610193&sst=4&ts_optout=true (accessed 2025-06-03).
(93) *VEVOR Belt Conveyor, 47 x 7.8 inch Conveyor Table, Heavy Duty Stainless Steel Motorized Belt Conveyor for Inkjet Coding Applications Powered PVC Belt Anti-Static Adjustable Speed (Double Guardrail) | VEVOR US*. Vevor. https://www.vevor.com/belt-conveyor-c_10439/vevor-belt-conveyor-47-x-7-8-inch-conveyor-table-heavy-duty-stainless-steel-motorized-belt-conveyor-for-inkjet-coding-applications-powered-pvc-belt-anti-static-adjustable-speed-double-guardrail--p_010728062662 (accessed 2025-06-03).
(94) *McMaster-Carr*. https://www.mcmaster.com/products/heat-wrap/high-temperature-constant-wattage-heaters-for-pipes-and-tubes/ (accessed 2025-06-03).
(95) *McMaster-Carr*. https://www.mcmaster.com/products/heat-wrap/ultra-flexible-constant-wattage-heaters-for-pipes-and-tubes/ (accessed 2025-06-03).
(96) *McMaster-Carr*. https://www.mcmaster.com/products/heat-wrap/cut-to-length-self-regulating-heaters-for-pipes-and-tubes-9/ (accessed 2025-06-03).
(97) *McMaster-Carr*. https://www.mcmaster.com/products/heat-wrap/ultra-high-temperature-constant-wattage-heaters-for-pipes-and-tubes/ (accessed 2025-06-03).
(98) *80W Silicone Heating Belt Waterproof Flexible Heater Wrap Self Control Tempe BEL 7596790928714*. eBay. https://www.ebay.com/itm/315362886164 (accessed 2025-06-03).
(99) *Heating Blankets, Tapes, & Cords - Grainger Industrial Supply*. https://www.grainger.com/search/lab-supplies/lab-heating/heating-blankets-tapes-cords?filters=webParentSkuKey&searchQuery=heating+wrap&webParentSkuKey=WP15703011%7CWP15703017&sst=4&tv_optin=true&gwwRemoveElement=true (accessed 2025-06-03).
(100) *Xnrtop 3 Pcs K Type Temperature Sensor Thermocouple Probe Copper Wire 5 Meter 16.4Ft: Amazon.com: Industrial & Scientific*. https://www.amazon.com/Xnrtop-Temperature-Sensor-Thermocouple-Copper/dp/B07PFX1CXJ?source=ps-sl-shoppingads-lpcontext&ref_=fplfs&psc=1&smid=A4EE8R8C5DE8H (accessed 2025-06-03).
(101) *Honeywell 18" Thermocouple*. https://www.menards.com/main/heating-cooling/heaters/garage-heater-replacement-parts-accessories/honeywell-18-thermocouple/cq100a1021/p-1444433406354-c-6860.htm (accessed 2025-06-03).
(102) *White Rodgers 36 in. Universal Replacement Thermocouple TC36*. The Home Depot. https://www.homedepot.com/p/White-Rodgers-36-in-Universal-Replacement-Thermocouple-TC36/206087727 (accessed 2025-06-03).
(103) www.supplyhouse.com, S. *H06E-36 - White Rodgers H06E-36 - 36" Universal Replacement Thermocouple*. SupplyHouse.com. https://www.supplyhouse.com/White-Rodgers-H06E-36-36-Universal-Replacement-Thermocouple (accessed 2025-06-03).
(104) *McMaster-Carr*. https://www.mcmaster.com/products/thermocouples/thermocouples-1~/thermocouple-probes-with-armored-cable-for-liquids-and-gases/ (accessed 2025-06-03).
(105) *McMaster-Carr*. https://www.mcmaster.com/products/heater-temperature-controls/on-off-temperature-controllers-7/ (accessed 2025-06-03).
(106) *Rheem PROTECH 3 1/4 in. Standard Shank Water Heater Temperature and Pressure Relief Valve SP12574*. The Home Depot. https://www.homedepot.com/p/Rheem-PROTECH-3-1-4-in-Standard-Shank-Water-Heater-Temperature-and-Pressure-Relief-Valve-SP12574/205651345 (accessed 2025-06-03).
(107) *110VAC Stainless Steel Pilot Operated Diaphragm Solenoid Valve, Normally Closed, 1" NPT Pipe Size*. https://www.boltontool.com/index.php?route=product/product&product_id=31694&srsltid=AfmBOooWJahASKdTNLYueVAdoFv6JNK-s_zcYzKWGTndJHwNMo8NQkt_1sY%20https://valveman.com/products/stc2v130-3-8-solenoid-valve-3-8-npt-2-way-normally-closed-pilot-operated-diaphragm/?srsltid=AfmBOooFTsmGzdIdoQ1mqSkn6h2PwZyNH82Xey-RiXNZN-U3BcfXYxcSbBo (accessed 2025-06-03).
(108) *Solenoid Valve, Pilot Operated 1/2" | Brass*. King Supply Company. https://www.kingsupply.com/products/1-2-solenoid-valve-pilot-operated-brass (accessed 2025-06-03).

(109) *OLAB 110/120 volt solenoid water valve 1/2" NPT*. Mister Valve. https://mistervalve.com/en/home/olab-110120-volt-solenoid-water-valve-12-npt.html?srsltid=AfmBOopbc9HS1ByKXcyArx-l01cTKeK-t2b9f-HyjnTk-rSasuR85PfrlnM (accessed 2025-06-03).
(110) *High Pressure Solenoid Valve - 1/4" Stainless Steel 60 bar, 24V DC Hig*. U.S. Solid. https://ussolid.com/products/high-pressure-solenoid-valve-1-4-stainless-steel-60-bar-24v-dc-high-pressure-high-temperature-resistance-solenoid-valve-356-html (accessed 2025-06-03).
(111) *U.S. Solid 3/4" Brass Electric Solenoid Valve 110V AC Normally Closed Non-potable Water, air, Diesel: Industrial Solenoid Valves: Amazon.com: Industrial & Scientific*. https://www.amazon.com/Electric-Solenoid-Normally-Closed-diesel/dp/B007N0J98E?source=ps-sl-shoppingads-lpcontext&ref_=fplfs&smid=A20QEF2LEKCK8G&th=1 (accessed 2025-06-03).
(112) *STC 2V130- 3/8" Solenoid Valve 2-Way, Normally Closed, Pilot-Operated Diaphragm*. ValveMan.com. https://valveman.com/products/stc2v130-3-8-solenoid-valve-3-8-npt-2-way-normally-closed-pilot-operated-diaphragm/ (accessed 2025-06-03).
(113) *McMaster-Carr*. https://www.mcmaster.com/6622K18-6622K183/ (accessed 2025-06-03).
(114) *Stainless Steel High Pressure Reactor Autoclave Vessel 22MPa 350°C Heating Temp*. eBay. https://www.ebay.com/itm/334132934833 (accessed 2025-06-03).
(115) *McMaster-Carr*. https://www.mcmaster.com/3347K56/ (accessed 2025-06-03).
(116) *McMaster-Carr*. https://www.mcmaster.com/3347K32/ (accessed 2025-06-03).
(117) *McMaster-Carr*. https://www.mcmaster.com/1810T2-1810T26/ (accessed 2025-06-03).
(118) *McMaster-Carr*. https://www.mcmaster.com/82695K93/ (accessed 2025-06-03).
(119) *e128 Electric Metering Pump*. Hog Slat. https://www.hogslat.com/e128-electric-metering-pump (accessed 2025-06-03).
(120) *12 gpd Max. Flow Rate, 1/60 hp Max. Horsepower, Chemical Metering Pump*. Grainger. https://www.grainger.com/product/MEC-O-MATIC-Chemical-Metering-Pump-12-20LX55 (accessed 2025-06-03).
(121) Seider, W. D.; Lewin, D. R.; Seader, J. D.; Widagdo, S.; Gani, R.; Ng, M. K. Cost Accounting and Capital Cost Estimation. In *Product and Process Design Principles*; Wiley, 2017; pp 426–485.
(122) *Dive Systems Pro Alu 0,5L Harness MiniDiveCompressor MiniComp*. COM MOVESEA. https://movesea.com/products/dive-systems-minidive-pro-alu-0-5l-harness (accessed 2025-06-03).
(123) *Air Venturi RovAir 4500 Portable Compressor | Pyramyd AIR*. https://www.pyramydair.com/product/air-venturi-rovair-4500-portable-compressor?a=12619 (accessed 2025-06-03).
(124) *TUXING 4500Psi PCP Air Compressor,Scuba Tank Diving Compressor for Filling Pcp Air Rifles,Scuba Diving Tanks*. Walmart.com. https://www.walmart.com/ip/TUXING-4500Psi-PCP-Air-Compressor-Scuba-Tank-Diving-Compressor-Double-Cylinder-High-Pressure-Pump-Adjustable-Pressure-Filling-Pcp-Air-Rifles-Scuba-Di/5381275306 (accessed 2025-06-03).
(125) *Amazon.com: PRECIPETTE Vacuum Pump 27L/Min with Switch, Oilless Diaphragm with Silencer, 6m PTFE Tape & 1m Rubber Tube, Low Noise & High Vacuum Degree, for Lab Electronics Aquarium : Industrial & Scientific*. https://www.amazon.com/Diaphragm-Silencer-Chemistry-Electronics-Aquarium/dp/B0BYJFJ3CN?source=ps-sl-shoppingads-lpcontext&ref_=fplfs&smid=A1W522WQCRBUSP&th=1 (accessed 2025-06-03).
(126) *0.5A Oil Free Diaphragm Vacuum Pump For Vacuum Filtration Lab Tool 30L/min*. eBay. https://www.ebay.com/itm/386182582193 (accessed 2025-06-03).
(127) *Amazon.com: JEGS Electric Vacuum Pump Kit | Provides Between 18” - 22” Hg of Vacuum | Includes Vacuum Pump, Hose, Switch & Pigtail, Vibration Isolators, 10 Amp Fuse, 12 Volt DC / 84 Watt Relay, and Hardware : Automotive*. https://www.amazon.com/JEGS-63016-Electric-Vacuum-Pump/dp/B078WK4XT9?source=ps-sl-shoppingads-lpcontext&ref_=fplfs&psc=1&smid=A30XU5Y510T9EB (accessed 2025-06-03).
(128) *LAB FISH Laboratory Vacuum Pump Portable Oilless Diaphragm Lab Vacuum Filtration 15L/min with Rubber Tube,110V-220V: Amazon.com: Industrial & Scientific*. https://www.amazon.com/Portable-Oilless-Diaphragm-Vacuum-Rubber/dp/B08GWGCCL2?source=ps-sl-shoppingads-lpcontext&ref_=fplfs&th=1 (accessed 2025-06-03).
(129) *Summit Racing SUM-760152 Summit Racing™ 12v Electric Vacuum Pumps | Summit Racing*. Summit Racing Equipment. https://www.summitracing.com/parts/sum-760152 (accessed 2025-06-03).
(130) *Always in Stock - Cole-Parmer PTFE-Coated Vacuum Pump, Gauge/Reg/Valve; 0.75 cfm/23.2"Hg-25psi/115V from Cole-Parmer*. https://www.coleparmer.com/i/cole-parmer-ptfe-coated-vacuum-pump-gauge-reg-valve-0-75-cfm-23-2-hg-25psi-115v/7920000?PubID=UX&persist=true&ip=no&srsltid=AfmBOoqBsKFCEQe3DWRnPzoFIpeu99vET19FPB7jD2HQeutflGnBcyvzSJ4%20https://www.rockler.com/rockler-vacuum-pump?country=US&promo=shopping (accessed 2025-06-03).
(131) *Mesh, 155 mesh, Strainer Screen*. Grainger. https://www.grainger.com/product/AMIAD-Strainer-Screen-Mesh-1PHP7 (accessed 2025-06-03).

(132) *Water Source 60 Mesh Sediment Filter Replacement Screen SFS-60*. The Home Depot. https://www.homedepot.com/p/Water-Source-60-Mesh-Sediment-Filter-Replacement-Screen-SFS-60/203990074 (accessed 2025-06-03).
(133) *Amazon.com: PATIKIL 40 Mesh in-Line Strainer Replacement, 1 Pair Stainless Steel Filter Screen Accessories for Garden Hose Water Pump Pressure Washer : Patio, Lawn & Garden*. https://www.amazon.com/PATIKIL-Strainer-Replacement-Stainless-Accessories/dp/B0D8GDC4GF?source=ps-sl-shoppingads-lpcontext&ref_=fplfs&psc=1&smid=AF9CPILHGHOMP (accessed 2025-06-03).
(134) *Spin Clean Y-Filter Replacement Element*. https://www.dripdepot.com/replacement-filter-screen-for-spin-clean-filters-filtration-150-micron-100-mesh-filter-size-three-quarter-inch-1-inch (accessed 2025-06-03).
(135) *Rusco Spin-Down Element - 40 Mesh*. Agri Sales Inc. https://www.agrisales-inc.com/rusco-spin-down-element-40-mesh (accessed 2025-06-03).
(136) *DERNORD 100 Mesh Stainless Steel Screen for 1.5"/2" Filter Tri-Clamp Sanitary SS304 Inline Straight Strainer: Amazon.com: Industrial & Scientific*. https://www.amazon.com/DERNORD-Stainless-Tri-Clamp-Sanitary-Straight/dp/B07XC47K2J?source=ps-sl-shoppingads-lpcontext&ref_=fplfs&smid=A1USG81SWV090L&th=1 (accessed 2025-06-03).
(137) *Damper, Floating Point with Timeout/On/Off, Actuator*. Grainger. https://www.grainger.com/product/JOHNSON-CONTROLS-Actuator-Damper-41P464 (accessed 2025-06-03).
(138) *Saniflo SaniPlus 0.5 HP 115-Volt Macerator Effluent Septic Pump 002*. The Home Depot. https://www.homedepot.com/p/Saniflo-SaniPlus-0-5-HP-115-Volt-Macerator-Effluent-Septic-Pump-002/206262151 (accessed 2025-06-03).
(139) *Departments - JOHNSON PUMP MACERATOR THERMAL PROTECTED MOTOR 24V*. https://shop.hamiltonmarine.com/products/pump-macerator-24v-thermal-protected-motor-51861.html (accessed 2025-06-03).
(140) *RecPro 12V RV Macerator Pump*. RecPro. https://recpro.com/recpro-12v-rv-macerator-pump/ (accessed 2025-06-03).
(141) *RV Macerator Pump 12V,12GPM Suction 3.2 feet Lift 10 feet with Garden Hose Discharge Port,Quick Release Sewage Pump with Fresh Water Rinse and Manual Crushing Function,for Boat Marine RV Campe - Amazon.com*. https://www.amazon.com/RV-Macerator-Pump-Discharge-crushing/dp/B0CZMGGYPX?source=ps-sl-shoppingads-lpcontext&ref_=fplfs&smid=A370615CNM9I6P&th=1 (accessed 2025-06-03).
(142) *VEVOR RV Portable Macerator Pump, 12V, 12GMP Quick Release RV Waste Pump, RV Sewage Sewer Pump with Detachable Quick Connection Valve Metal Hose Clamp Power Cord, for RV Boat Marine Motorhome Camper | VEVOR US*. Vevor. https://www.vevor.com/water-pumps-accessories-c_12381/vevor-rv-portable-macerator-pump-12v-12gmp-quick-release-rv-waste-pump-rv-sewage-sewer-pump-with-detachable-quick-connection-valve-metal-hose-clamp-power-cord-for-rv-boat-marine-motorhome-camper-p_010256051288 (accessed 2025-06-03).
(143) *2-1/2 Gallon Natural LLDPE Rectangular Utility Tamco® Tank with 4" Plain Lid & 1/2" FNPT Fitting & 1/2-Gallon Graduations - 12" L x 8" W x 10" Hgt. | U.S. Plastic Corp.* https://www.usplastic.com/catalog/item.aspx?itemid=134147&v1=&v7=&gad_source=1&gclid=Cj0KCQjw3vO3BhCqARIsAEWblcB-oV85uG-0iiOzC1TMkaeqhkYyZQ-oi8jw3kQqSO7BABHGvhcEfeEaAsQhEALw_wcB (accessed 2025-06-03).
(144) *McMaster-Carr*. https://www.mcmaster.com/4439T15/ (accessed 2025-06-03).
(145) *Liquid Storage Tanks - Grainger Industrial Supply*. https://www.grainger.com/category/material-handling/storage-workspace/drums-pails-ibc-totes-tanks/liquid-storage-tanks-ibc-totes (accessed 2025-06-03).
(146) *Class A Customs 20 Gallon Fresh / Gray Water Holding Tank classAcustoms MADE IN USA*. Class A Customs. https://www.classacustoms.com/20-Gallon-Fresh-Gray-Water-Holding-Tank_p_34.html (accessed 2025-06-03).
(147) *Amazon.com : 12 Gallon Portable Water Tank with Spigot&Hose, Water Storage Container for Camping, RV, Truck, Emergency Preparedness （Thickening） : Sports & Outdoors*. https://www.amazon.com/Water-Containers-Emergency-Household-Self-Driving/dp/B0C3HKFH8M?th=1 (accessed 2025-06-03).
(148) *Norwesco 20-Gallons Plastic White Water Storage Tank Lowes.com*. Lowe’s. https://www.lowes.com/pd/Norwesco-20-Gallon-Flat-Bottom-Utility-Tank/5015428555 (accessed 2025-06-03).
(149) *Vertical Storage Tanks | 10 - 120 Gallons » BENECOR*. BENECOR. https://benecor.com/product/vertical-storage-tanks-10-120-gallons/ (accessed 2025-06-03).
(150) *20 Gallon White Vertical Storage Tank | Ace VT0020-16*. https://www.ntotank.com/20gallon-acerotomold-white-heavyduty-vertical-storage-tank-x7030195 (accessed 2025-06-03).
(151) *McMaster-Carr*. https://www.mcmaster.com/1972T325/ (accessed 2025-06-03).
(152) *McMaster-Carr*. https://www.mcmaster.com/5065K34/ (accessed 2025-06-03).

(153) Chang, H.; Zhao, Y.; Xu, A.; Damgaard, A.; Christensen, T. H. Mini-Review of Inventory Data for the Dewatering and Drying of Sewage Sludge. *Waste Manag. Res.* **2023**, *41* (6), 1081–1088. https://doi.org/10.1177/0734242X221139170.
(154) *Sludge Drying | WendeWolf® Sludge Dryer System - MAK Water*. https://www.makwater.com.au/solutions/wendewolf-solar-sludge-drying (accessed 2025-06-17).
(155) Miller, A.; Espanani, R.; Junker, A.; Hendry, D.; Wilkinson, N.; Bollinger, D.; Abelleira-Pereira, J. M.; Deshusses, M. A.; Inniss, E.; Jacoby, W. Supercritical Water Oxidation of a Model Fecal Sludge without the Use of a Co-Fuel. *Chemosphere* **2015**, *141*, 189–196. https://doi.org/10.1016/j.chemosphere.2015.06.076.
(156) Zhang, F.; Li, Y.; Liang, Z.; Wu, T. Energy Conversion and Utilization in Supercritical Water Oxidation Systems: A Review. *Biomass Bioenergy* **2022**, *156*, 106322. https://doi.org/10.1016/j.biombioe.2021.106322.
(157) *Saniflo 082 Saniaccess 3-Macerator Effluent Septic Pump (1/2 HP) 082*. The Home Depot. https://www.homedepot.com/p/Saniflo-082-Saniaccess-3-Macerator-Effluent-Septic-Pump-1-2-HP-082/204204411 (accessed 2025-06-03).
(158) *PILOT Series Chemical Resistant Diaphragm Vacuum Pumps*. The Lab Depot. https://www.labdepotinc.com/p-62092-pilot5000-chemical-resistant-diaphragm-vacuum-pump (accessed 2025-06-03).
(159) Perry, R. H.; Green, D. W. Thermodynamic Analysis of Processes. In *Perry's Chemical Engineers' Handbook*; McGraw-Hill Professional: New York, NY, 1997; pp 4–36.
(160) *Fans - Efficiency and Power Consumption*. https://www.engineeringtoolbox.com/fans-efficiency-power-consumption-d_197.html (accessed 2025-06-03).
(161) Aggarwal, N. *Sherwood Number Calculator – Significance and Calculation*. https://chemenggcalc.com/sherwood-number-calculator/ (accessed 2025-06-03).
(162) *Filter Press Replacement with Zeta Plus^{TM} Depth Filter Cartridges for Beer Clarification*. https://multimedia.3m.com/mws/media/2113780O/3m-zeta-plus-vs-filter-press-for-beer-clarification-application-brief.pdf (accessed 2025-06-03).
(163) *International - U.S. Energy Information Administration (EIA)*. https://www.eia.gov/international/data/world/electricity/electricity-generation?pd=2&p=0000000000000000000000000000000fvu0000000000000000000000000000000u&u=0&f=A&v=mapbubble&a=-&i=none&vo=value&t=C&g=000000000000000000000000000000000000000000000000000001&l=249-ruvvvvvfvtvnvv1vrvvvvfvvvvvvfvvvou20evvvvvvvvvvnvvvs0008&s=315532800000&e=1672531200000 (accessed 2025-03-23).
(164) *ecoinvent Version 3.8*. https://support.ecoinvent.org/ecoinvent-version-3.8 (accessed 2025-03-23).
(165) IPCC. *2019 Refinement to the 2006 IPCC Guidelines for National Greenhouse Gas Inventories, Volume 4*; 2019. https://www.ipcc-nggip.iges.or.jp/public/2019rf/vol4.html (accessed 2022-06-13).
(166) IPCC. *2006 IPCC Guidelines for National Greenhouse Gas Inventories, Volume 4*; 2006. https://www.ipcc-nggip.iges.or.jp/public/2006gl/vol4.html (accessed 2022-06-13).
(167) *tap water production, underground water with chemical treatment - Rest-of-World - tap water | ecoQuery*. https://ecoquery.ecoinvent.org/3.8/cutoff/dataset/11413/documentation (accessed 2025-04-20).
(168) *urea production - Rest-of-World - urea | ecoQuery*. https://ecoquery.ecoinvent.org/3.8/cutoff/dataset/5039/documentation (accessed 2025-04-20).
(169) *triple superphosphate production - Rest-of-World - triple superphosphate | ecoQuery*. https://ecoquery.ecoinvent.org/3.8/cutoff/dataset/23091/documentation (accessed 2025-04-20).
(170) *potassium chloride production - Rest-of-World - potassium chloride | ecoQuery*. https://ecoquery.ecoinvent.org/3.8/cutoff/dataset/8138/documentation (accessed 2025-04-20).
(171) Feng, J.; Li, Y.; Strathmann, T. J.; Guest, J. S. Characterizing the Opportunity Space for Sustainable Hydrothermal Valorization of Wet Organic Wastes. *Environ. Sci. Technol.* **2024**, *58* (5), 2528–2541. https://doi.org/10.1021/acs.est.3c07394.
(172) Tow, E. W.; Hartman, A. L.; Jaworowski, A.; Zucker, I.; Kum, S.; AzadiAghdam, M.; Blatchley, E. R.; Achilli, A.; Gu, H.; Urper, G. M.; Warsinger, D. M. Modeling the Energy Consumption of Potable Water Reuse Schemes. *Water Res. X* **2021**, *13*, 100126. https://doi.org/10.1016/j.wroa.2021.100126.
(173) *Energy Conservation Measures | Water and Energy Conservation Guidance Manual for Sewage Works | ontario.ca*. http://www.ontario.ca/document/water-and-energy-conservation-guidance-manual-sewage-works/energy-conservation-measures (accessed 2025-07-02).
(174) Lohman, H. A. C.; Li, Y.; Zhang, X.; Morgan, V. L.; Watabe, S.; Rowles, L. S.; Cusick, R. D.; Guest, J. S. Defining Economic and Environmental Typologies across 77 Countries to Prioritize Opportunities for Nonsewered Sanitation. *Environ. Sci. Technol.* **2025**. https://doi.org/10.1021/acs.est.5c02064.
(175) Rahman, A.; Feng, J.; Kala, V.; O'Brien, W.; Rai, S.; Mendoza, J. A.; Guest, J.; Cusick, R. The Potential of Non-Sewered Sanitation (NSS) in Urban Settings: Exploring Core Value Propositions across Global and Local Contexts. ChemRxiv September 29, 2025. https://doi.org/10.26434/chemrxiv-2025-16tmw.

(176) Lohman, H.; Li, Y.; Zhang, X.; Morgan, V.; Watabe, S.; Rowles, L. S.; Cusick, R.; Guest, J. Defining Economic and Environmental Typologies across 77 Countries to Prioritize Opportunities for Non-Sewered Sanitation. ChemRxiv January 21, 2025. https://doi.org/10.26434/chemrxiv-2025-zb1r0.
